# One-pot synthesis of diaryliodonium salts from arenes and aryl iodides with Oxone–sulfuric acid

**DOI:** 10.3762/bjoc.14.70

**Published:** 2018-04-12

**Authors:** Natalia Soldatova, Pavel Postnikov, Olga Kukurina, Viktor V Zhdankin, Akira Yoshimura, Thomas Wirth, Mekhman S Yusubov

**Affiliations:** 1 The Tomsk Polytechnic University, 634050 Tomsk, Russia; 2 Department of Chemistry and Biochemistry, University of Minnesota, Duluth MN 55812, USA; 3 School of Chemistry, Cardiff University, Park Place, Main Building, Cardiff CF10 3AT, UK

**Keywords:** diaryliodonium salts, iodine, iodonium, oxidation, Oxone

## Abstract

A facile synthesis of diaryliodonium salts utilizing Oxone as versatile and cheap oxidant has been developed. This method shows wide applicability and can be used for the preparation of iodonium salts containing electron-donating or electron-withdrawing groups in good yields. In addition, this procedure can be applied to the preparation of symmetric iodonium salts directly from arenes via a one-pot iodination–oxidation sequence.

## Introduction

Diaryliodonium salts, which are also known as diaryl-λ^3^-iodanes, are widely considered to be an important and practically useful class of hypervalent iodine compounds [1–4]. Diaryliodonium salts have found broad synthetic application as electrophilic arylating reagents in reactions with various nucleophiles including electron-rich carbon-centered species [5–7]. The unique arylating reactivity of diaryliodonium salts has been demonstrated in many metal-catalyzed and also metal-free transformations [8–15].

The development of novel synthetic approaches to diaryliodonium salts based on the use of inexpensive, commercially available oxidants is an important and challenging goal. A vast majority of existing procedures involve the interaction of electrophilic hypervalent iodine(III) species with suitable arenes through ligand exchange processes [16–20]. The reactive hypervalent iodine(III) species can be used as stable reagents or can be generated in situ [21–25]. In particular, Olofsson and co-workers reported procedures based on the in situ generation of reactive λ^3^-iodane species directly from arenes, which was a significant achievement in this field [26–29]. However, these now well-established processes involve oxidations using *m*CPBA in the presence of strong organic acids [30–35]. Therefore, the development of new, convenient and inexpensive methods utilizing readily available and easy-to-handle oxidants still remains a highly desirable goal.

Previously, we have published the utilization of Oxone^®^ (2KHSO_5_·KHSO_4_·K_2_SO_4_) as a readily available and effective oxidant for the preparation of various hypervalent iodine compounds [36–42]. Oxone is used as an efficient oxidant for the direct conversion of substituted 2-iodobenzoic acids to arylbenziodoxoles [37–38], 2-iodobiphenyl to dibenziodolium compounds [39], iodoarenes to iodylbenzenes and [bis(trifluoroacetoxy)iodo]arenes [40–41], and for the preparation of diaryliodonium trifluoroacetates and triflates [42]. Yakura also used Oxone^®^ as an oxidant for the generation of iodine(III) species in the oxidation of phenols [43]. In the present work, we report the development of a reliable and convenient procedure for the preparation of diaryliodonium bromides using Oxone in the presence of sulfuric acid.

## Results and Discussion

After having investigated previously described reaction conditions [37–38], initial optimization studies were performed using iodobenzene and toluene as reactants for the synthesis of diaryliodonium salt **3a** (Table 1). A simple mixing of the starting materials with finely ground Oxone and sulfuric acid leads to the formation of a very viscous and dark reaction mixture (Table 1, entry 1). Full conversion of the starting materials could not be achieved even after 24 h stirring. The addition of aqueous KBr to the reaction mixture resulted in the formation of the desired bromide salt **3a** in 30% isolated yield. The addition of KBr is necessary as the high solubility of the diaryliodonium sulfonates does not allow their isolation from the reaction mixture. Dilution of the reaction mixture with dichloromethane did not increase the yield of the target product significantly (Table 1, entry 2). Moreover, we observed the formation of 4-iodobenzenesulfonic acid in both cases, probably due to the high concentration of sulfuric acid in the reaction mixture. Mixing dichloromethane with acetonitrile resulted in an increased yield of **3a** and a decreased amount of 4-iodobenzenesulfonic acid (Table 1, entry 3). When the reaction was carried out in pure acetonitrile, the iodonium salt **3a** was formed in 75% yield (Table 1, entry 4). The addition of 2 equivalents of Oxone increased the yield to 92% (Table 1, entry 5). Surprisingly, a smaller amount of toluene did not affect the yield (Table 1, entry 6). In order to avoid the formation of undesired 4-iodobenzenesulfonic acid, the reaction was carried out using smaller amounts of acid (Table 1, entries 7 and 8). That reaction proceeds smoothly with only 7.5 equivalents of sulfuric acid producing the target compound **3a** high yields. However, the use of 3.75 equivalents of sulfuric acid resulted in a significantly lower yield.

**Table 1:** Optimization studies.^a^

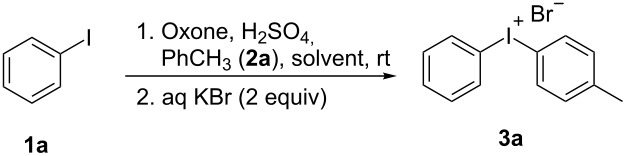					

entry	solvent	toluene (equiv)	Oxone (equiv)	H_2_SO_4_ (equiv)	yield^b^ (%)

1	–	2.8	1.3	15	30^c^
2	CH_2_Cl_2_	2.8	1.3	15	40^c^
3	CH_2_Cl_2_/MeCN	2.8	1.3	15	60^c^
4	MeCN	2.8	1.3	15	75^c^
5	MeCN	2.8	2	15	92^c^
6	MeCN	1.2	2	15	88^c^
7	MeCN	1.2	2	7.5	86
8	MeCN	1.2	2	3.75	53

^a^Reaction conditions: PhI (1 mmol), overnight, rt; ^b^isolated yield; ^c^according to NMR data product contains up to 10% of (4-tolyl)phenyliodonium 4-iodobenzenesulfonate as an impurity.

With the optimized procedure, the synthetic utility of this method using various aryliodides and arenes was investigated (Table 2). Iodobenzene (**1a**) smoothly reacts with arenes containing electron-donating substituents to form the corresponding iodonium salts in high yields. 3-Trifluoromethyliodobenzene (**1b**) exhibited higher reactivity, and iodonium salts have been isolated in higher yields. Moreover, iodoarene **1b** reacted with the moderately electron-poor chlorobenzene (**2e**) forming iodonium salt **3i** in 51% yield. In contrast, 4-bromoiodobenzene (**1c**) was less reactive and afforded iodonium salts **3j**–**m** in lower yields. Similar reactions of 3,5-bis(trifluoromethyl)iodobenzene (**1d**) with benzene and mesitylene formed the corresponding iodonium salts **3n** and **3o** in moderate yields.

**Table 2:** Synthesis of diaryliodonium bromides.^a^

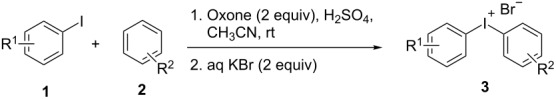			

R^1^ (**1**)	R^2^ (**2**)	product **3**	yield^b^ (%)

R^1^ = H (**1a**)	R^2^ = Me (**2a**)	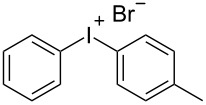 **3a**	86^c^
R^1^ = H (**1a**)	R^2^ = H (**2b**)	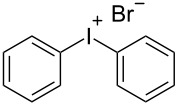 **3b**	74^d,e,f^
R^1^ = H (**1a**)	R^2^ = 1,4-Me_2_ (**2c**)	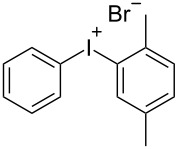 **3c**	75
R^1^ = H (**1a**)	R^2^ = 1,3,5-Me_3_ (**2d**)	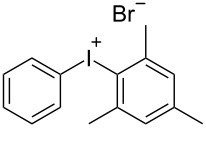 **3d**	75
R^1^ = 3-CF_3_ (**1b**)	R^2^ = H (**2b**)	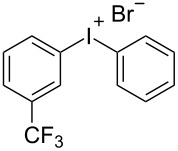 **3e**	85^e,f^
R^1^ = 3-CF_3_ (**1b**)	R^2^ = Me (**2a**)	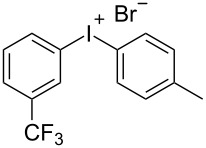 **3f**	76^c^
R^1^ = 3-CF_3_ (**1b**)	R^2^ = 1,4-Me_2_ (**2c**)	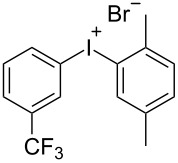 **3g**	95
R^1^ = 3-CF_3_ (**1b**)	R^2^ = 1,3,5-Me_3_ (**2d**)	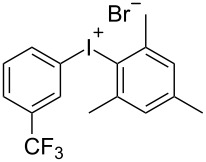 **3h**	92
R^1^ = 3-CF_3_ (**1b**)	R^2^ = Cl (**2e**)	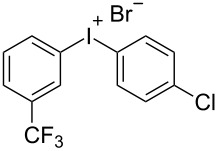 **3i**	51^g,h^
R^1^ = 4-Br (**1c**)	R^2^ = Me (**2a**)	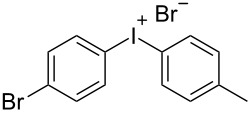 **3j**	70^c^
R^1^ = 4-Br (**1c**)	R^2^ = 1,4-Me_2_ (**2c**)	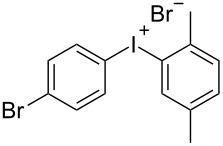 **3k**	32
R^1^ = 4-Br (**1c**)	R^2^ = 1,3,5-Me_3_ (**2d**)	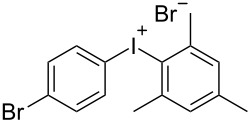 **3l**	71
R^1^ = 4-Br (**1c**)	R^2^ = Cl (**2e**)	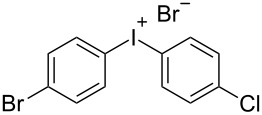 **3m**	34^g,h^
R^1^ = 3,5-(CF_3_)_2_ (**1d**)	R^2^ = H (**2b**)	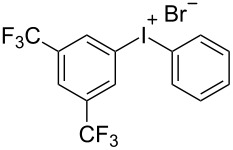 **3n**	35^e,f^
R^1^ = 3,5-(CF_3_)_2_ (**1d**)	R^2^ = 1,3,5-Me_3_ (**2d**)	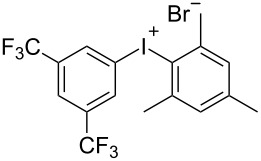 **3o**	62

^a^Reaction conditions: **1** (1 mmol), **2** (1.1 mmol), H_2_SO_4_ (7.5 mmol), MeCN (2 mL) overnight, rt; ^b^isolated yield; ^c^1.2 mmol of **2a** was used; ^d^according to NMR data product contains an up to 1.5% of diphenyliodonium 4-iodobenzenesulfonate as impurity; ^e^11.3 mmol of H_2_SO_4_ was used; ^f^1.3 mmol of **2b** was used; ^g^15 mmol of H_2_SO_4_ was used; ^h^1.5 mmol of **2e** was used.

With electron-deficient arenes **2** such as chlorobenzene (**2e**) and benzene, an excess of sulfuric acid and arene was used to improve the yields. Subsequently it was shown that the addition of aqueous potassium bromide can be modified and other counter anions can be introduced to prepare different diaryliodonium salts. This has been demonstrated in the preparation of diaryliodonium salts using 1-iodo-3-trifluoromethylbenzene (**1b**) and mesitylene (**2d**) as model substrates (Table 3).

**Table 3:** Preparation of diaryliodonium salts with different anions.^a^

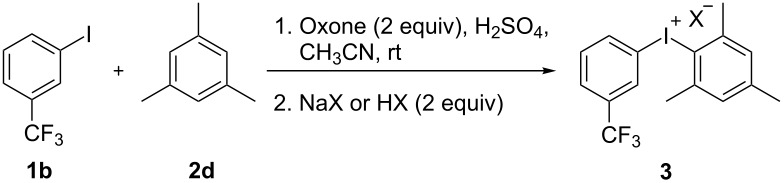		

NaX / HX	product	yield^b^ (%)

NaBr	**3h**	92
TsOH	**3p**	82
TfOH	**3q**	89
NaBF_4_	**3r**	81
NaPF_6_	**3s**	80

^a^Reaction conditions: **1b** (1 mmol), **2d** (1.1 mmol), Oxone (1 mmol), H_2_SO_4_ (7.5 mmol), MeCN (2 mL), NaX or HX (2 mmol) overnight, rt; ^b^isolated yield.

The yield of the salts **3h** and **3p**–**s** do not depend on the nature of the anion and its source. Small differences in yield can be explained by different solubility of salts in acetonitrile/water. Diaryliodonium bromides were isolated in higher yields because of the low solubility of these products (Table 3).

Finally, a one-step procedure for the preparation of symmetric iodonium salts directly from arenes via an in situ iodination was developed (Table 4). Arenes **2b**–**e** can be transformed to the symmetric iodonium salts **3b** and **3t**–**v** by the reaction with iodine, Oxone, and sulfuric acid. The attempted synthesis of the symmetric iodonium salt using toluene as substrate led to a regioisomeric mixture of products due to the low regioselectivity of iodination.

**Table 4:** Preparation of symmetric iodonium salts via a one-step iodination–oxidation procedure.^a^

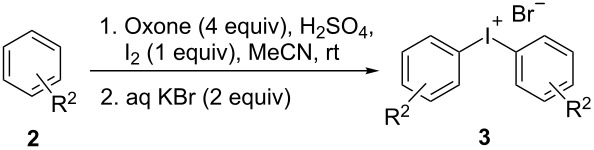		

R^2^ (**2**)	product **3**	yield^b^ (%)

R^2^ = H (**2b**)	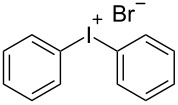 **3b**	84^c,d^
R^2^ = 1,4-Me_2_ (**2c**)	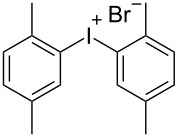 **3t**	80
R^2^ = 1,3,5-Me_3_ (**2d**)	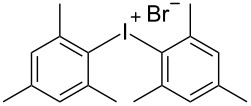 **3u**	77
R^2^ = Cl (**2e**)	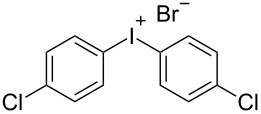 **3v**	40^e^

^a^Reaction conditions: ArH (2.2 mmol), Oxone (2 mmol), H_2_SO_4_ (7.5 mmol), MeCN (2 mL) overnight, rt; ^b^isolated yield; ^c^according to NMR data the product contains up to 1.5% of diphenyliodonium 4-iodobenzenesulfonate as impurity; ^d^11.3 mmol of H_2_SO_4_ and 2.6 mmol of **2b** were used; ^e^15 mmol of H_2_SO_4_ and 3.0 mmol of **2e** were used.

This procedure allowed the synthesis of iodonium salts with arenes containing electron-donating groups. Unfortunately, electron-poor arenes exhibited a lower reactivity and bis-(4-chlorophenyl)iodonium bromide have been isolated in only 40% yield. Nevertheless, the developed procedure is characterized by important advantages, such as simplicity, the use of inexpensive and available reagents, and typically good yields of iodonium salts. It is a versatile addition to the methodology toolbox for the preparation of diaryliodonium salts.

## Conclusion

In conclusion, a new facile protocol for the preparation of diaryliodonium salts using cheap and readily available Oxone as an oxidant in the presence of sulfuric acid has been developed. The procedure allows the synthesis of a wide range of iodonium salts containing electron-donating and electron-withdrawing substituents. Particularly attractive is the possibility of the one-pot synthesis of symmetric bis-aryliodonium salts directly from arenes via an iodination–oxidation sequence.

## Supporting Information

File 1 Experimental details and NMR spectra.
