# Supplementary material for: One-pot synthesis of diaryliodonium salts from arenes and aryl iodides with Oxone–sulfuric acid
Source: Beilstein J Org Chem. 2018 Apr 12;14:849–55. doi: 10.3762/bjoc.14.70 (PMC5905280; doi:10.3762/bjoc.14.70)
Supplement: File 1 — Experimental details and NMR spectra. [file Beilstein_J_Org_Chem-14-849-s001.pdf]

Supporting Information

for

One-pot synthesis of diaryliodonium salts from arenes and aryl iodides with Oxone–sulfuric acid

Natalia Soldatova[1], Pavel Postnikov[1], Olga Kukurina[1], Viktor V. Zhdankin[1,2], Akira Yoshimura[1], Thomas Wirth[3]* and Mekhman S. Yusubov[1]*

Address: [1]The Tomsk Polytechnic University, 634050 Tomsk, Russia; [2]Department of Chemistry and Biochemistry, University of Minnesota Duluth, MN 55812, USA, [3]School of Chemistry, Cardiff University Park Place, Main Building, Cardiff CF10 3AT, UK

Email: Thomas Wirth* - wirth@cf.ac.uk, Mekhman S. Yusubov* - yusubov@tpu.ru

* Corresponding author

Experimental details and NMR spectra

Contents:

Experimental.. S2
References S9
^{1}H, ^{13}C and ^{19}F NMR spectra S10

Experimental

General information

All aromatic precursors, and other reagents and solvents were from commercial sources and used without further purification from freshly opened containers. NMR spectra were recorded at 300 and 400 MHz (^{1}H NMR), 75, 100 MHz (^{13}C NMR), 376 MHz (^{19}F NMR). Chemical shifts (δ) are reported in parts per million. Mass spectrometric measurements were performed by the EPSRC Mass Spectrometry Service Centre, Swansea University or by R. Jenkins/R. Hick/S. Waller at Cardiff University. Ions were generated by the atmospheric pressure chemical ionization (APCI), Electrospray (ES) or Electron Ionization (EI).

General procedure for preparation of diaryliodonium bromides (3a–o).

Sulfuric acid (400–800 µL) was added to a stirred mixture of iodoarene (1 mmol), Oxone (1 mmol, 617 mg) and arene (1.1–3 mmol) in acetonitrile (2 mL). The reaction mixture was stirred overnight and a solution of KBr (2 mmol, 240 mg) in water (10 mL) was added. After the formation of a solid or oily residue, acetonitrile was removed under reduced pressure. To the residue Et_2O (10 mL) was added and the suspension stirred for 10 min. The precipitated diaryliodonium bromide was filtrated and washed with water (15 mL) and Et_2O (15 mL). The product was dried under vacuo.

Phenyl(*p*-tolyl)iodonium bromide (3a)

The reaction of iodobenzene (**1a**, 1 mmol, 204 mg), toluene (**2a**, 130 µL, 1.2 mmol), sulfuric acid (400 µL), KBr (2 mmol, 240 mg) according to the general procedure afforded 322 mg (86%) of phenyl(*p*-tolyl)iodonium bromide (**3a**) as a yellow crystalline solid; mp 179 – 180 ^{o}C (lit 178 – 179 ^{o}C [1]). ^{1}H NMR (300 MHz, DMSO-d_6): δ = 8.19 – 8.12 (m, 2H), 8.06 (d, J = 8.4 Hz, 2H), 7.58 (t, J = 7.5 Hz, 1H), 7.46 (t, J = 7.5 Hz, 2H), 7.27 (d, J = 8.1 Hz, 2H), 2.31 (s, 3H) ppm. ^{13}C NMR (75 MHz, DMSO-d_6): δ = 141.7, 134.9, 134.77, 132.0, 131.4, 131.3, 119.5, 115.8, 20.9 ppm. HRMS (NSI) m/z calcd for $C_{13}H_{12}I^+$: 294.9978; found: 294.9978.

Diphenyliodonium bromide (3b)

The reaction of iodobenzene (**1a**, 1 mmol, 204 mg), benzene (**2b**, 120 µL, 1.3 mmol), sulfuric acid (500 µL), KBr (2 mmol, 240mg) according to the general procedure afforded 268 mg (74%) of diphenyliodonium bromide (**3b**) as a yellow crystalline solid; mp 199 – 200 ^{o}C (lit 208 – 210 ^{o}C [2]). ^{1}H NMR (400 MHz, DMSO-d_6): δ = 8.19 (dd, J = 8.4, 1.2 Hz, 2H), 7.60 (t, J = 7.6 Hz, 1H), 7.47 (t, J = 7.6 Hz, 2H) ppm. ^{13}C NMR (75 MHz, DMSO- d_6:$CDCl_3$ 6:1): δ = 134.9, 131.3, 119.3 ppm. HRMS (NSI) m/z calcd for $C_{12}H_{10}I^+$: 280.9822; found: 280.9819.

(2,5-Dimethylphenyl)(phenyl)iodonium bromide (3c)

The reaction of iodobenzene (**1a**, 1 mmol, 204 mg), *p*-xylene (**2c**, 135 µL, 1.1 mmol), sulfuric acid (400 µL), KBr (2 mmol, 240 mg) according to the general procedure afforded 293 mg (75%) of (2,5-dimethylphenyl)(phenyl)iodonium bromide (**3c**) as a yellow crystalline solid; mp 159 – 160 ^oC. ^1H NMR (300 MHz, DMSO-d_6): δ = 8.20 (s, 1H), 8.13 (d, *J* = 7.8 Hz, 2H), 7.78 – 7.15 (m, 5H), 2.56 (s, 3H), 2.29 (s, 3H) ppm. ^{13}C NMR (75 MHz, DMSO-d_6:$CDCl_3$ 6:1): δ = 138.6, 136.9, 136.8, 134.4, 132.8, 131.3, 131.1, 130.7, 123.9, 119.1, 24.5, 20.1 ppm. HRMS (NSI) m/z calcd for $C_{14}H_{14}I^+$: 309.0135; found: 309.0132.

Mesityl(phenyl)iodonium bromide (3d)

The reaction of iodobenzene (**1a**, 1 mmol, 204 mg), mesitylene (**2d**, 150 µL, 1.1 mmol), sulfuric acid (400 µL), KBr (2 mmol, 240 mg) according to the general procedure afforded 301 mg (75%) of mesityl(phenyl)iodonium bromide (**3d**) as a white crystalline solid; mp 151 – 152 ^oC (lit 166 – 167 ^oC [3]). ^1H NMR (400 MHz, DMSO-d_6): δ = 7.88 (dd, *J* = 8.4, 1.2 Hz, 2H), 7.55 (t, *J* = 7.6 Hz, 1H), 7.43 (t, *J* = 8.0 Hz, 2H), 7.16 (s, 2H), 2.58 (s, 6H), 2.27 (s, 3H) ppm. ^{13}C NMR (75 MHz, DMSO- d_6:$CDCl_3$ 6:1): δ = 142.3, 140.9, 133.70, 131.4, 130.9, 129.5, 125.4, 118.2, 26.3, 20.6 ppm. HRMS (NSI) m/z calcd for $C_{15}H_{16}I^+$: 323.0291; found: 323.0287.

Phenyl(3-(trifluoromethyl)phenyl)iodonium bromide (3e)

The reaction of 3-iodo-1-(trifluoromethyl)benzene (**1b**, 1 mmol, 272 mg), benzene (**2b**, 120 µL, 1.3 mmol), sulfuric acid (500 µL), KBr (2 mmol, 240 mg) according to the general procedure afforded 365 mg (85%) of phenyl(3-(trifluoromethyl)phenyl)iodonium bromide **(3e)** as a white crystalline solid; mp 194 – 195 ^oC. ^1H NMR (300 MHz, DMSO-d_6): δ = 8.68 (s, 1H), 8.48 (d, *J* = 8.1 Hz, 1H), 8.25 (d, *J* = 7.5 Hz, 2H), 7.95 (d, *J* = 7.8 Hz, 1H), 7.68 (t, *J* = 7.8 Hz, 1H), 7.60 (t, *J* = 7.5 Hz, 1H), 7.48 (t, *J* = 7.8 Hz, 2H) ppm. ^{13}C NMR (75 MHz, DMSO-d_6): δ = 138.9, 135.0, 132.2, 131.5, 131.0 (q, J_{CF} = 33 Hz), 128.1, 124.9, 123.1 (q, *J* = 273 Hz, CF_3), 121.3, 120.8, 120.4 ppm. ^{19}F NMR (376 MHz, DMSO): δ = –61.16 ppm. HRMS (NSI) m/z calcd for $C_{13}H_9F_3I^+$: 348.9696; found: 348.9691.

p-Tolyl(3-(trifluoromethyl)phenyl)iodonium bromide (3f)

The reaction of 3-iodo-1-(trifluoromethyl)benzene (**1b**, 1 mmol, 272 mg), toluene (**2a**, 130 µL, 1.2 mmol), sulfuric acid (400 µL), KBr (2 mmol, 240 mg) according to the general procedure afforded 338 mg (76%) of p-tolyl(3-(trifluoromethyl)phenyl)iodonium bromide **(3f)** as a white crystalline solid; mp 178 – 179 ^oC. ^1H NMR (300 MHz, DMSO-d_6): δ = 8.66 (s, 1H), 8.44 (d, *J* = 8.1 Hz, 1H), 8.12 (d, *J* = 8.4 Hz, 2H), 7.95 (d, *J* = 7.8 Hz, 1H), 7.67 (t, *J* = 7.8 Hz, 1H), 7.29 (d, *J* = 8.1 Hz, 2H), 2.31 (s, 3H) ppm. ^{13}C NMR (75 MHz, DMSO-d_6): δ = 142.0, 138.8, 135.0, 132.1, 131.4, 130.9 (q, J_{CF} = 32 Hz), 128.0, 123.1 (q, J_{CF} = 273 Hz, CF_3), 120.8, 116.6, 20.9 ppm. ^{19}F

NMR (376 MHz, $CDCl_3$): δ = –62.65 ppm. HRMS (NSI) m/z calcd for $C_{14}H_{11}F_3I^+$: 362.9852; found: 362.9849.

(2,5-Dimethylphenyl)(3-(trifluoromethyl)phenyl)iodonium bromide (3g)

The reaction of 3-iodo-1-(trifluoromethyl)benzene (**1b**, 1 mmol, 272 mg), *p*-xylene (**2c**, 135 µL, 1.1 mmol), sulfuric acid (400 µL), KBr (2 mmol, 240 mg) according to the general procedure afforded 433 mg (95%) of (2,5-dimethylphenyl)(3-(trifluoromethyl)phenyl)iodonium bromide (**3g**) as a grey crystalline solid; mp 148 – 149 °C. ^1H NMR (400 MHz, DMSO-d_6): δ = 8.60 (s, 1H), 8.36 (d, J = 8.4 Hz, 1H), 8.26 (s, 1H), 7.95 (d, J = 8.0 Hz, 1H), 7.67 (t, J = 8.0 Hz, 1H), 7.39 (d, J = 7.6 Hz, 1H), 7.34 (d, J = 7.6 Hz, 1H), 2.58 (s, 3H), 2.28 (s, 3H) ppm. ^{13}C NMR (75 MHz, DMSO- d_6:$CDCl_3$ 6:1): δ = 138.7, 138.3, 137.0, 136.8, 133.0, 132.2, 131.1, 131.14 (q, J_{CF} = 33 Hz), 130.8, 127.8, 124.2, 123.0 (q, J_{CF}=273 Hz, CF_3), 120.1, 24.5, 20.1 ppm. ^{19}F NMR (376 MHz, DMSO-d_6): δ = –61.15 ppm. HRMS (ESI) m/z calcd for $C_{15}H_{13}F_3I^+$: 377.0014; found: 377.0001.

Mesityl(3-(trifluoromethyl)phenyl)iodonium bromide (3h)

The reaction of 3-iodo-1-(trifluoromethyl)benzene (**1b**, 1 mmol, 272 mg), mesitylene (**2d**, 150 µL, 1.1 mmol), sulfuric acid (400 µL), KBr (2 mmol, 240 mg) according to the general procedure afforded 433 mg (92%) of mesityl(3-(trifluoromethyl)phenyl)iodonium bromide (**3h**) as a white crystalline solid; mp 138 – 139 °C. ^1H NMR (400 MHz, DMSO-d_6): δ = 8.36 (s, 1H), 7.98 (d, J = 7.6 Hz, 1H), 7.91 (d, J = 7.6 Hz, 1H), 7.63 (t, J = 8.0 Hz, 1H), 7.17 (s, 2H), 2.59 (s, 6H), 2.27 (s, 3H) ppm. ^{13}C NMR (100 MHz, DMSO): δ = 142.5, 141.0, 137.3, 132.4, 131.0 (q, J_{CF} = 33 Hz), 130.3, 129.6, 127.7, 126.0, 123.1 (q, J_{CF} = 273 Hz, CF_3), 119.7, 26.3, 20.5 ppm. ^{19}F NMR (376 MHz, DMSO-d_6): δ = –61.28 ppm. HRMS (NSI) m/z calcd for $C_{16}H_{15}F_3I^+$: 391.0165; found: 391.0165.

(4-Chlorophenyl)(3-(trifluoromethyl)phenyl)iodonium bromide (3i)

The reaction of 3-iodo-1-(trifluoromethyl)benzene (**1b**, 1 mmol, 272 mg), chlorobenzene (**2e**, 150 µL, 1.5 mmol), sulfuric acid (800 µL), KBr (2 mmol, 240 mg) according to the general procedure afforded 236 mg (51%) of (4-chlorophenyl)(3-(trifluoromethyl)phenyl)iodonium bromide (**3i**) as a white crystalline solid; mp 175 – 176 °C. ^1H NMR (300 MHz, DMSO-d_6): δ = 8.71 (s, 1H), 8.49 (d, J = 7.8 Hz, 1H), 8.26 (d, J = 8.7 Hz, 2H), 7.97 (d, J = 7.2 Hz, 1H), 7.69 (t, J = 8.1 Hz, 1H), 7.58 (d, J = 8.7 Hz, 2H) ppm. ^{13}C NMR (75 MHz, DMSO-d_6): δ = 138.9, 136.8, 136.7, 132.2, 131.5, 131.4, 131.0 (q, J_{CF} = 33 Hz), 128.0, 123.1 (q, J_{CF} = 273 Hz, CF_3), 121.7, 119.2 ppm. ^{19}F NMR (376 MHz, DMSO-d_6): δ = –61.14 ppm. HRMS (NSI) m/z calcd for $C_{13}H_8ClF_3I^+$: 382.9306; found: 382.9305.

(4-Bromophenyl)(*p*-tolyl)iodonium bromide (3j)

The reaction of 1-bromo-4-iodobenzene (**1c**, 1 mmol, 282 mg), toluene (**2a**, 130 µL, 1.2 mmol), sulfuric acid (400 µL), KBr (2 mmol, 240 mg) according to the general procedure afforded 316 mg (70%) of (4-bromophenyl)(*p*-tolyl)iodonium bromide (**3j**) as a white crystalline solid; mp 171 – 172 ^{o}C. ^{1}H NMR (400 MHz, DMSO-d_6): δ = 8.09 (d, J = 6.4 Hz, 2H), 8.07 (d, J = 6.4 Hz, 2H), 7.68 (d, J = 8.4 Hz, 2H), 7.28 (d, J = 8.4 Hz, 2H), 2.32 (s, 3H) ppm. ^{13}C NMR (75 MHz, DMSO-d_6): δ = 141.8, 136.7, 134.9, 134.2, 132.1, 125.3, 118.7, 116.3, 20.8 ppm. HRMS (NSI) m/z calcd for $C_{13}H_{11}BrI^+$: 372.9083; found: 372.9085.

(4-Bromophenyl)(2,5-dimethylphenyl)iodonium bromide (3k)

The reaction of 1-bromo-4-iodobenzene (**1c**, 1 mmol, 282 mg), *p*-xylene (**2c**, 135 µL, 1.1 mmol), sulfuric acid (400 µL), KBr (2 mmol, 240 mg) according to the general procedure afforded 149 mg (32%) of (4-bromophenyl)(2,5-dimethylphenyl)iodonium bromide (**3k**) as a white crystalline solid; mp 180 – 181 ^{o}C. ^{1}H NMR (400 MHz, DMSO-d_6): δ = 8.20 (s, 1H), 8.05 (d, J = 8.8 Hz, 2H), 7.68 (d, J = 8.4 Hz, 2H), 7.40 (d, J = 8.0 Hz, 1H), 7.35 (d, J = 8.0 Hz, 1H), 2.55 (s, 3H), 2.29 (s, 3H) ppm. ^{13}C NMR (75 MHz, DMSO-d_6:$CDCl_3$ 6:1): δ = 138.9, 137.1, 136.9, 136.6, 134.4, 133.2, 130.9, 125.6, 118.6, 116.8, 24.5, 20.1 ppm. HRMS (NSI) m/z calcd for $C_{14}H_{13}BrI^+$: 386.9240; found: 386.9240.

(4-Bromophenyl)(mesityl)iodonium bromide (3l)

The reaction of 1-bromo-4-iodobenzene (**1c**, 1 mmol, 282 mg), mesitylene (**2d**, 150 µL, 1.1 mmol), sulfuric acid (400 µL), KBr (2 mmol, 240 mg) according to the general procedure afforded 316 mg (71%) of (4-bromophenyl)(mesityl)iodonium bromide (**3l**) as a white crystalline solid; mp 154 – 156 ^{o}C. ^{1}H NMR (400 MHz, DMSO-d_6): δ = 7.82 (d, J = 8.8 Hz, 2H), 7.66 (d, J = 8.8 Hz, 2H), 7.18 (s, 2H), 2.58 (s, 6H), 2.28 (s, 3H) ppm. ^{13}C NMR (100 MHz, DMSO-d_6): δ = 142.5, 141.0, 135.8, 134.3, 129.6, 125.4, 125.0, 116.9, 26.3, 20.6 ppm. HRMS (NSI) m/z calcd for $C_{15}H_{15}BrI^+$: 400.9396; found: 400.9395.

(4-Bromophenyl)(4-chlorophenyll)iodonium bromide (3m)

The reaction of 1-bromo-4-iodobenzene (**1c**, 1 mmol, 282 mg), chlorobenzene (**2e**, 150 µL, 1.5 mmol), sulfuric acid (800 µL), KBr (2 mmol, 240 mg) according to the general procedure afforded 161 mg (34%) of (4-bromophenyl)(4-chlorophenyl)iodonium bromide (**3m**) as a white crystalline solid; mp 169 – 170 ^{o}C. ^{1}H NMR (300 MHz, DMSO-d_6): δ = 8.23 (d, J = 8.4 Hz, 2H), 8.15 (d, J = 8.1 Hz, 2H), 7.73 (d, J = 8.4 Hz, 2H), 7.60 (d, J = 8.4 Hz, 2H) ppm. ^{13}C NMR (75 MHz, DMSO-d_6:$CDCl_3$ 6:1): δ = 137.0, 136.9, 136.8, 134.3, 131.5, 125.8, 117.5, 116.8 ppm. HRMS (NSI) m/z calcd for $C_{12}H_8ClBrI^+$: 392.8537; found: 392.8533.

(3,5-Bis(trifluoromethyl)phenyl)(phenyl)iodonium bromide (3n)

The reaction of 1-iodo-3,5-bis(trifluoromethyl)benzene (**1d**, 1 mmol, 340 mg), benzene (**2b**, 120 µL, 1.3 mmol), sulfuric acid (500 µL), KBr (2 mmol, 240 mg) according to the general procedure afforded 174 mg (35%) of (3,5-bis(trifluoromethyl)phenyl)(phenyl)iodonium bromide **3n** as a white crystalline solid; mp 190 – 192 ^{o}C. ^{1}H NMR (400 MHz, DMSO-d_6): δ = 9.01 (s, 2H), 8.37 (s, 1H), 8.30 (dd, J = 8.4, 1.2 Hz, 1H), 7.62 (t, J = 7.2 Hz, 1H), 7.51 (t, J = 7.6 Hz, 2H) ppm. ^{13}C NMR (75 MHz, DMSO-d_6-$CDCl_3$ 6:1): δ = 135. 6, 135.1, 131.8 (q, J_{CF} = 34 Hz), 131.6, 131.4, 131.13, 125.0, 122.5, 122.7 (q, J = 274 Hz, CF_3), 120.7 ppm. ^{19}F NMR (376 MHz, DMSO-d_6): δ = –61.21 ppm. HRMS (ESI) m/z calcd for $C_{14}H_8F_6I^+$: 416.9575; found: 416.9584.

(3,5-Bis(trifluoromethyl)phenyl)(mesityl)iodonium bromide (3o)

The reaction of 1-iodo-3,5-bis(trifluoromethyl)benzene (**1d**, 1 mmol, 340 mg), mesitylene (**2d**, 150 µL, 1.1 mmol), sulfuric acid (400 µL), KBr (2 mmol, 240 mg) according to the general procedure afforded 334 mg (62%) of (3,5-bis(trifluoromethyl)phenyl)(mesityl)iodonium bromide (**3o**) as a white crystalline solid; mp 142 – 144 ^{o}C. ^{1}H NMR (400 MHz, DMSO-d_6): δ = 8.43 (s, 2H), 8.34 (s, 1H), 7.21 (s, 2H), 2.61 (s, 6H), 2.29 (s, 3H) ppm. ^{13}C NMR (75 MHz, DMSO-d_6-$CDCl_3$ 6:1): δ = 142.9, 141.1, 133.6, 132.0 (q, J_{CF} = 34 Hz), 129.7, 125.6, 124.7, 122.2 (q, J = 274 Hz, CF_3), 121.3, 26.31, 20.57 ppm. ^{19}F NMR (376 MHz, DMSO-d_6): δ = –61.36 ppm. HRMS (ESI) m/z calcd for $C_{17}H_{14}F_6I^+$: 459.0044; found: 459.0060.

General procedure for preparation of symmetric diaryliodonium bromides (3b, 3t–v).

Sulfuric acid (400–800 µL) was added to a stirred mixture of iodine (0.5 mmol, 127 mg), Oxone (2 mmol, 1.23 g) and arene (2.2–3 mmol) in acetonitrile (2 mL). The reaction mixture was stirred overnight and a solution of KBr (2 mmol, 240 mg) in water (10 mL) was added. After the formation of solid or oil residue acetonitrile was removed under low pressure. To the residue Et_2O (10 mL) was added and the suspension was stirred for 10 min. The precipitated diaryliodonium bromide was filtrated and washed with water (15 mL) and Et_2O (15 mL). The product was dried under vacuo.

Diphenyliodonium bromide (3b)

The reaction of iodine (0.5 mmol, 127 mg), benzene (**2b**, 240 µL, 2.6 mmol), sulfuric acid (500 µL), KBr (2 mmol, 240mg) according to the general procedure afforded 304 mg (84%) of diphenyliodonium bromide (**3b**) as a yellow crystalline solid; mp 201 – 202 ^{o}C (lit 208 – 210 ^{o}C [2]).

Bis(2,5-dimethylphenyl)iodonium bromide (3t)

The reaction of iodine (0.5 mmol, 127 mg), *p*-xylene (**2c**, 270 µL, 2.2 mmol), sulfuric acid (400 µL), KBr (2 mmol, 240 mg) according to the general procedure afforded 334 mg (80%) of **3t** as a white crystalline solid; mp 129 – 130 ^{o}C. ^{1}H NMR (300 MHz, DMSO-d_6): δ = 8.10 (s, 2H),

7.40 (d, J = 7.8 Hz, 2H), 7.34 (d, J = 7.8 Hz, 2H), 2.56 (s, 6H), 2.29 (s, 6H) ppm. ^{13}C NMR (75 MHz, DMSO-d_6): δ = 138.8, 137.2, 136.9, 133.0, 131.0, 121.9, 24.5, 20.1 ppm. HRMS (NSI) m/z calcd for $C_{16}H_{18}I^+$: 337.0448; found: 337.0446.

Dimesityliodonium bromide (3u)

The reaction of iodine (0.5 mmol, 127 mg), mesitylene (**2d**, 300 µL, 2.2 mmol), sulfuric acid (400 µL), KBr (2 mmol, 240 mg) according to the general procedure afforded 344 mg (77%) of **3u** as a white crystalline solid; mp 114 – 115 ^{o}C (lit 134-135 ^{o}C [4]). ^{1}H NMR (300 MHz, MeOD): δ = 7.16 (s, 4H), 2.51 (s, 12H), 2.32 (s, 6H) ppm. ^{13}C NMR (75 MHz, MeOD): δ = 145.2, 143.6, 131.7, 119.5, 31.5, 20.9 ppm. HRMS (NSI) m/z calcd for $C_{18}H_{22}I^+$: 365.0761; found: 365.0755.

Bis(4-chlorophenyl)iodonium bromide (3v)

The reaction of iodine (0.5 mmol, 127 mg), chlorobenzene (**2e**, 300 µL, 3.0 mmol), sulfuric acid (800 µL), KBr (2 mmol, 240 mg) according to the general procedure afforded 171 mg (40%) of **3v** as a white crystalline solid; mp 188 – 189 ^{o}C (lit 189 – 190 ^{o}C [5]). ^{1}H NMR (300 MHz, DMSO-d_6): δ = 8.20 (d, J = 8.7 Hz, 1H), 7.55 (d, J = 8.7 Hz, 1H) ppm. ^{13}C NMR (75 MHz, DMSO-d_6): δ = 136.7, 136.7, 131.4, 118.4 ppm. HRMS (NSI) m/z calcd for $C_{12}H_8Cl_2I^+$: 348.9042; found: 348.9043.

Procedure for preparation of mesityl(3-(trifluoromethyl)phenyl)iodonium tosylate (3p).

Sulfuric acid (400 µL) was added to a stirred mixture of 3-iodo-1-(trifluoromethyl)benzene (**1b**, 1 mmol, 272 mg), Oxone (1 mmol, 617 mg) and mesitylene (**2d**, 150 µL, 1.1 mmol) in acetonitrile (2 mL). The reaction mixture was stirred overnight and a solution of TsOH·H_2O (2 mmol, 380 mg) in water (10 mL) was added. After the formation of a solid residue, acetonitrile was removed under reduced pressure. To the residue Et_2O (10 mL) was added and the suspension was stirred for 10 min. The precipitated diaryliodonium tosylate **3p** was filtrated and washed with water (15 mL) and Et_2O (15 mL). The product was dried under vacuo. The product was obtained in 82% yield (460 mg) as a white crystalline solid; mp 162 – 163 ^{o}C. ^{1}H NMR (400 MHz, DMSO-d_6): δ = 8.48 (s, 1H), 8.11 (d, J = 8.4 Hz, 1H), 8.01 (d, J = 7.6 Hz, 1H), 7.70 (t, J = 8.0 Hz, 1H), 7.45 (d, J = 8.0 Hz, 2H), 7.23 (s, 2H), 7.10 (d, J = 8.0 Hz, 2H), 2.59 (s, 6H), 2.30 (s, 3H), 2.28 (s, 3H) ppm. ^{13}C NMR (75 MHz, DMSO-d_6:$CDCl_3$ 6:1): δ = 145.3, 143.3, 141.7, 137.8, 132.8, 131.4 (q, J_{CF} = 33 Hz), 130.9, 129.8, 128.3, 128.0, 125.5, 122.9 (q, J_{CF} = 273 Hz, CF_3), 122.6, 114.7, 26.4, 20.8, 20.6 ppm. ^{19}F NMR (376 MHz, DMSO-d_6): δ = –61.26 ppm. HRMS (ESI) m/z calcd for $C_{16}H_{15}F_3I^+$: 391.0171; found: 391.0170.

General procedure for preparation of mesityl(3-(trifluoromethyl)phenyl)iodonium salts (3q–s).

Sulfuric acid (400 µL) was added to a stirred mixture of 3-iodo-1-(trifluoromethyl)benzene (**1b**, 1 mmol, 272 mg), Oxone (1mmol, 617 mg) and mesitylene (**2d**, 150 µL, 1.1 mmol) in acetonitrile (2 mL). The reaction mixture was stirred overnight and a solution of salt or acid (2 mmol) in water (10 mL) was added. The diaryliodonium salt was extracted with CH_2Cl_2 (3 × 5 mL). The combined organic layers were dried with Na_2SO_4 and the solvent was removed under the reduced pressure. A mixture of hexane (9 mL) and Et_2O (1 mL) was added to the residue. The product was filtered off and washed with hexane (3 × 5 mL). The product was dried under vacuo.

Mesityl(3-(trifluoromethyl)phenyl)iodonium trifluoromethanesulfonate (3q)

The reaction of 3-iodo-1-(trifluoromethyl)benzene (**1b**, 1 mmol, 272 mg), mesitylene (**2d**, 150 µL, 1.1 mmol), sulfuric acid (400 µL), TfOH (2 mmol, 176 µL) according to the general procedure afforded 481 mg (89%) of **3q** as a beige crystalline solid; mp 100 – 101 ^{o}C (lit 107 – 108 ^{o}C [6]). ^{1}H NMR (400 MHz, $CDCl_3$): δ = 7.94 (d, *J* = 8.0 Hz, 1H), 7.91 (s, 1H), 7.77 (d, *J* = 8.0 Hz, 1H), 7.54 (t, *J* = 8.0 Hz, 1H), 7.12 (s, 2H), 2.63 (s, 6H), 2.37 (s, 3H) ppm. ^{13}C NMR (100 MHz, $CDCl_3$): δ = 144.9, 142.6, 136.5, 134.2 (q, J_{CF} = 34 Hz), 132.5, 130.6, 129.8, 128.6, 122.5 (q, J_{CF} = 272 Hz, CF_3), 120.2 (q, J_{CF} = 318Hz, $CF_3SO_3^-$), 120.9, 112.0, 27.3, 21.3 ppm. ^{19}F NMR (376 MHz, $CDCl_3$): δ = –63.01, –78.54 ppm. HRMS (ESI) m/z calcd for $C_{16}H_{15}F_3I^+$: 391.0171; found: 391.0189.

Mesityl(3-(trifluoromethyl)phenyl)iodonium tetrafluoroborate (3r)

The reaction of 3-iodo-1-(trifluoromethyl)benzene (**1b**, 1 mmol, 272 mg), mesitylene (**1d**, 150 µL, 1.1 mmol), sulfuric acid (400 µL), $NaBF_4$ (2 mmol, 220 mg) according to the general procedure afforded 387 mg (81%) of **3r** as a beige crystalline solid; mp 133 – 134 ^{o}C. ^{1}H NMR (400 MHz, DMSO-*d*$_6$): δ = 8.48 (s, 1H), 8.11 (d, *J* = 8.0 Hz, 1H), 8.02 (d, *J* = 8.0 Hz, 1H), 7.70 (t, *J* = 8.0 Hz, 1H), 7.24 (s, 2H), 2.60 (s, 6H), 2.31 (s, 3H) ppm. ^{13}C NMR (75 MHz, $CDCl_3$): δ = 144.7, 142.8, 136.9, 134.3 (q, J_{CF} = 34 Hz), 132.6, 130.5, 129.3, 128.4, 122.6 (q, J_{CF} = 272 Hz, CF_3), 120.5, 112.1, 27.2, 21.3 ppm. ^{19}F NMR (376 MHz, DMSO-d$_6$): δ = –61.25, –148.22 ppm. HRMS (ESI) m/z calcd for $C_{16}H_{15}F_3I^+$: 391.0171; found: 391.0164.

Mesityl(3-(trifluoromethyl)phenyl)iodonium hexafluorophosphate (3s)

The reaction of 3-iodobenzotrifluoride (**1b**, 1 mmol, 272 mg), mesitylene (**2d**, 150 µL, 1.1 mmol), sulfuric acid (400 µL), $NaPF_6$ (2 mmol, 336 mg) according to the general procedure afforded 329 mg (80%) of) **3s** as a beige crystalline solid; mp 165 – 166 ^{o}C. ^{1}H NMR (400 MHz, DMSO-*d*$_6$): δ = 8.49 (s, 1H), 8.12 (d, *J* = 8.0 Hz, 1H), 8.02 (d, *J* = 8.4 Hz, 1H), 7.70 (t, *J* = 8.0 Hz, 1H), 7.24 (s, 2H), 2.60 (s, 6H), 2.31 (s, 3H) ppm. ^{13}C NMR (75 MHz, DMSO-*d*$_6$:$CDCl_3$ 6:1):

δ = 143.5, 141.7, 137.8, 132.8, 131.5 (q, J_{CF} = 33 Hz), 130.9, 129.9, 128.4, 122.9 (q, J_{CF} = 272 Hz, CF_3), 122.5, 114.6, 26.4, 20.6 ppm. ^{19}F NMR (376 MHz, DMSO-d_6): δ = –61.27, –70.11 (d, J_{PF} = 710 Hz) ppm. HRMS (ESI) m/z calcd for $C_{16}H_{15}F_3I^+$: 391.0171; found: 391.0171.

References

1. Kamierczak P., Skulski L. *Synthesis* **1995**, 1027-10329.
2. McKerrow J. D., Al-Rawi J. M. A., Brooks P. *Synth. Commun.* **2010**, *40*, 1161-1179.
3. Kryska A., Skulski L. *Molecules* **2001**, *6*, 875-880.
4. Miralles, N., Romero, R. M., Fernández, E., Muñiz, K. *Chem. Commun.* **2015**, *51*, 14068-14071.
5. Beringer F. M., Dehn Jr J. W., Winicov M. *J. Am. Chem. Soc.* **1960**, *82*, 2948-2952.
6. Hossain, M. D., Kitamura, T. *Tetrahedron* **2006**, *62*, 6955-696.

^{1}H, ^{13}C and ^{19}F NMR spectra

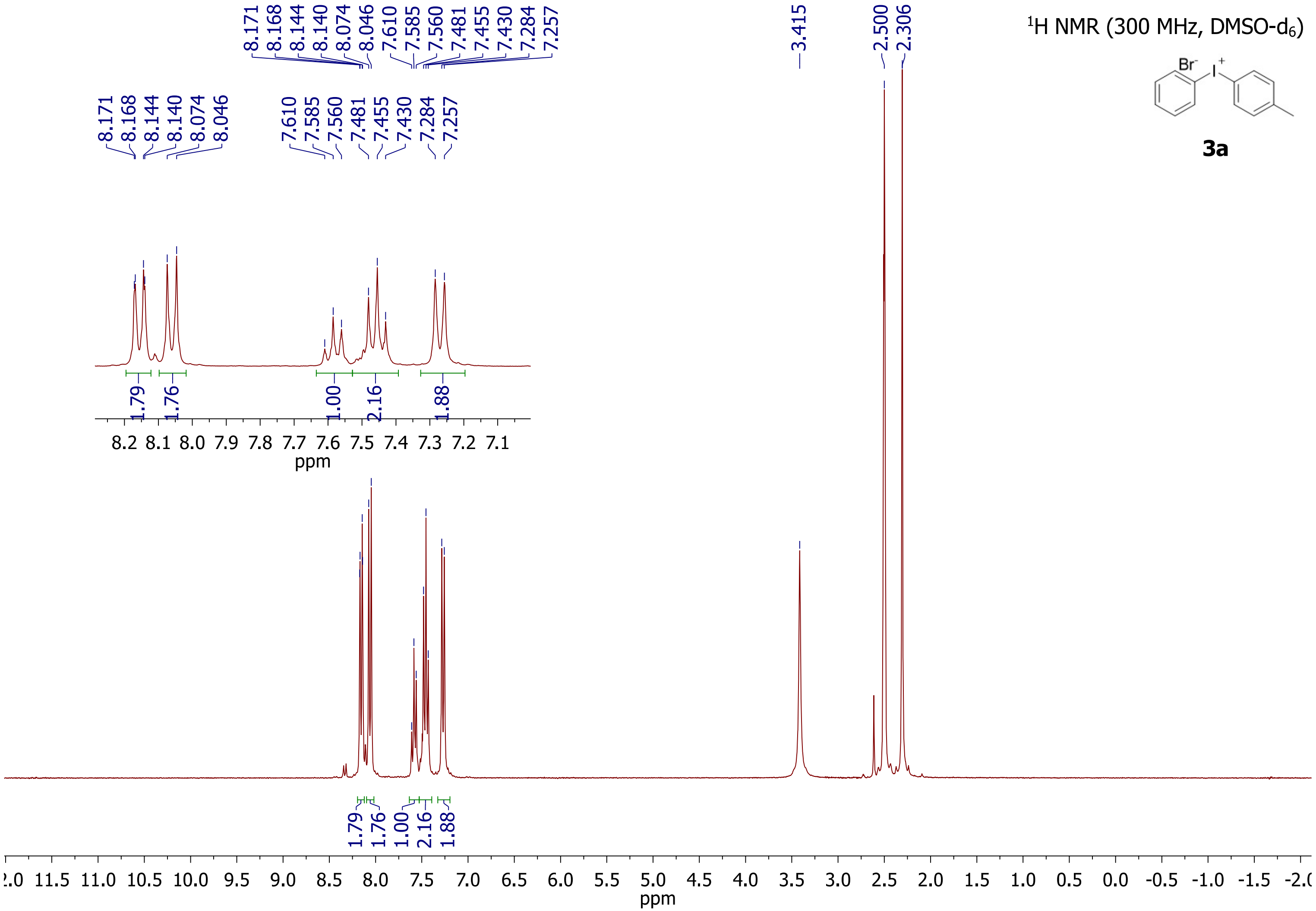

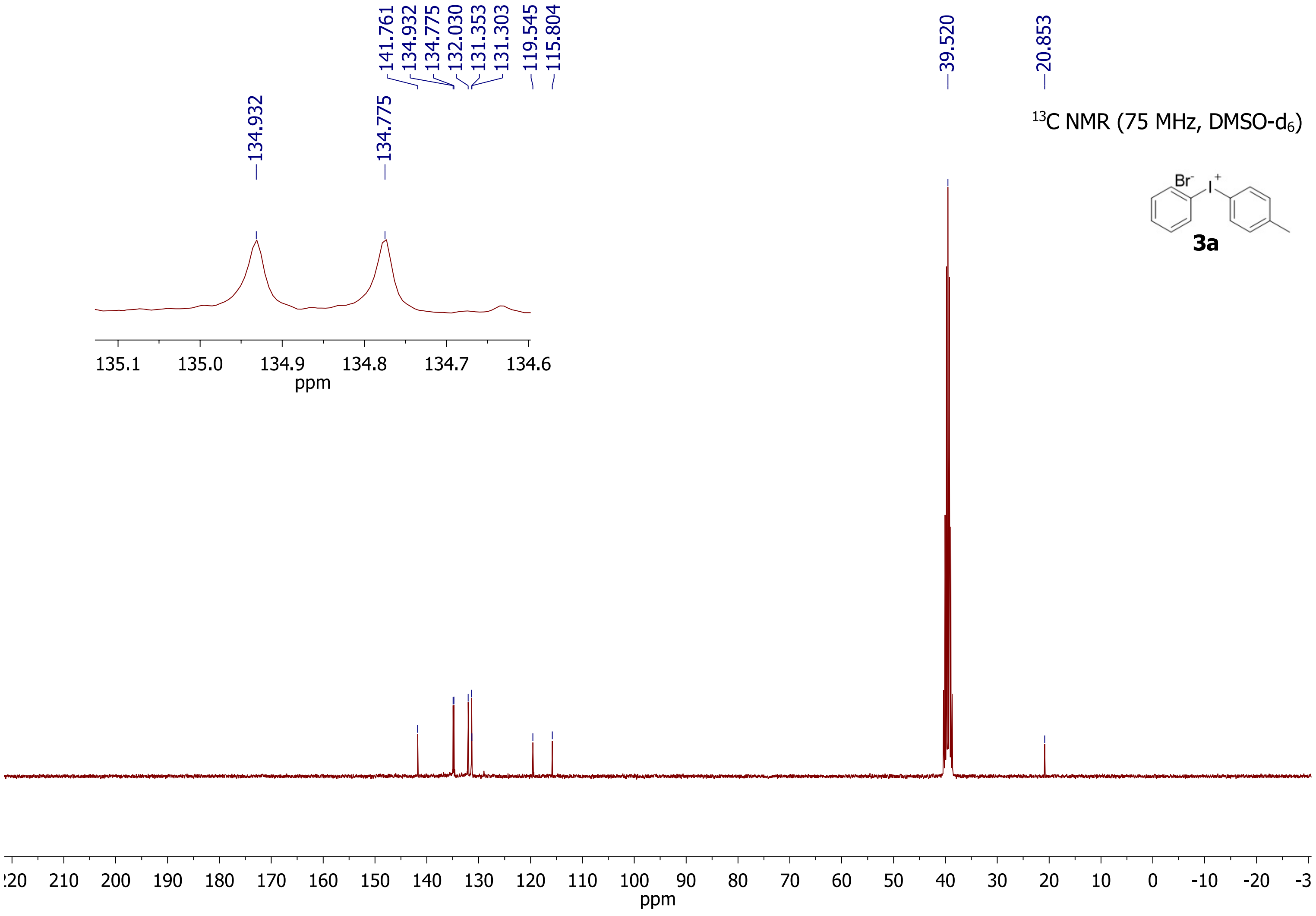
141.761
134.932
134.775
132.030
131.353
131.303
119.545
115.804
39.520
20.853
134.932
134.775
135.1
135.0
134.9
134.8
134.7
134.6
ppm
13C NMR (75 MHz, DMSO-d6)
Br-
I+
3a
220
210
200
190
180
170
160
150
140
130
120
110
100
90
80
70
60
50
40
30
20
10
0
-10
-20
-30
ppm

^{1}H NMR (400 MHz, DMSO-d_6)

3b

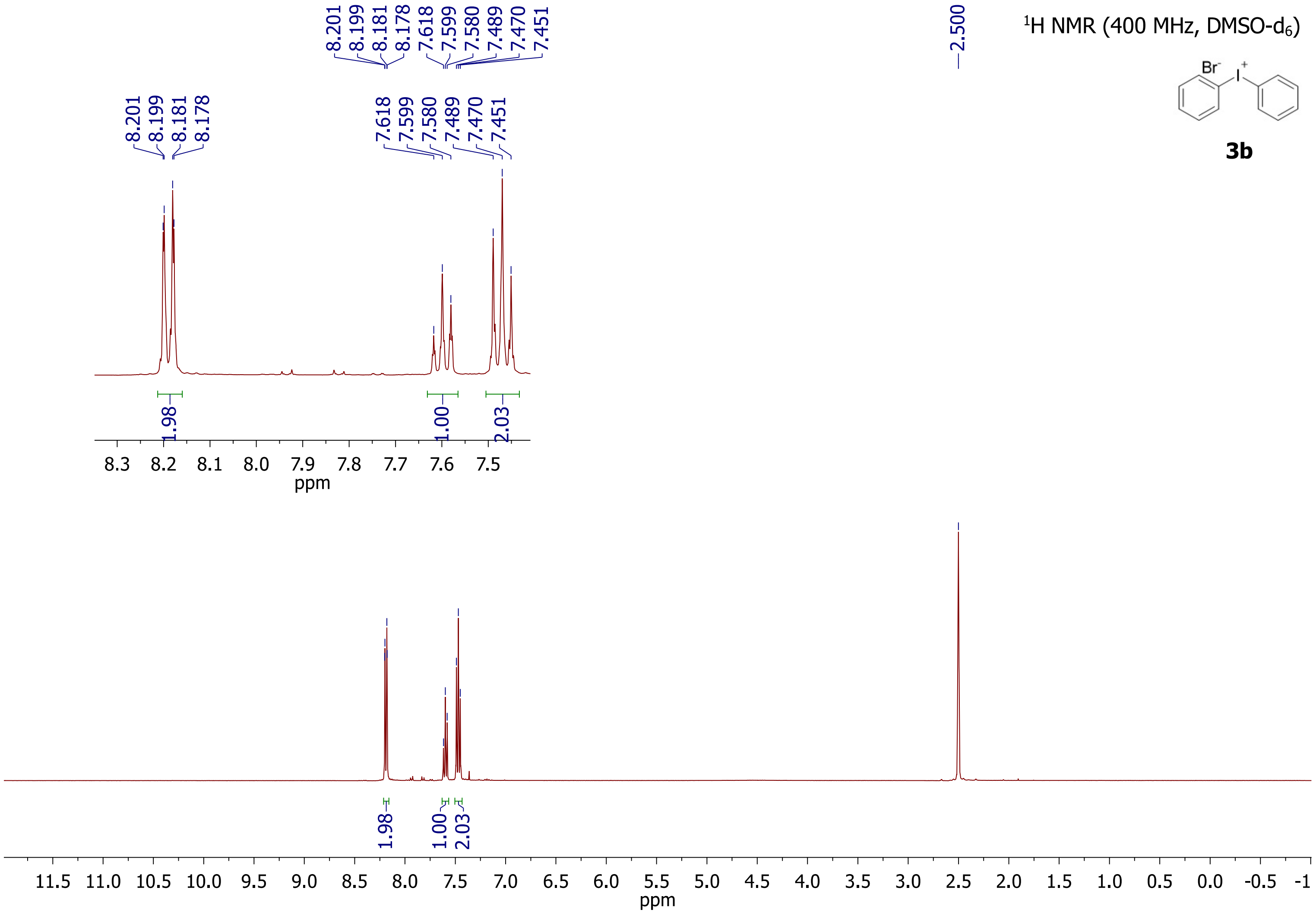

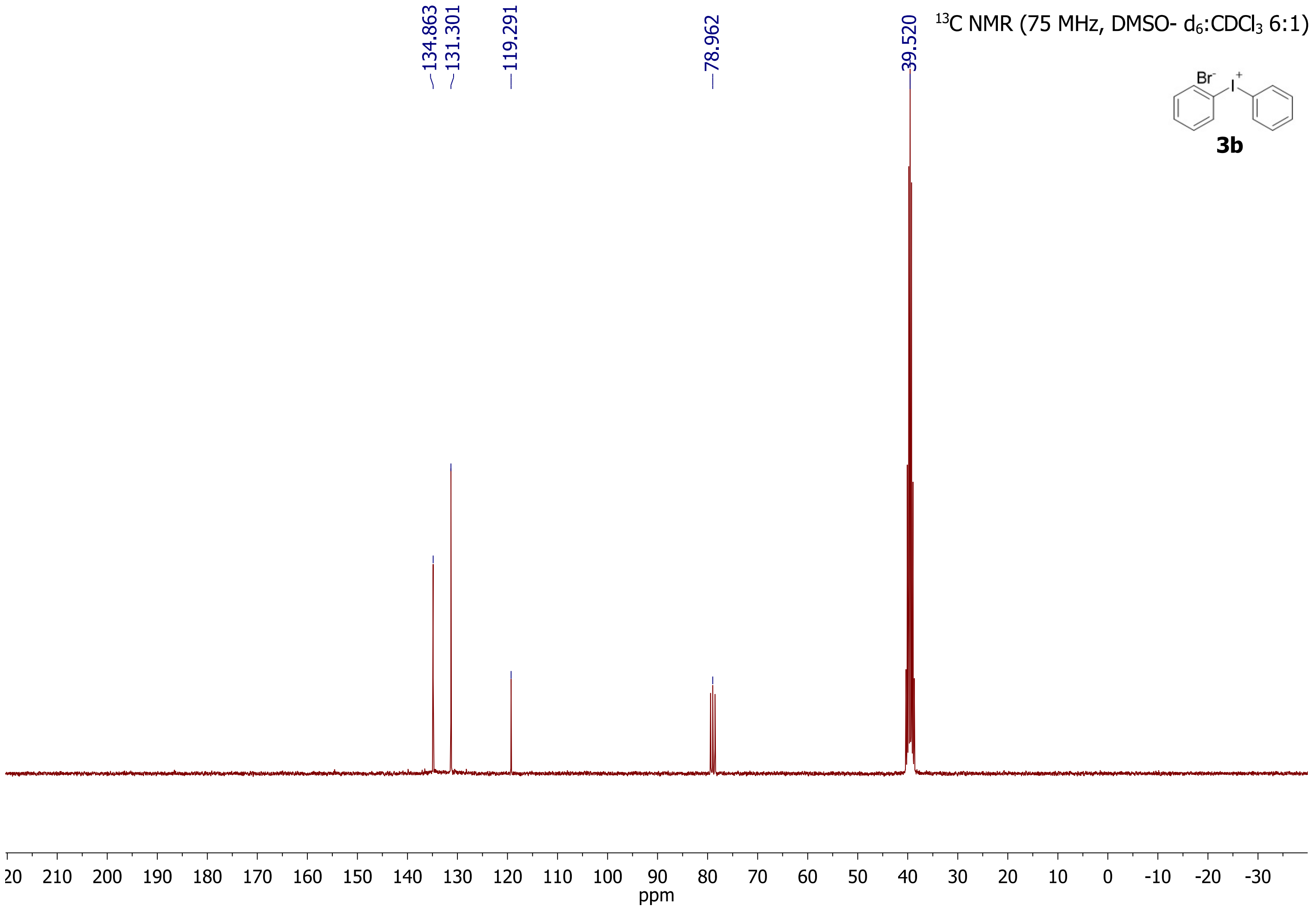

134.863
131.301
119.291
78.962
39.520
^{13}C NMR (75 MHz, DMSO- d_6:$CDCl_3$ 6:1)
Br⁻
I⁺
3b
20
210
200
190
180
170
160
150
140
130
120
110
100
90
80
70
60
50
40
30
20
10
0
-10
-20
-30
ppm

^{1}H NMR (300 MHz, DMSO-d_6)

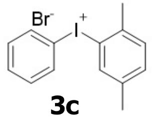

3c

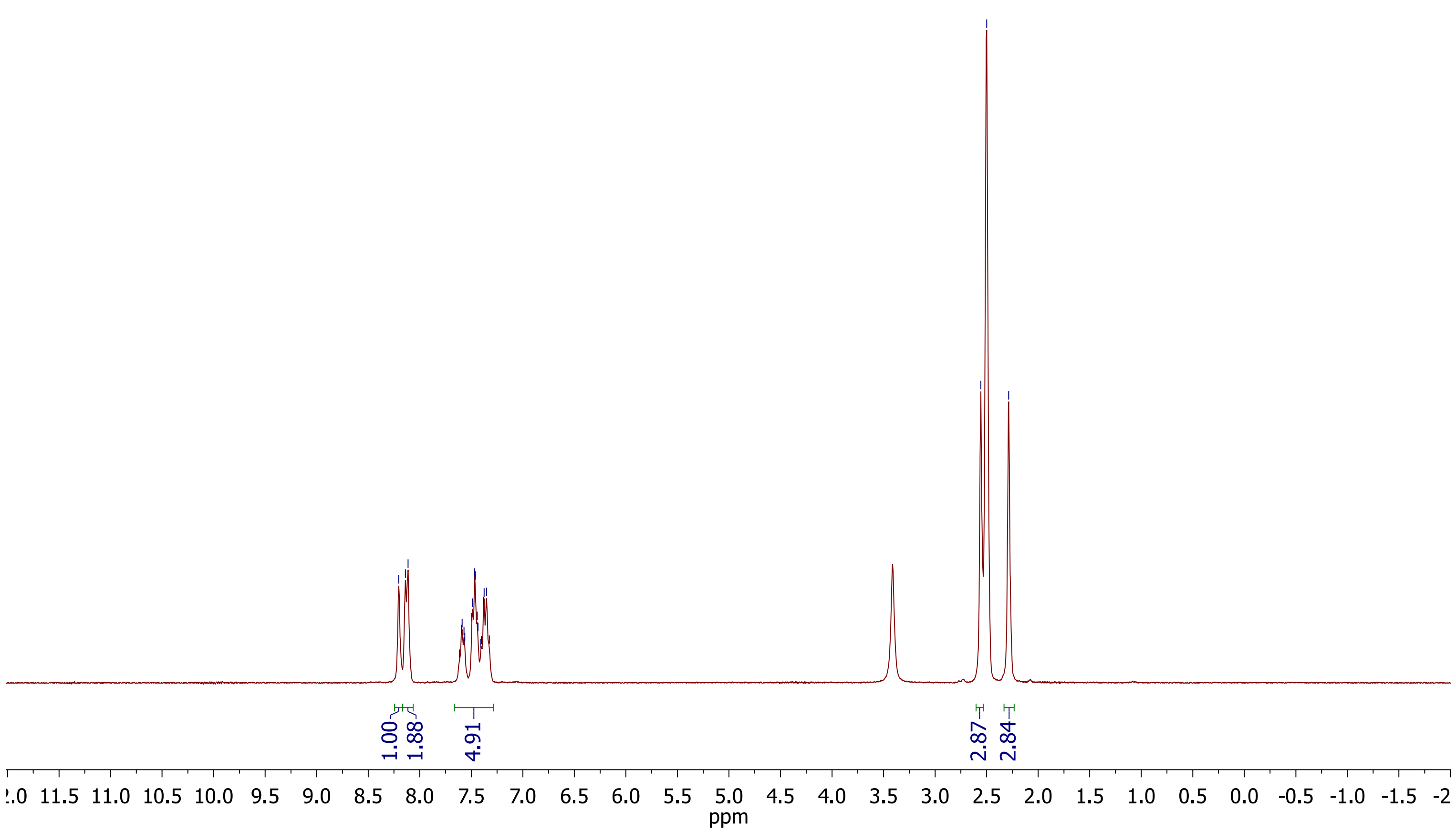

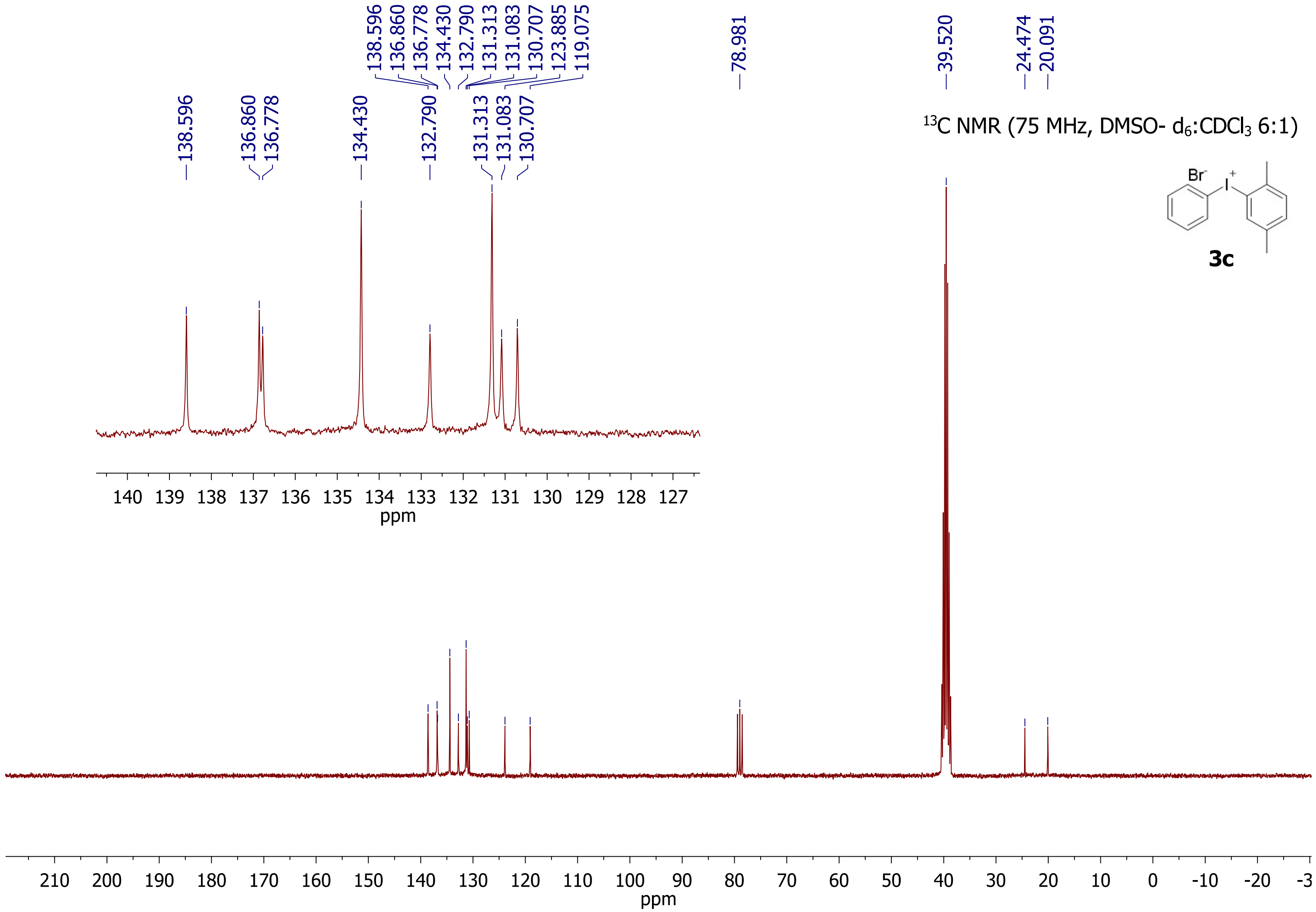

138.596
136.860
136.778
134.430
132.790
131.313
131.083
130.707
123.885
119.075
78.981
39.520
24.474
20.091
^{13}C NMR (75 MHz, DMSO- d_6:$CDCl_3$ 6:1)
Br⁻
I⁺
3c
140 139 138 137 136 135 134 133 132 131 130 129 128 127
ppm
210 200 190 180 170 160 150 140 130 120 110 100 90 80 70 60 50 40 30 20 10 0 -10 -20 -3
ppm

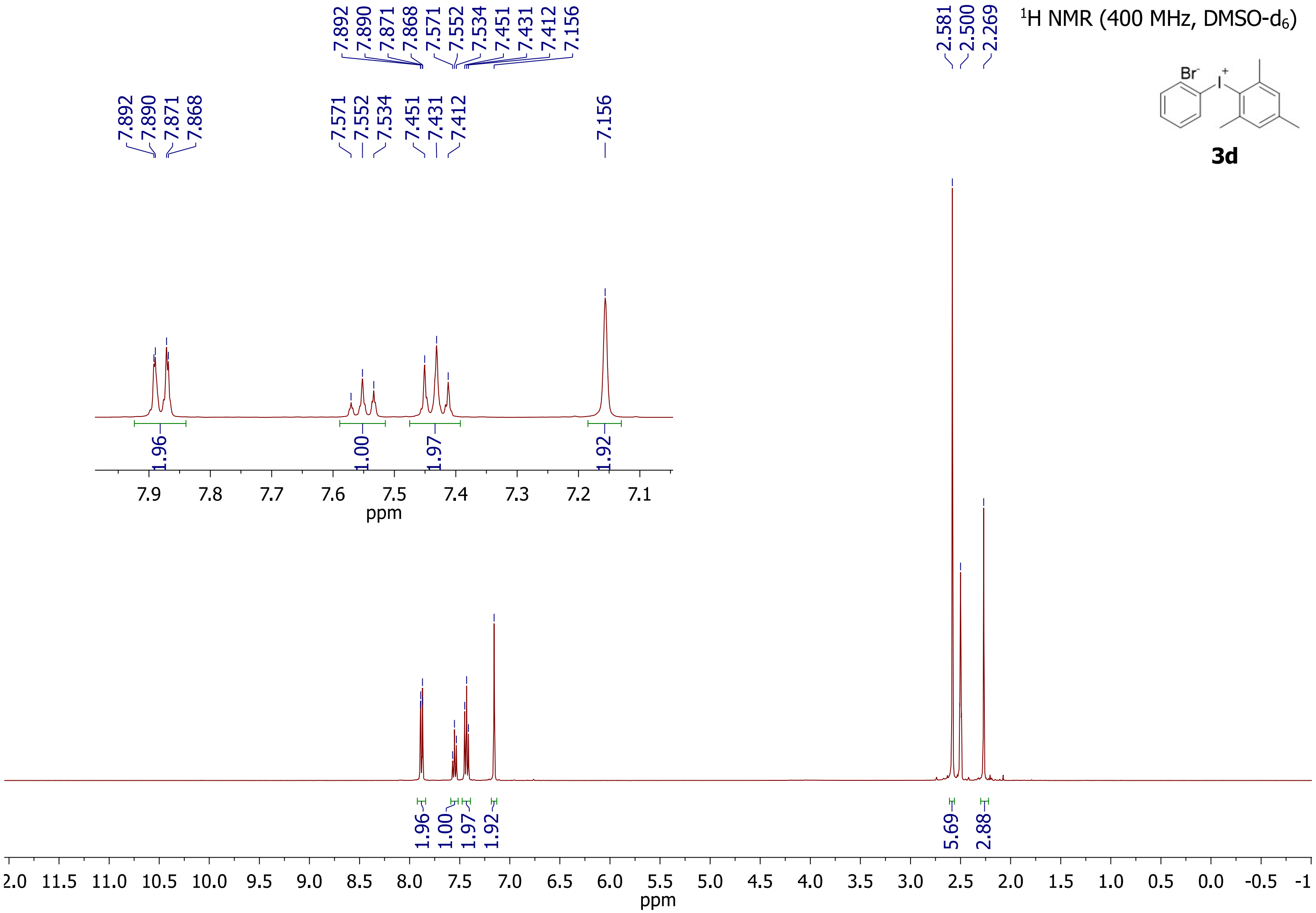

7.892
7.890
7.871
7.868
7.571
7.552
7.534
7.451
7.431
7.412
7.156
2.581
2.500
2.269
¹H NMR (400 MHz, DMSO-d6)
Br
I
1.96
1.00
1.97
1.92
5.69
2.88
ppm

3d

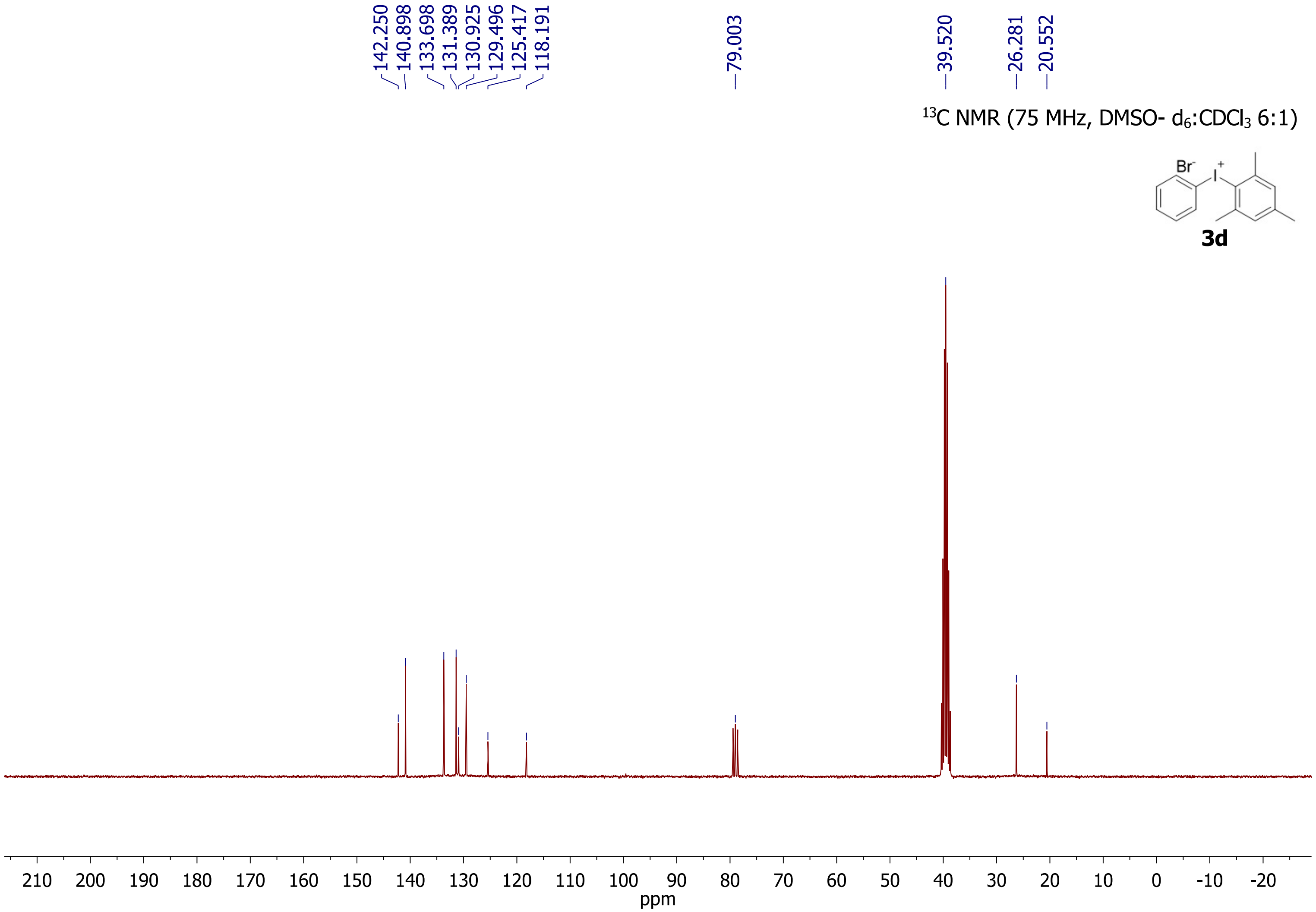
142.250
140.898
133.698
131.389
130.925
129.496
125.417
118.191
79.003
39.520
26.281
20.552
^{13}C NMR (75 MHz, DMSO- d_6:$CDCl_3$ 6:1)
Br⁻
I⁺
3d
210 200 190 180 170 160 150 140 130 120 110 100 90 80 70 60 50 40 30 20 10 0 -10 -20
ppm

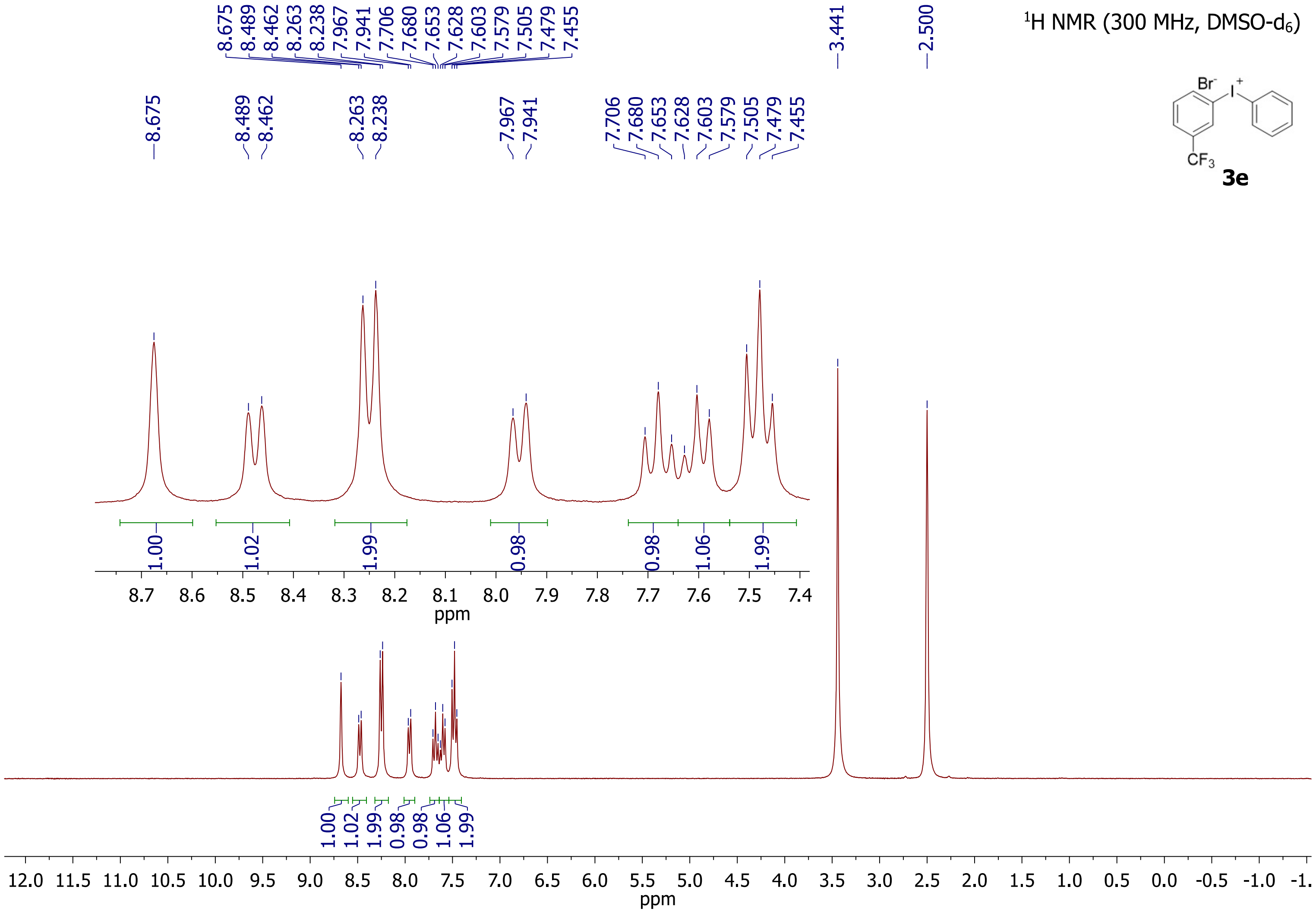
8.675
8.489
8.462
8.263
8.238
7.967
7.941
7.706
7.680
7.653
7.628
7.603
7.579
7.505
7.479
7.455
3.441
2.500
^{1}H NMR (300 MHz, DMSO-d$_6$)
Br$^-$
I$^+$
CF$_3$
3e
1.00
1.02
1.99
0.98
0.98
1.06
1.99
ppm

^{13}C NMR (75 MHz, DMSO-d_6)

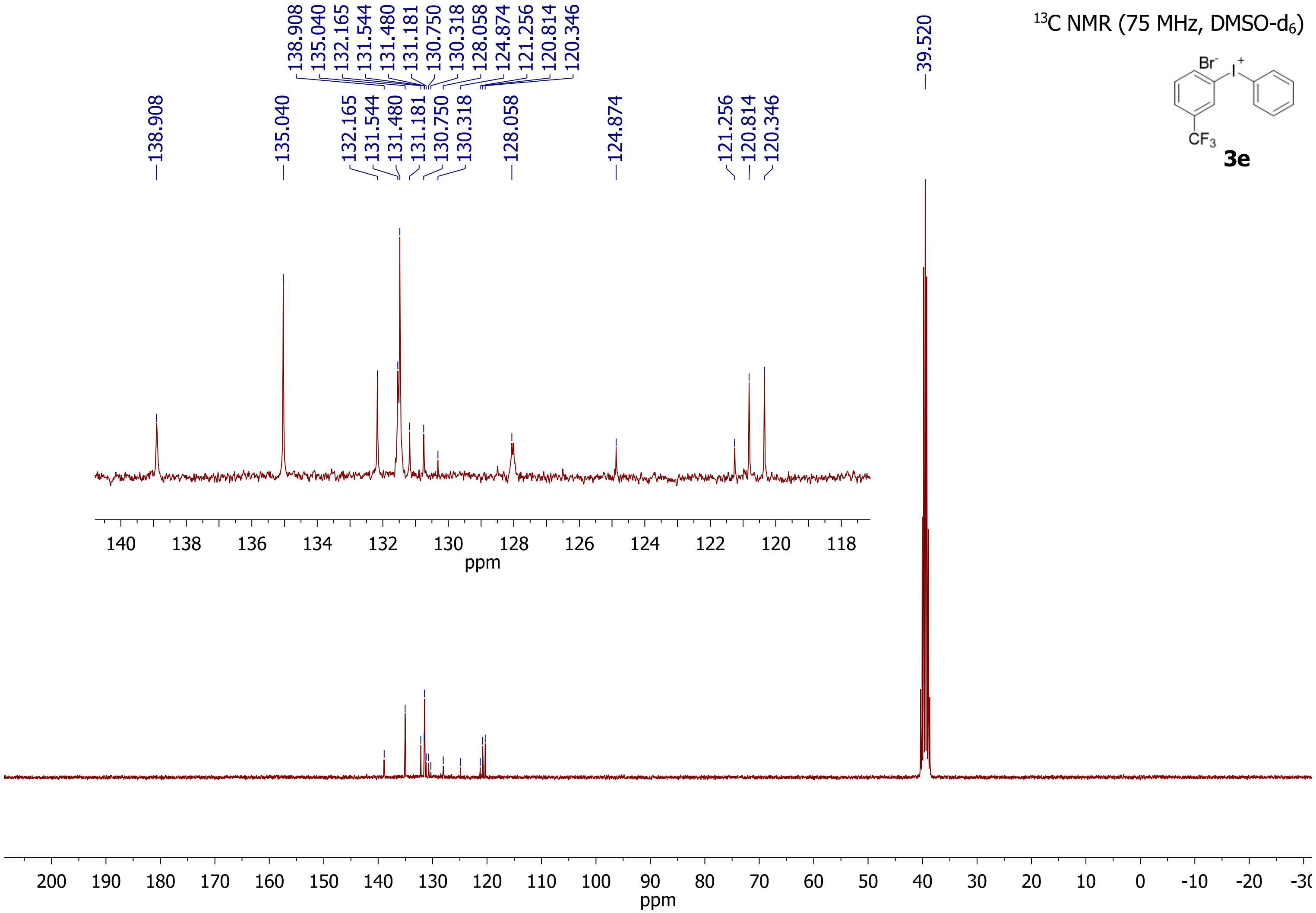

3e

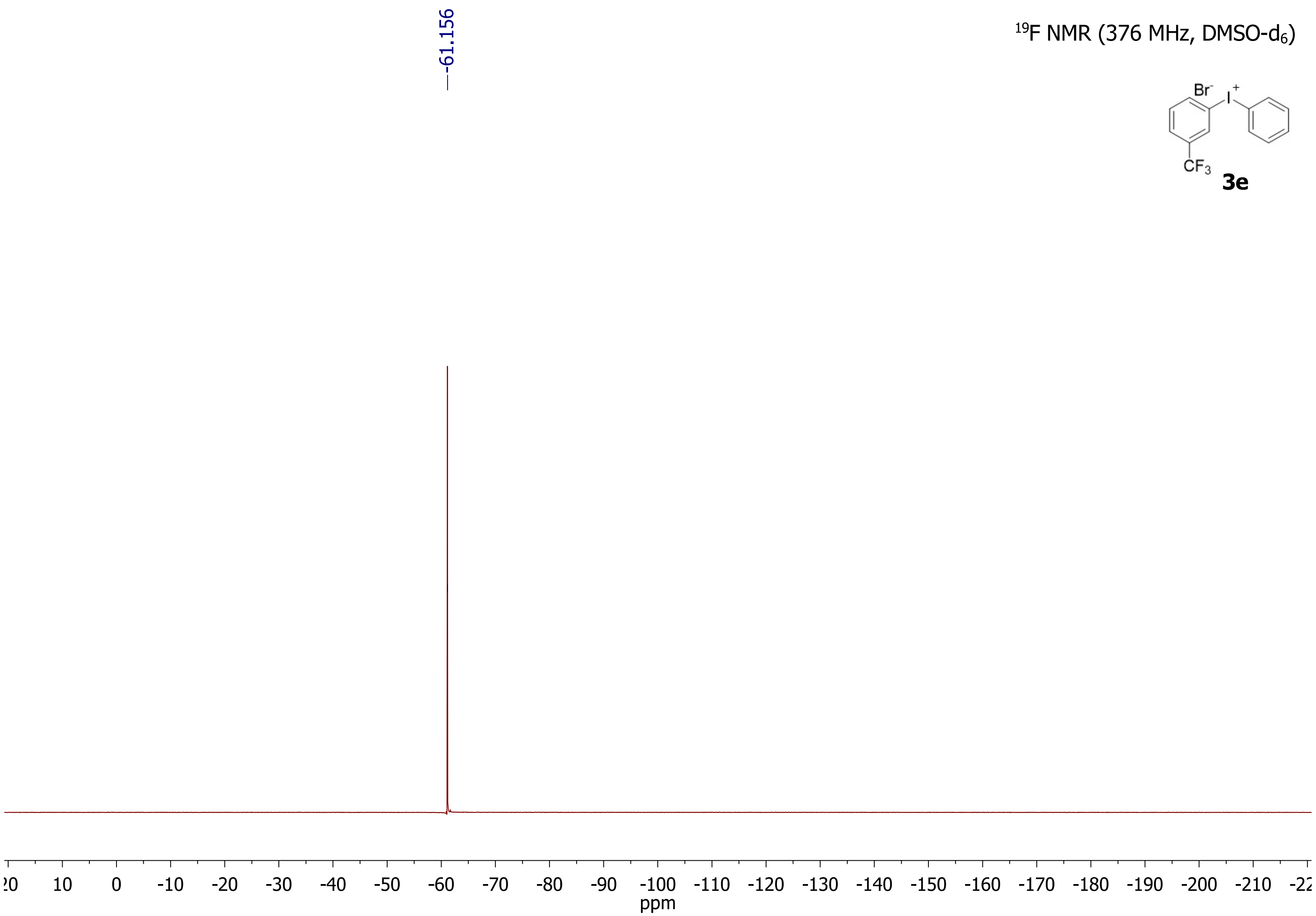
-61.156
19F NMR (376 MHz, DMSO-d6)
Br-
I+
CF3
3e
20 10 0 -10 -20 -30 -40 -50 -60 -70 -80 -90 -100 -110 -120 -130 -140 -150 -160 -170 -180 -190 -200 -210
ppm

^1H NMR (300 MHz, DMSO-d_6)

3f

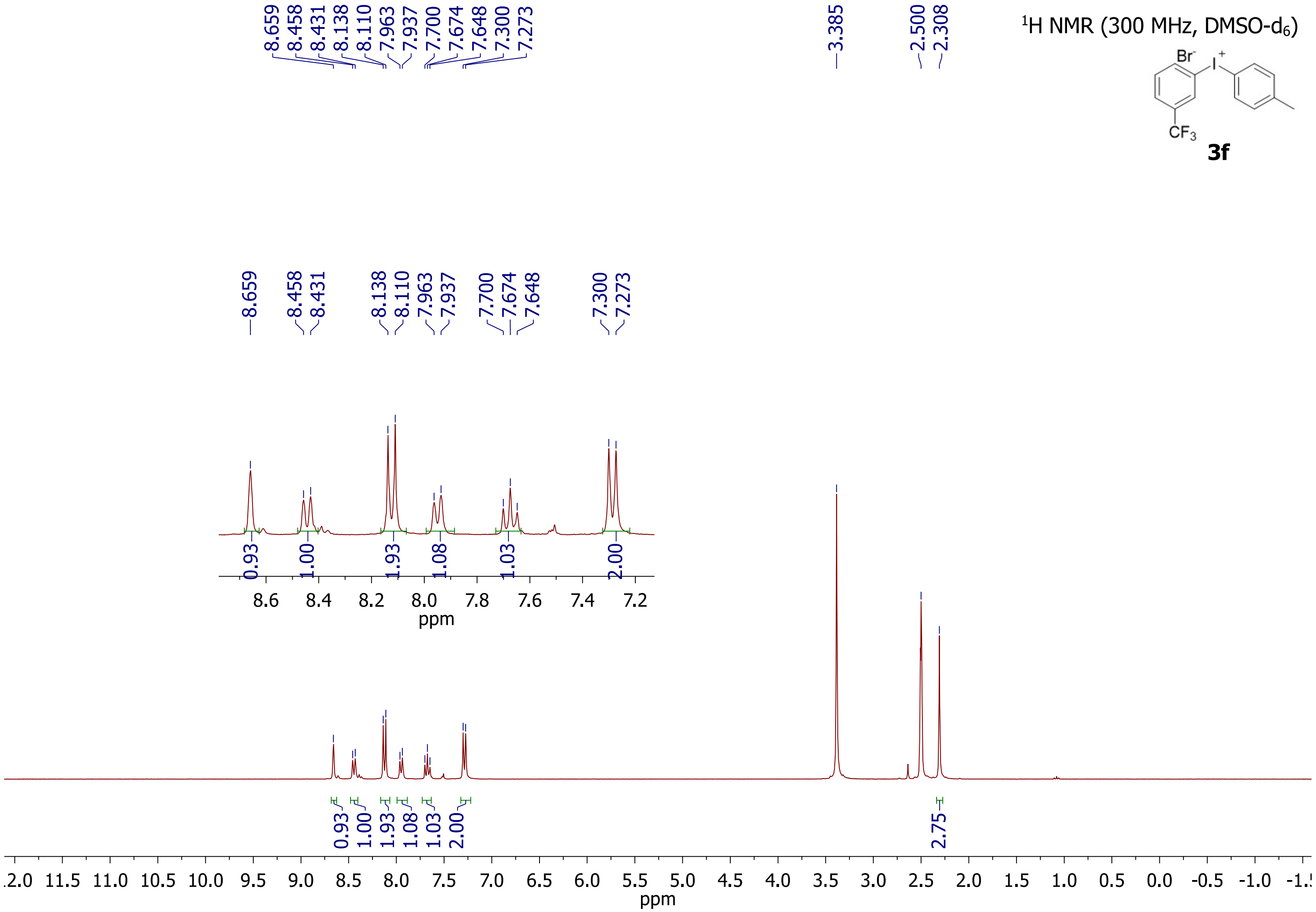

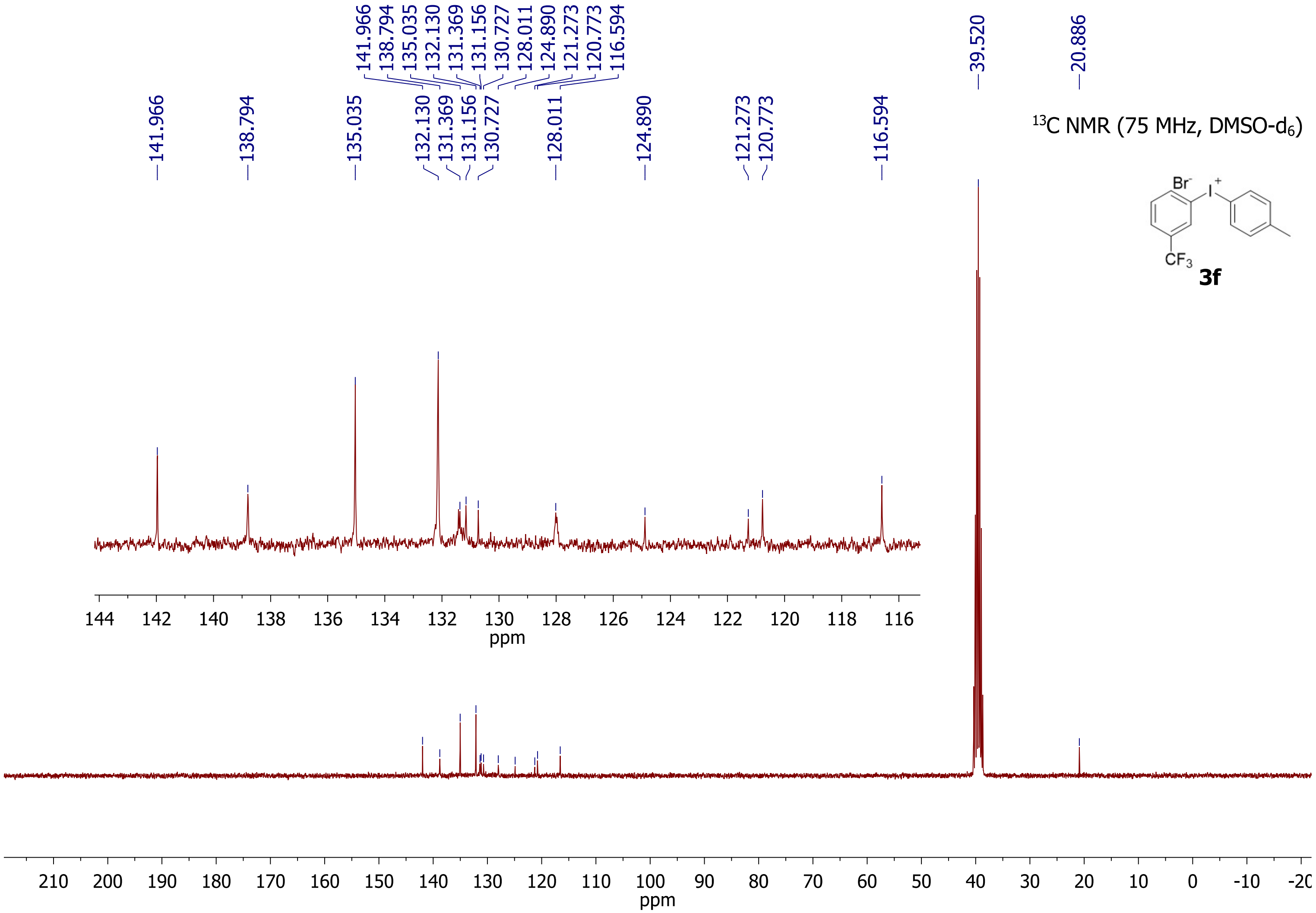

141.966
138.794
135.035
132.130
131.369
131.156
130.727
128.011
124.890
121.273
120.773
116.594
39.520
20.886
¹³C NMR (75 MHz, DMSO-d6)
Br⁻
I⁺
CF3
3f
ppm
ppm

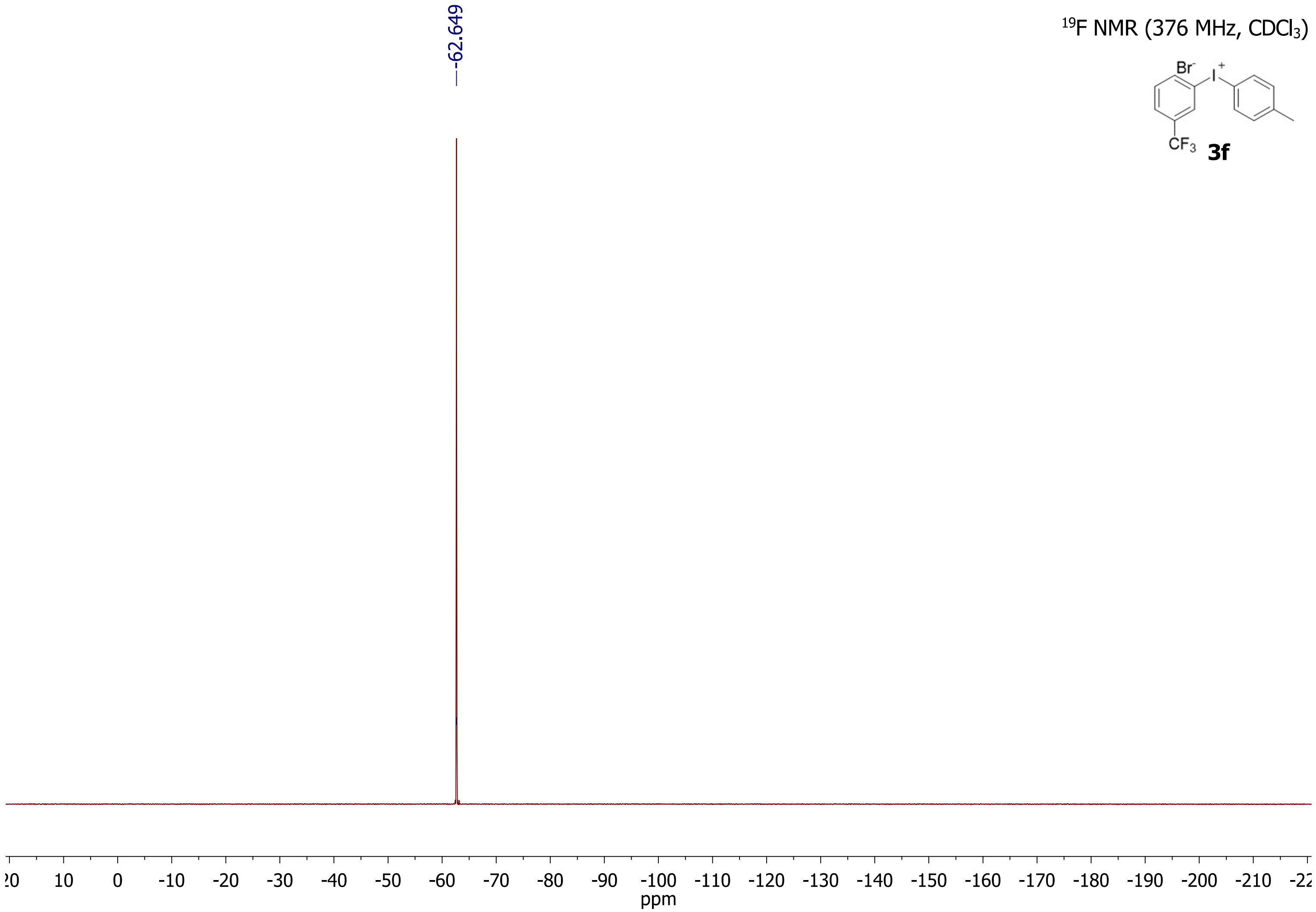

^{19}F NMR (376 MHz, $CDCl_3$)

3f

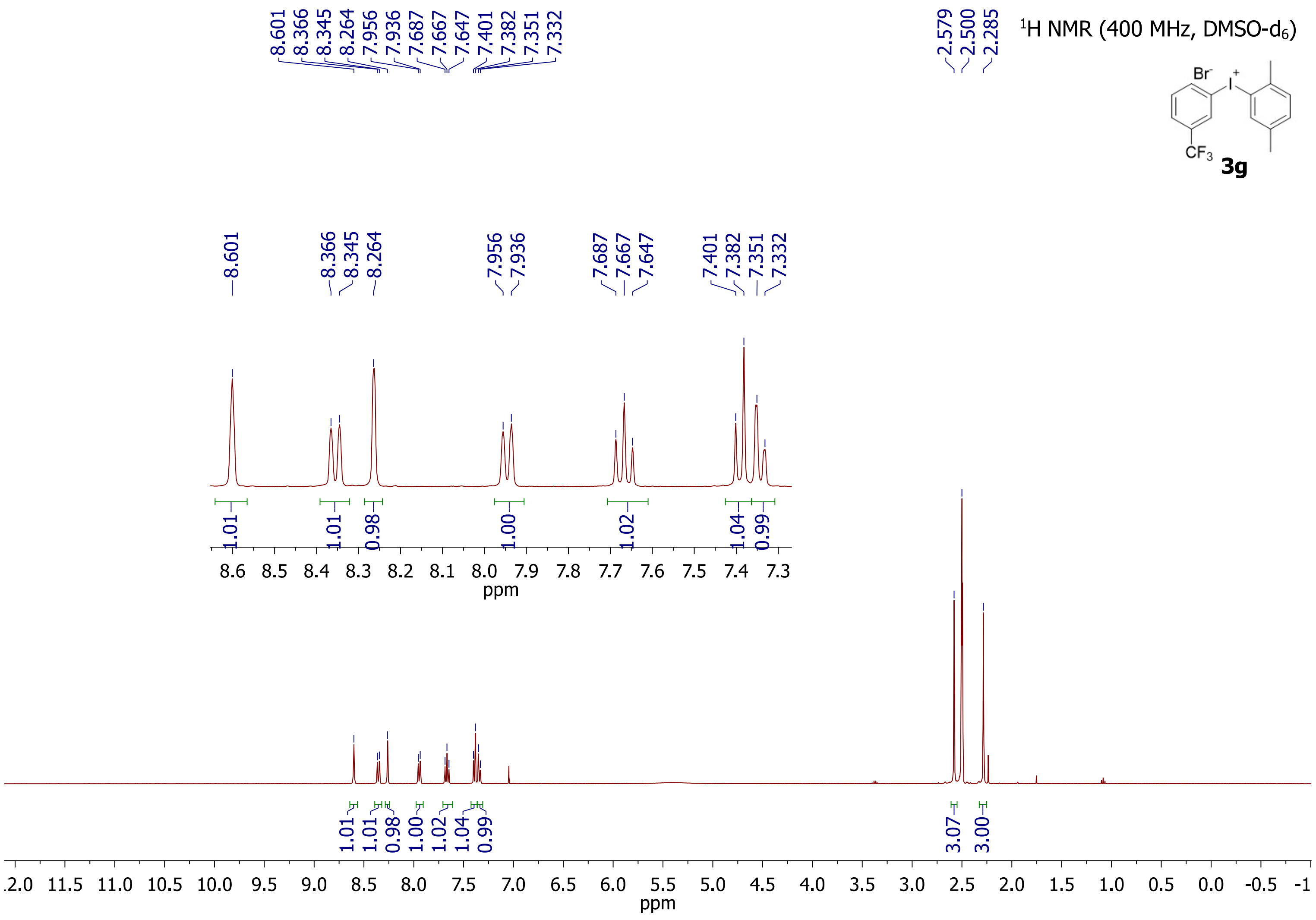
8.601
8.366
8.345
8.264
7.956
7.936
7.687
7.667
7.647
7.401
7.382
7.351
7.332
2.579
2.500
2.285
1H NMR (400 MHz, DMSO-d6)
Br-
I+
CF3
3g
1.01
1.01
0.98
1.00
1.02
1.04
0.99
3.07
3.00
ppm

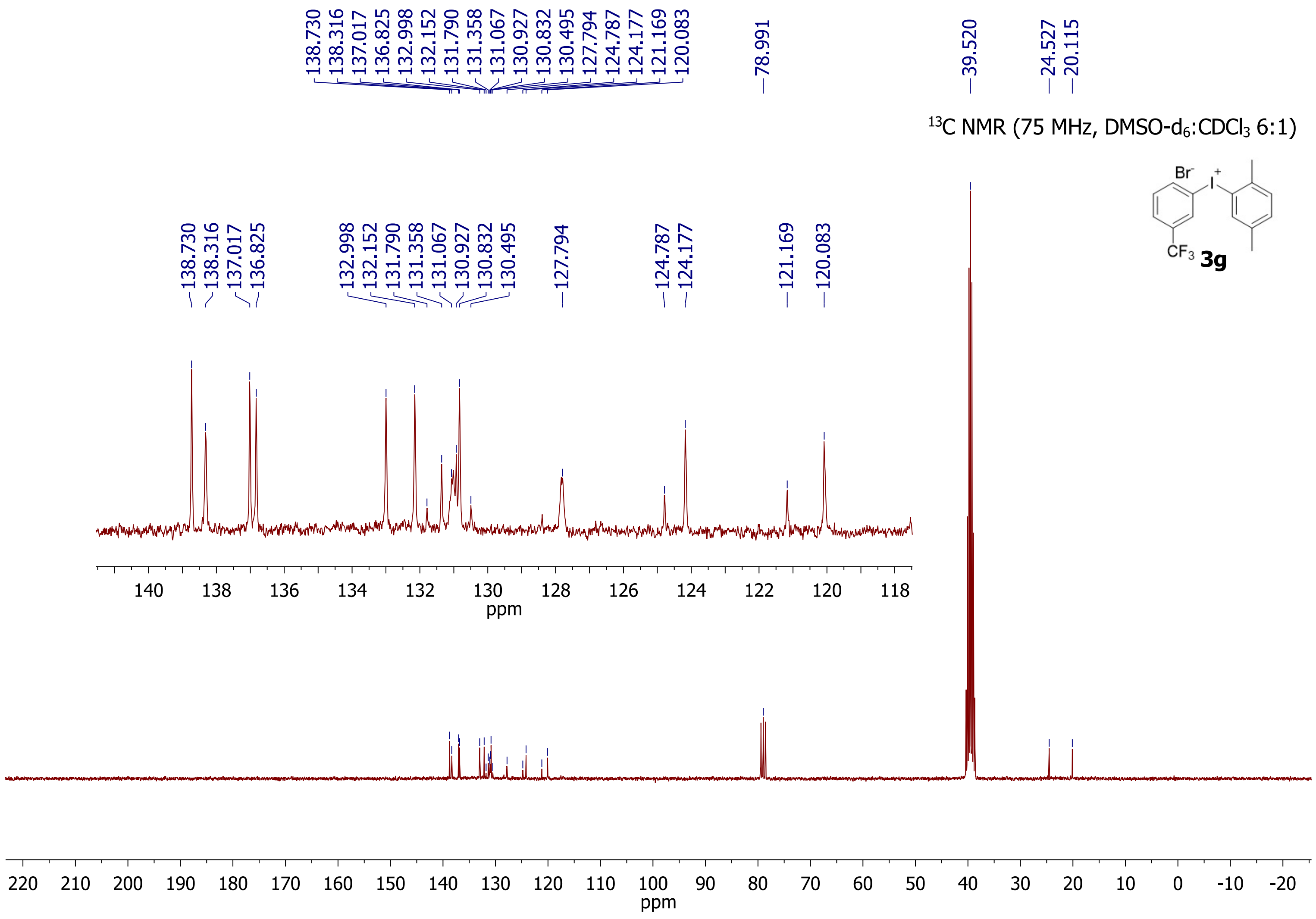

138.730
138.316
137.017
136.825
132.998
132.152
131.790
131.358
131.067
130.927
130.832
130.495
127.794
124.787
124.177
121.169
120.083
78.991
39.520
24.527
20.115
^{13}C NMR (75 MHz, DMSO-d$_6$:CDCl$_3$ 6:1)
Br⁻
I⁺
CF$_3$
3g
ppm

^{19}F NMR (376 MHz, DMSO-d_6)

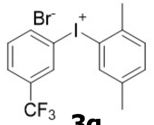

3g

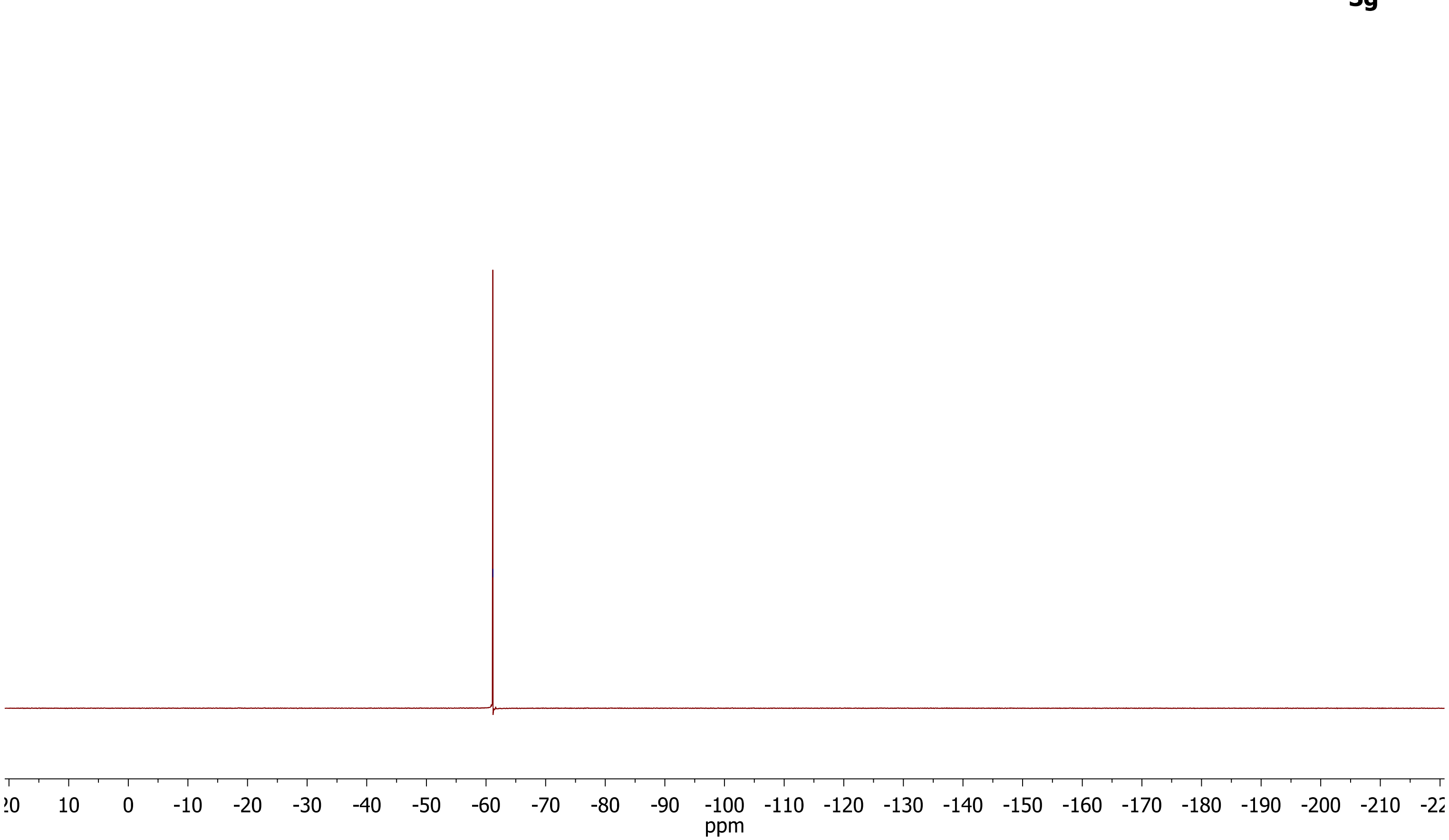

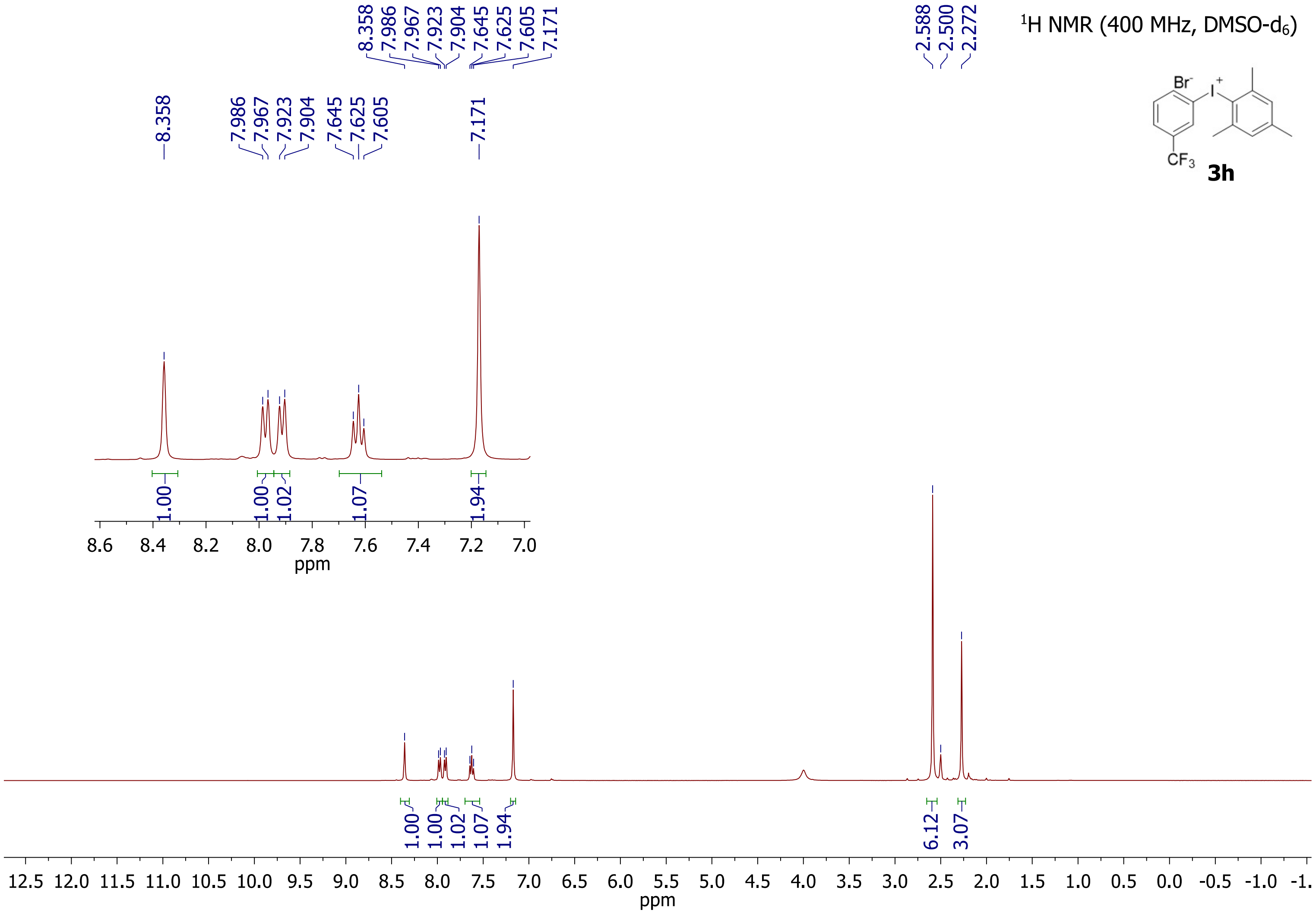

^{1}H NMR (400 MHz, DMSO-d6)
3h

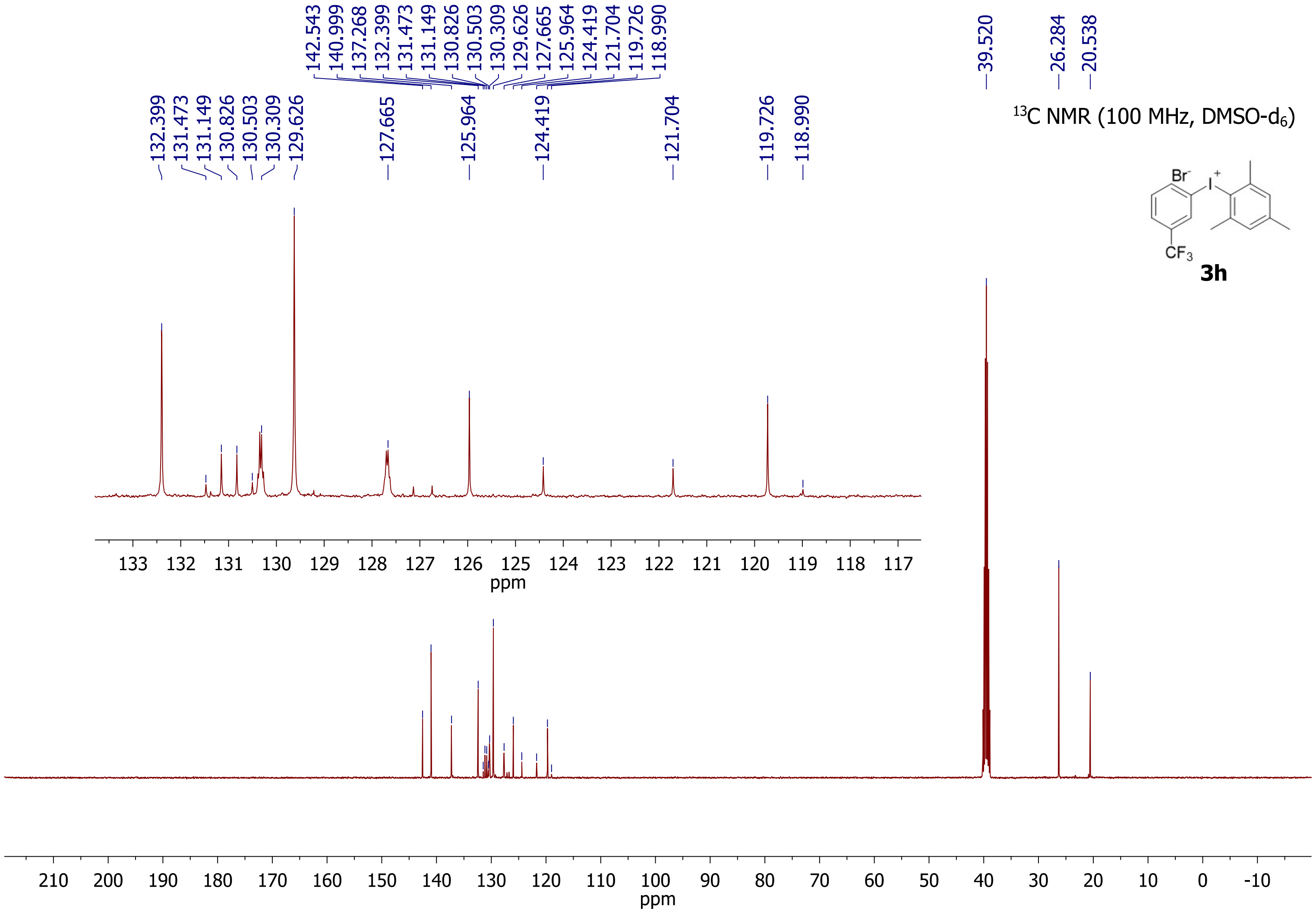
142.543
140.999
137.268
132.399
131.473
131.149
130.826
130.503
130.309
129.626
127.665
125.964
124.419
121.704
119.726
118.990
39.520
26.284
20.538
^{13}C NMR (100 MHz, DMSO-d$_6$)
Br$^-$
I$^+$
CF$_3$
3h
ppm
ppm

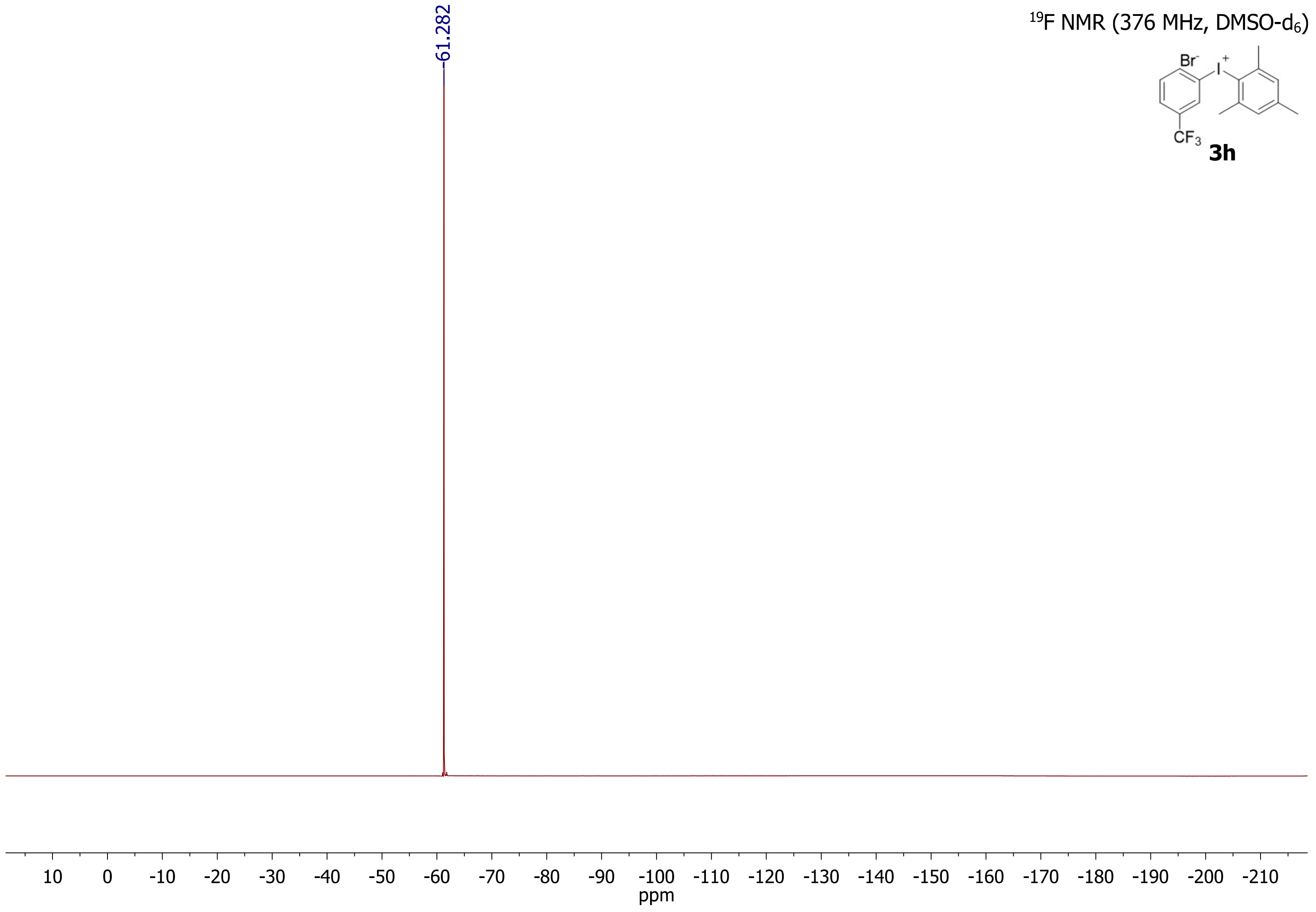
-61.282
19F NMR (376 MHz, DMSO-d6)
Br-
I+
CF3
3h
10 0 -10 -20 -30 -40 -50 -60 -70 -80 -90 -100 -110 -120 -130 -140 -150 -160 -170 -180 -190 -200 -210
ppm

^1H NMR (300 MHz, DMSO-d_6)

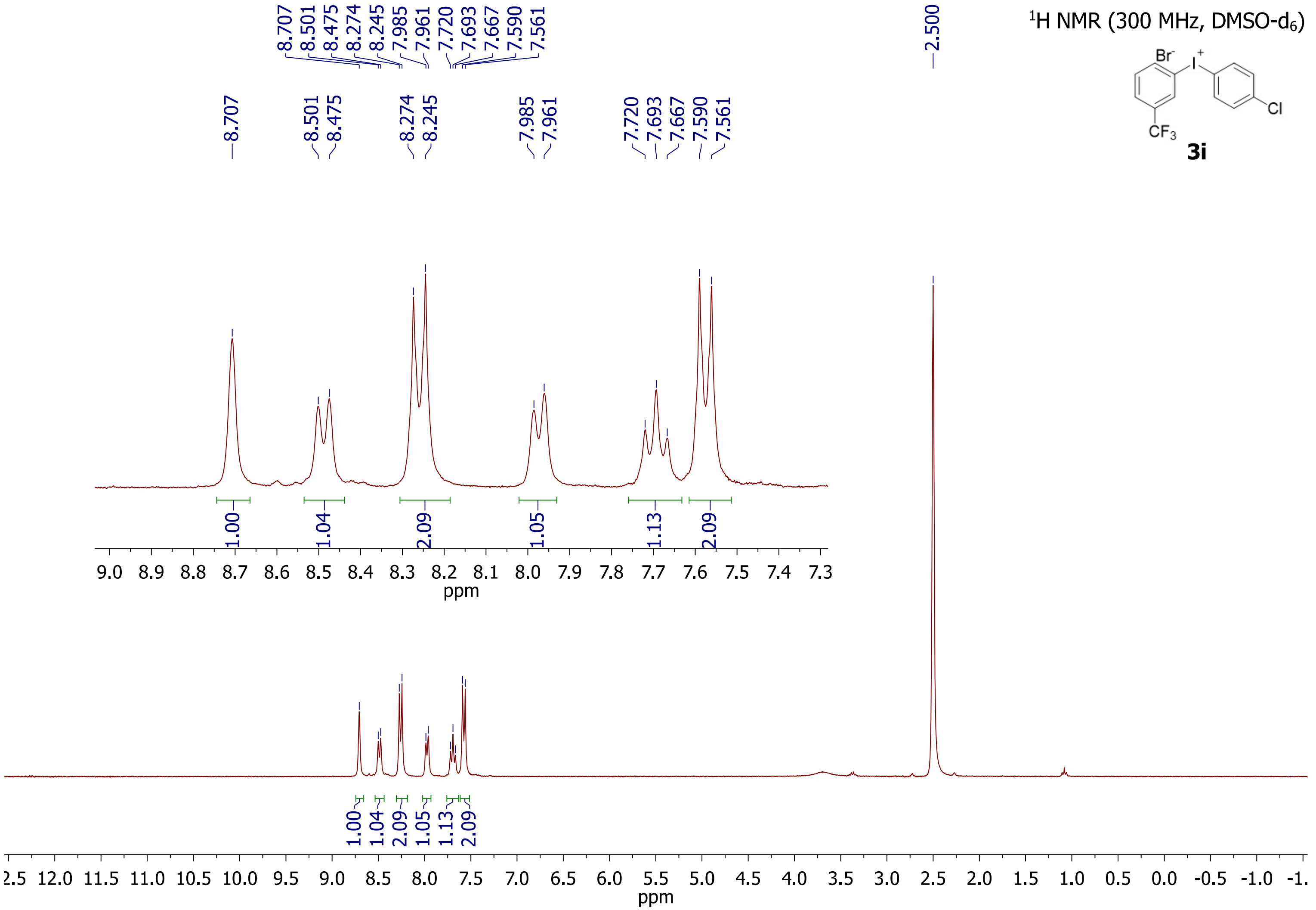

^{13}C NMR (75 MHz, DMSO-d_6)

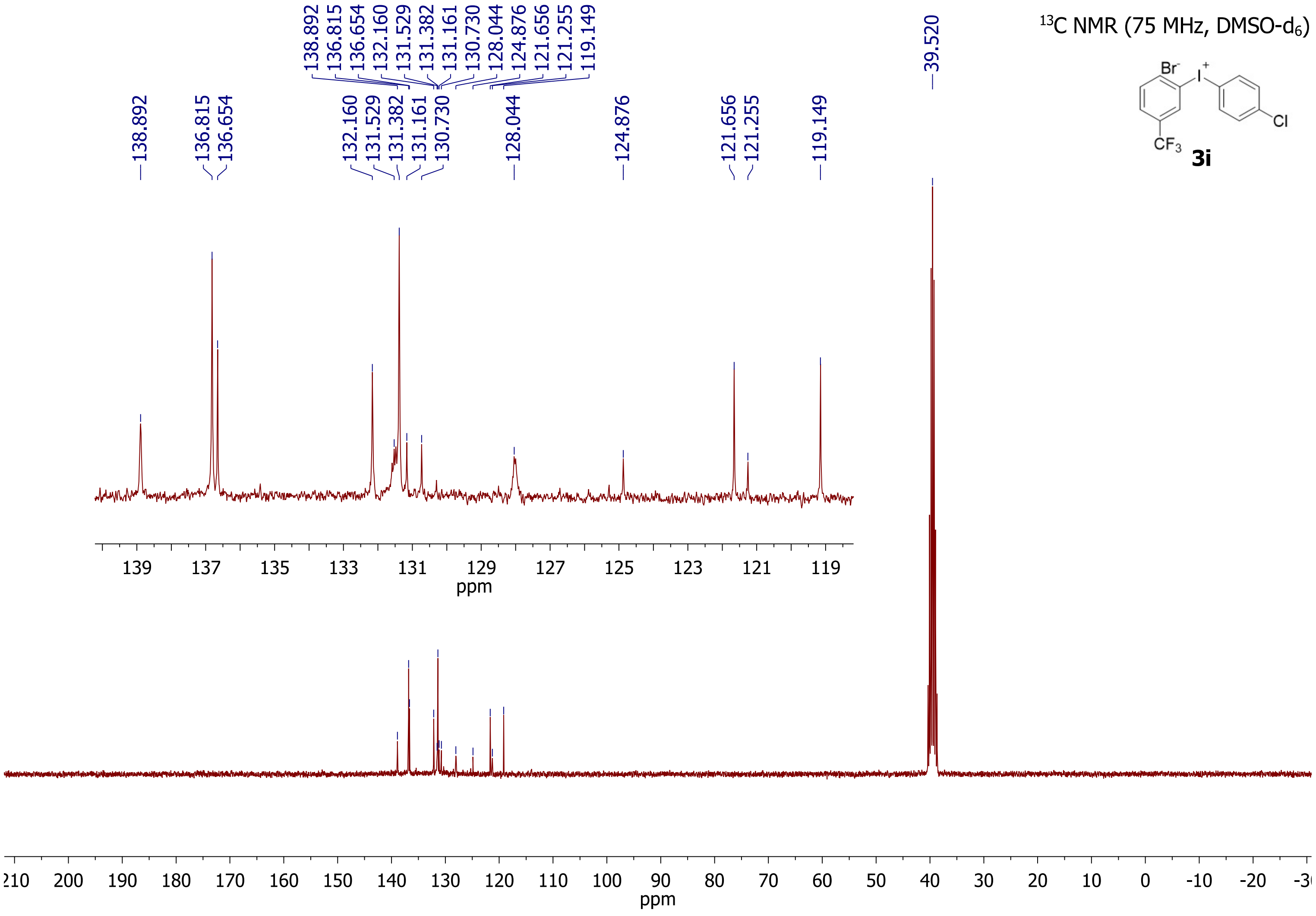

^{19}F NMR (376 MHz, DMSO-d$_6$)

3i

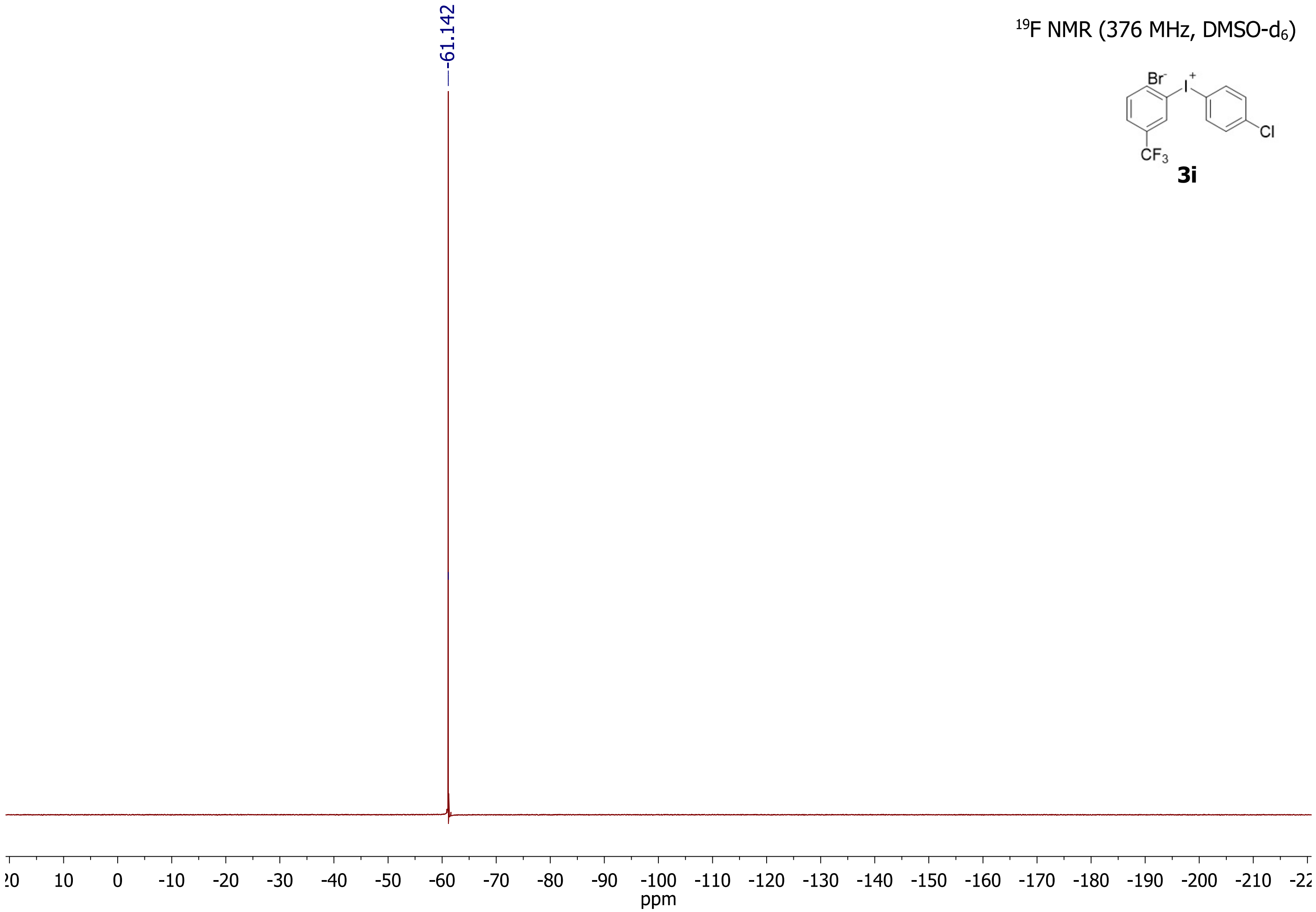

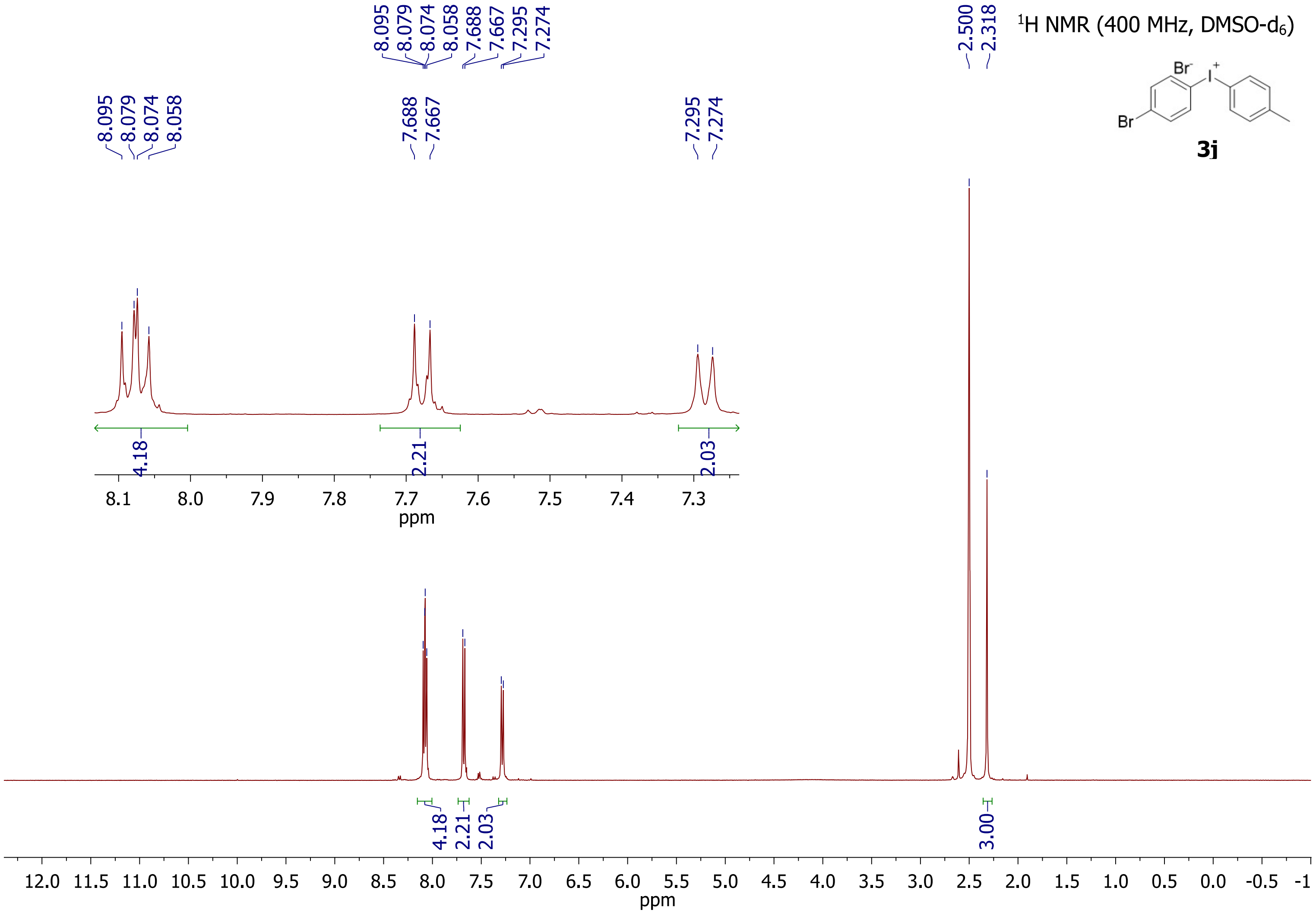
8.095
8.079
8.074
8.058
7.688
7.667
7.295
7.274
2.500
2.318
^{1}H NMR (400 MHz, DMSO-d6)
Br-
I+
Br
3j
4.18
2.21
2.03
3.00
ppm

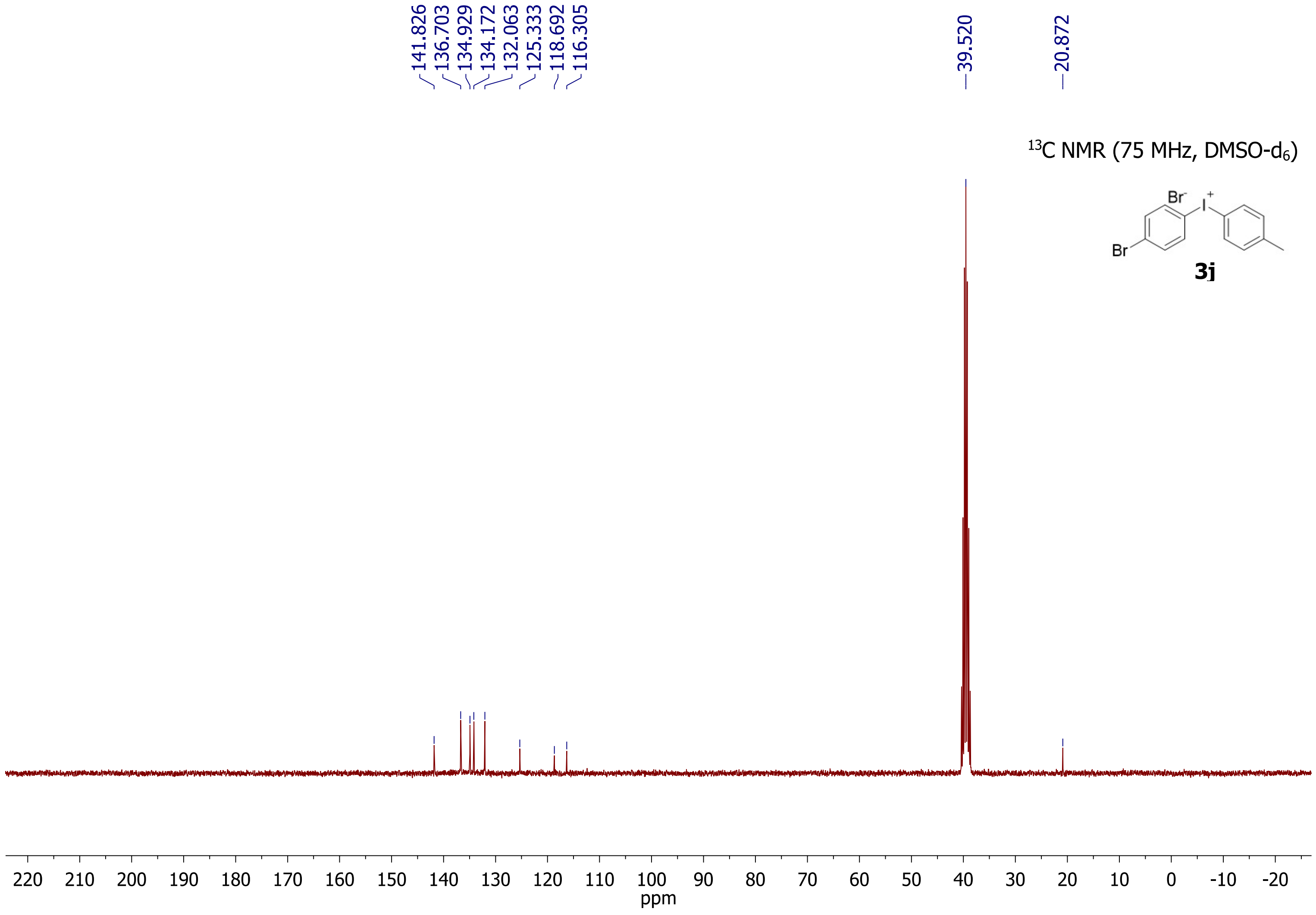
141.826
136.703
134.929
134.172
132.063
125.333
118.692
116.305
39.520
20.872
^{13}C NMR (75 MHz, DMSO-d$_6$)
Br⁻
I⁺
Br
3j
220 210 200 190 180 170 160 150 140 130 120 110 100 90 80 70 60 50 40 30 20 10 0 -10 -20
ppm

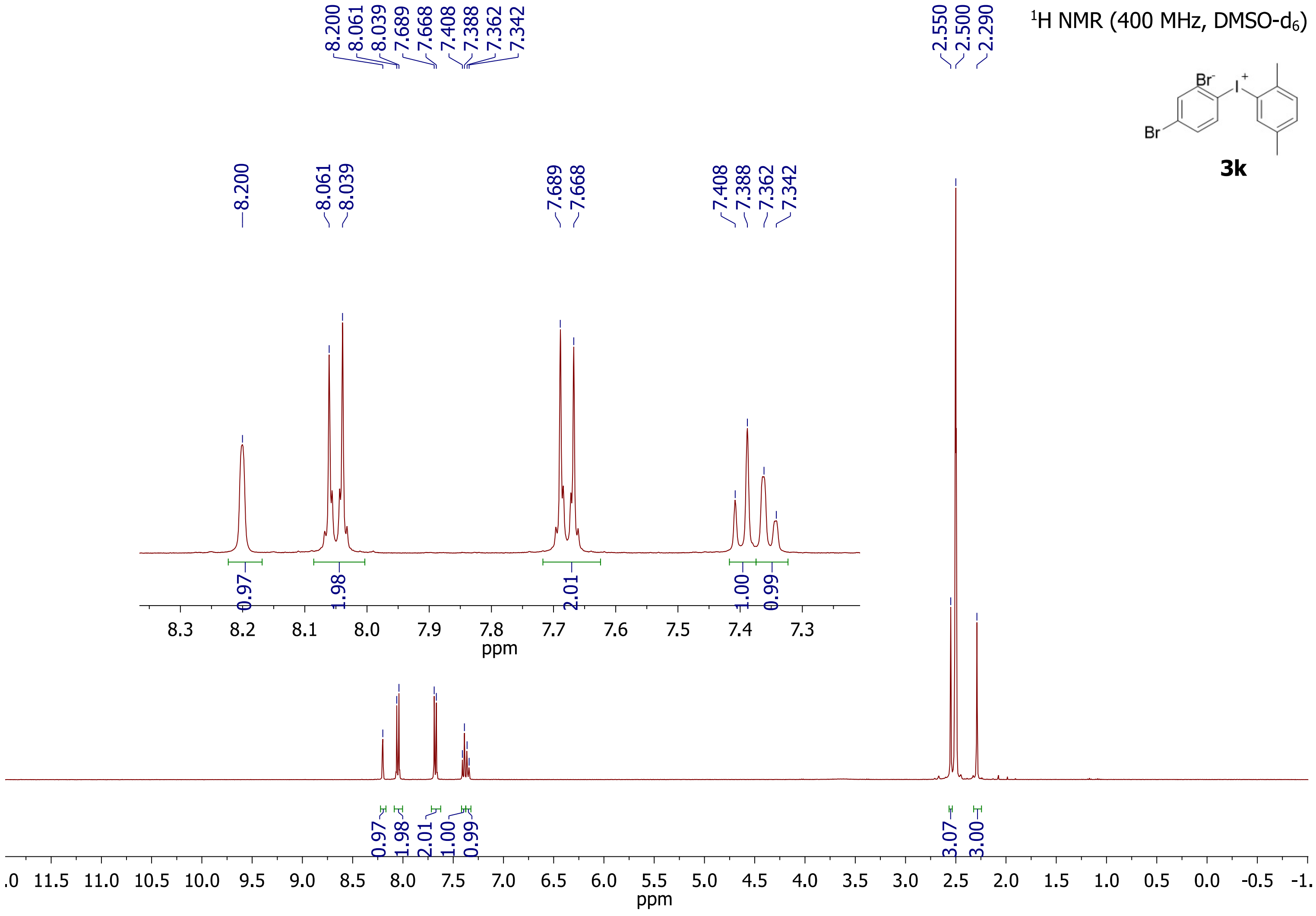

^{1}H NMR (400 MHz, DMSO-d6)
Br
I
Br
3k
8.200
8.061
8.039
7.689
7.668
7.408
7.388
7.362
7.342
2.550
2.500
2.290
0.97
1.98
2.01
1.00
0.99
3.07
3.00
ppm

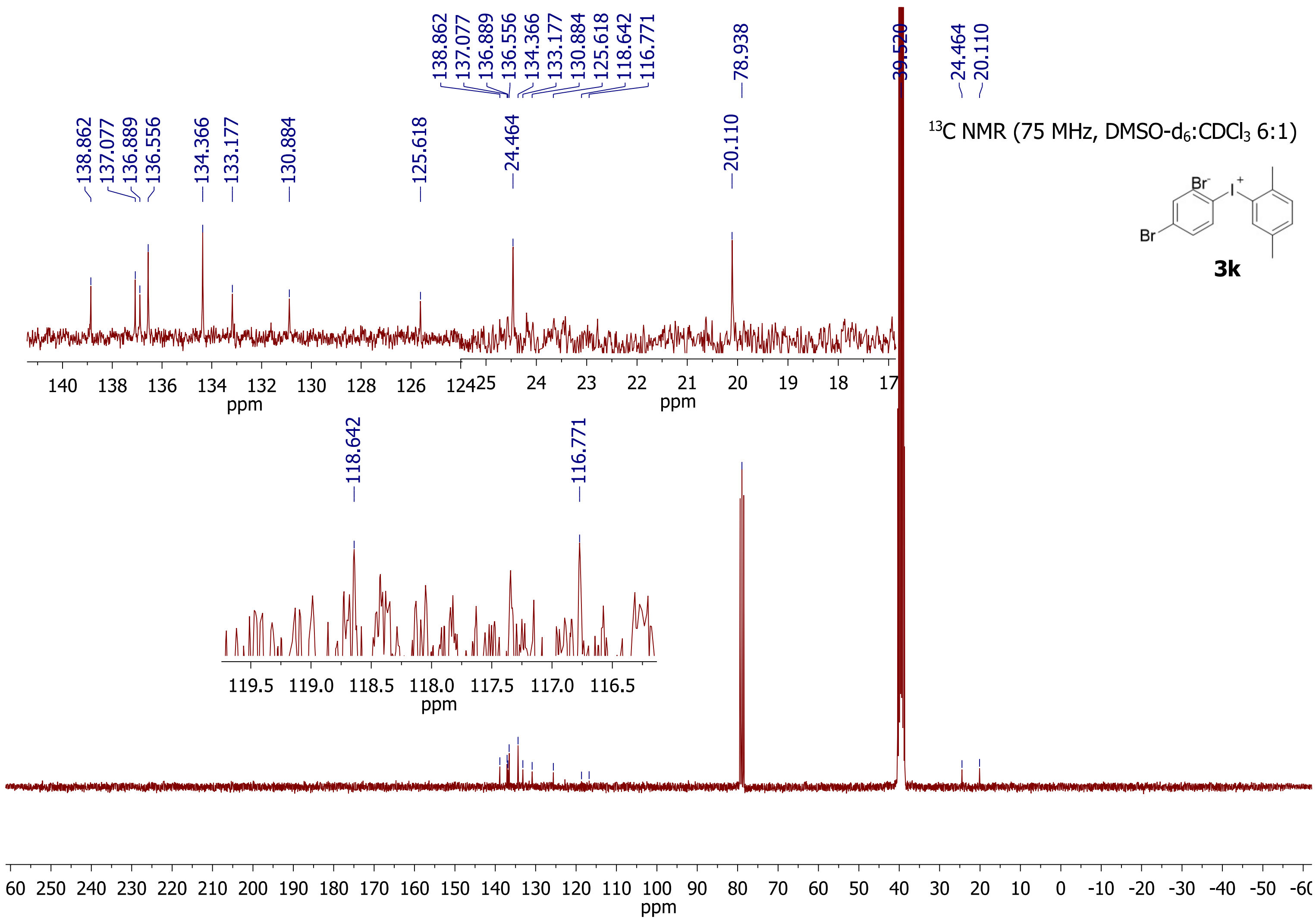

138.862
137.077
136.889
136.556
134.366
133.177
130.884
125.618
118.642
116.771
78.938
39.520
24.464
20.110
13C NMR (75 MHz, DMSO-d6:CDCl3 6:1)
Br-
I+
Br
3k
ppm

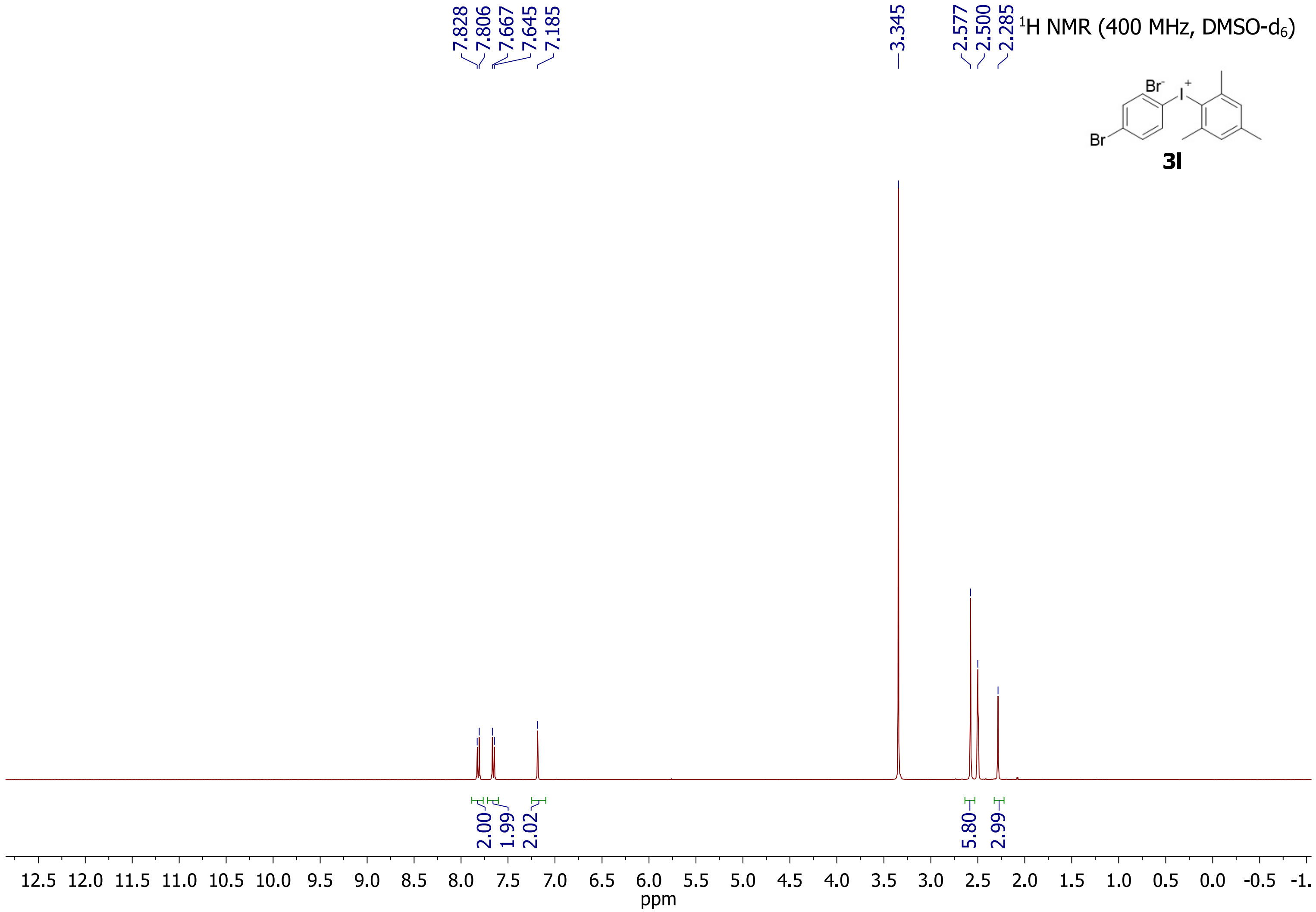
7.828
7.806
7.667
7.645
7.185
3.345
2.577
2.500
2.285
¹H NMR (400 MHz, DMSO-d6)
Br⁻
I⁺
Br
3l
2.00
1.99
2.02
5.80
2.99
12.5 12.0 11.5 11.0 10.5 10.0 9.5 9.0 8.5 8.0 7.5 7.0 6.5 6.0 5.5 5.0 4.5 4.0 3.5 3.0 2.5 2.0 1.5 1.0 0.5 0.0 -0.5 -1.
ppm

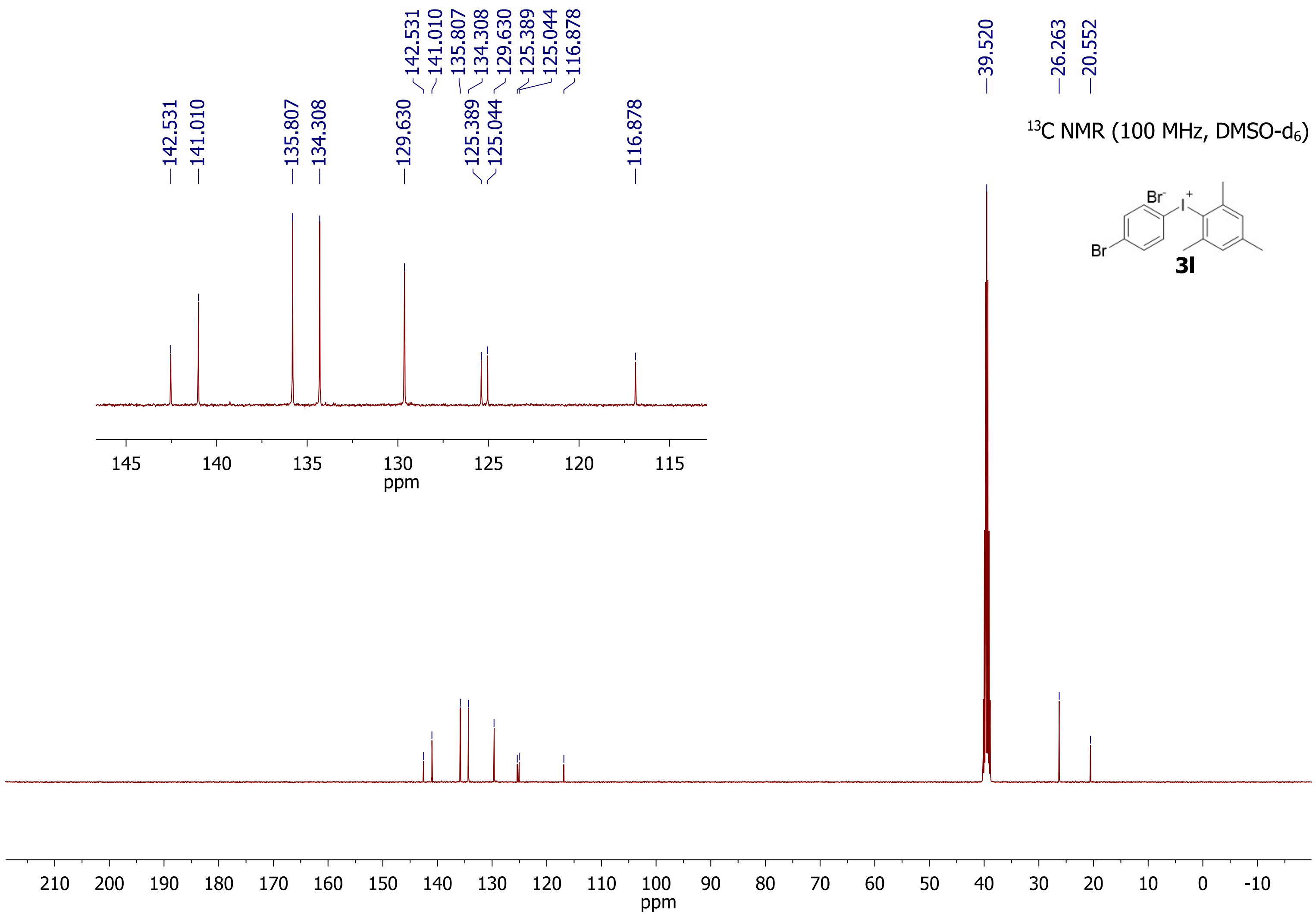
142.531
141.010
135.807
134.308
129.630
125.389
125.044
116.878
39.520
26.263
20.552
^{13}C NMR (100 MHz, DMSO-d$_6$)
Br⁻
I⁺
Br
3l
145 140 135 130 125 120 115
ppm
210 200 190 180 170 160 150 140 130 120 110 100 90 80 70 60 50 40 30 20 10 0 -10
ppm

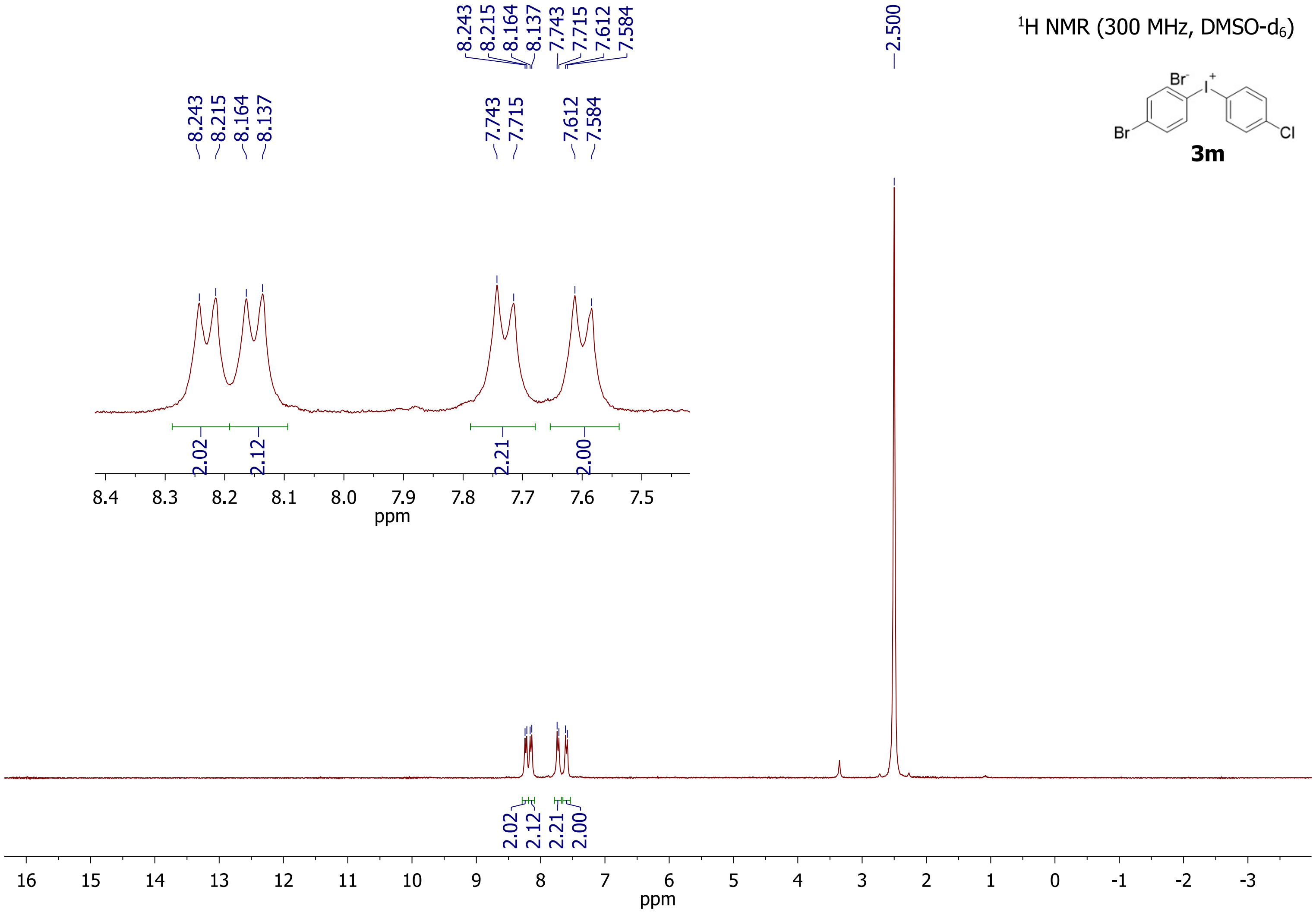

^{1}H NMR (300 MHz, DMSO-d_6)
3m
Br
Cl
8.243
8.215
8.164
8.137
7.743
7.715
7.612
7.584
2.500
2.02
2.12
2.21
2.00
ppm

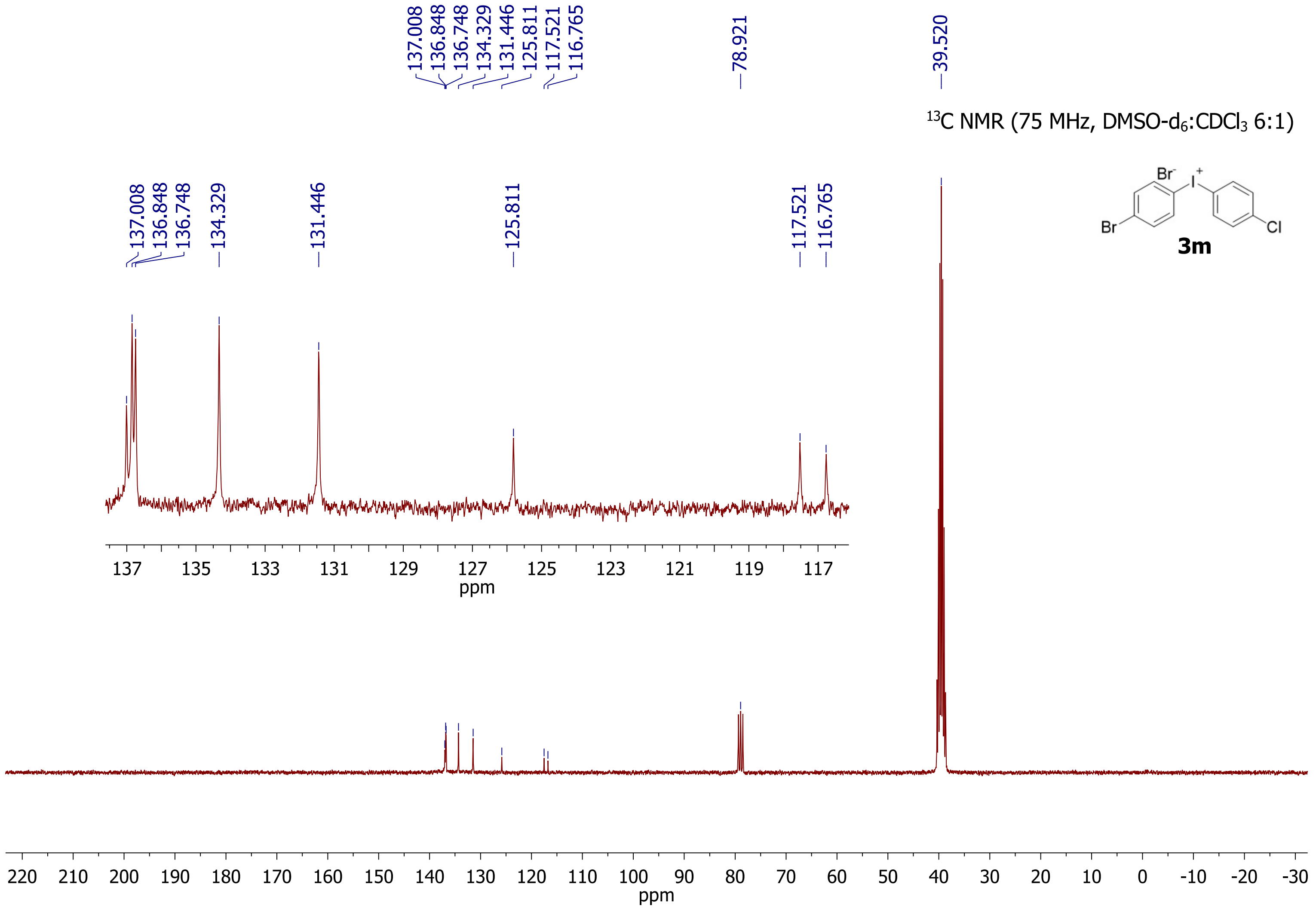
137.008
136.848
136.748
134.329
131.446
125.811
117.521
116.765
78.921
39.520
13C NMR (75 MHz, DMSO-d6:CDCl3 6:1)
Br-
I+
Br
Cl
3m
ppm

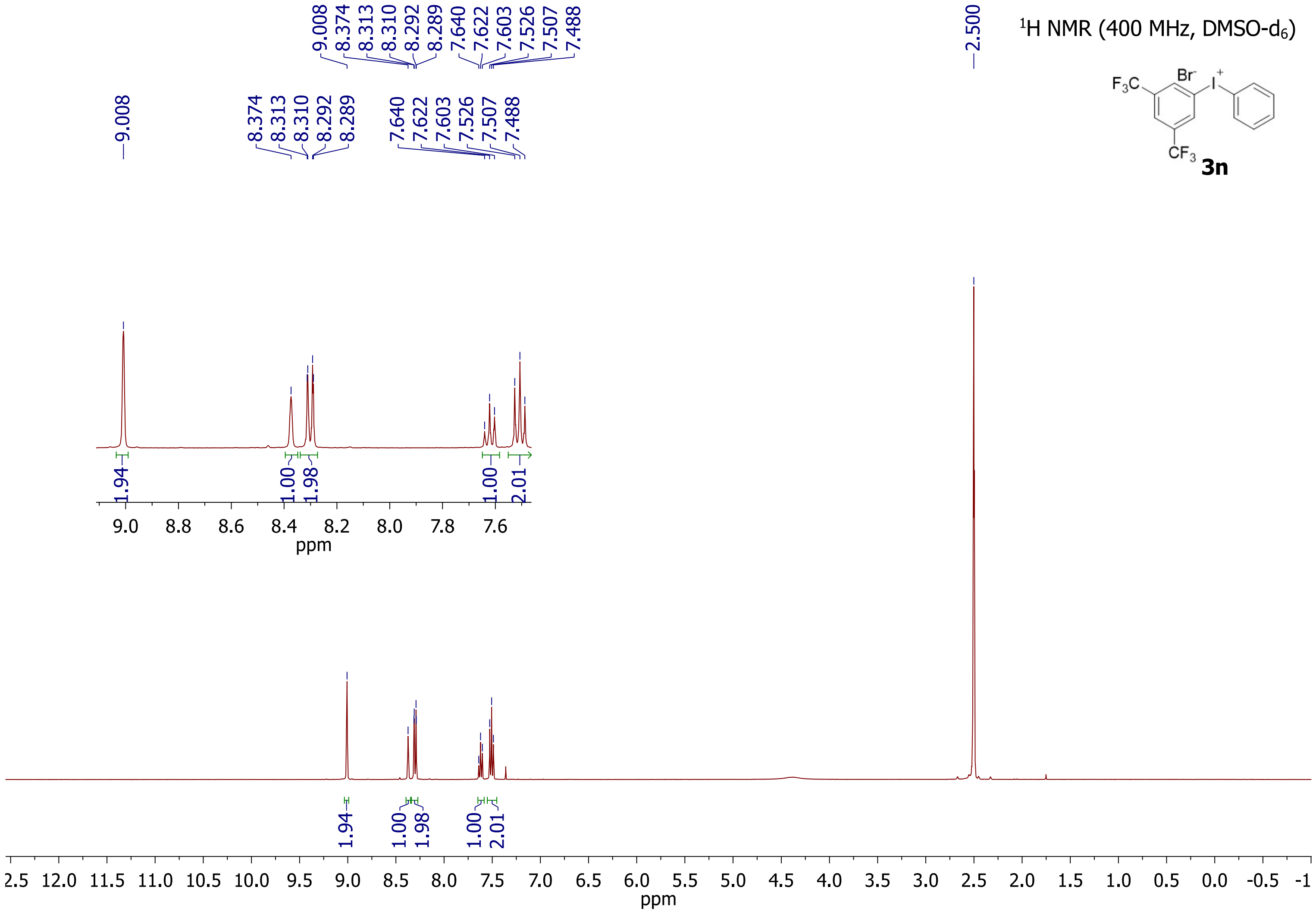

^1H NMR (400 MHz, DMSO-d_6)

3n

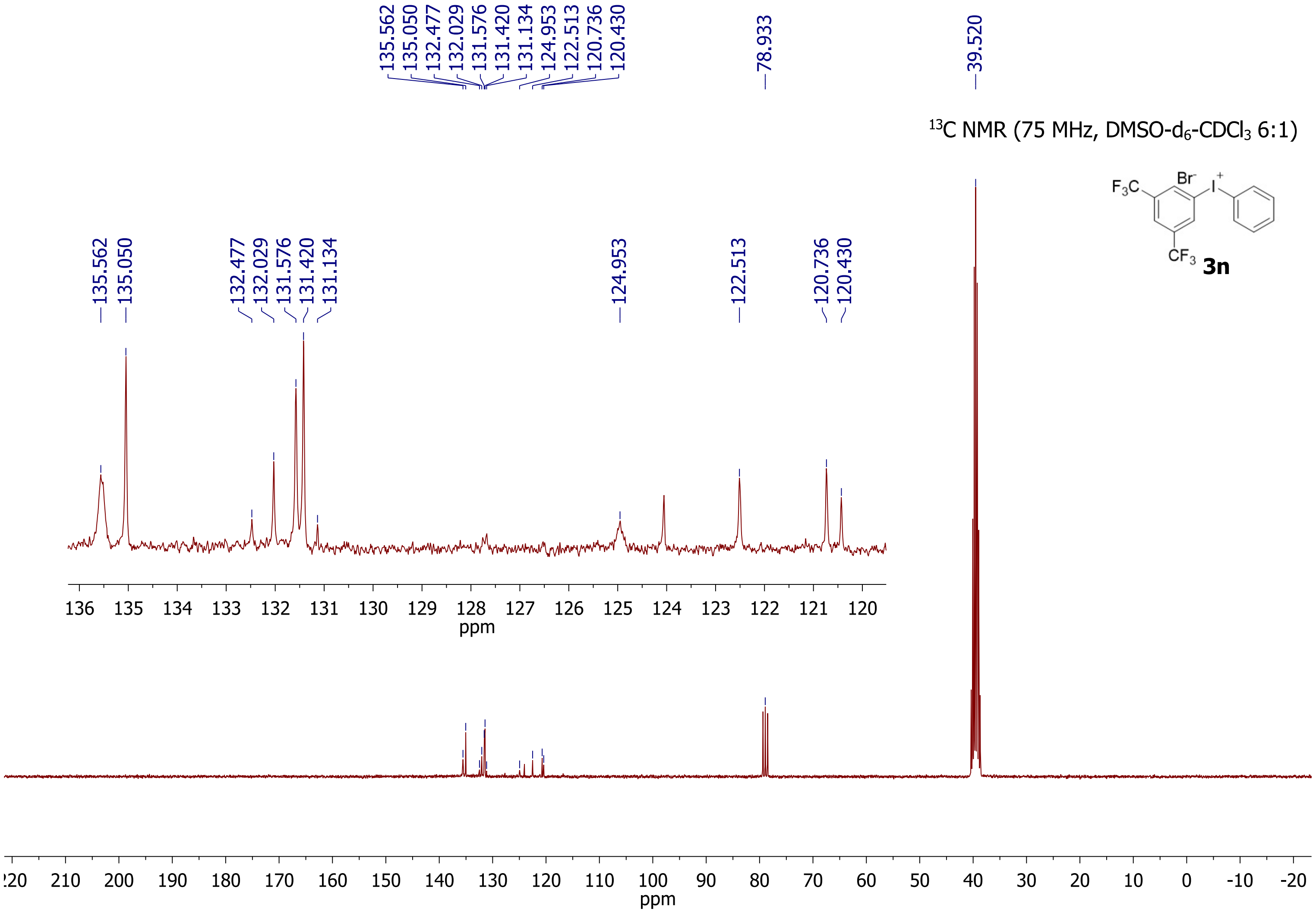

^{13}C NMR (75 MHz, DMSO-d_6-$CDCl_3$ 6:1)

3n

^{19}F NMR (376 MHz, DMSO-d_6)

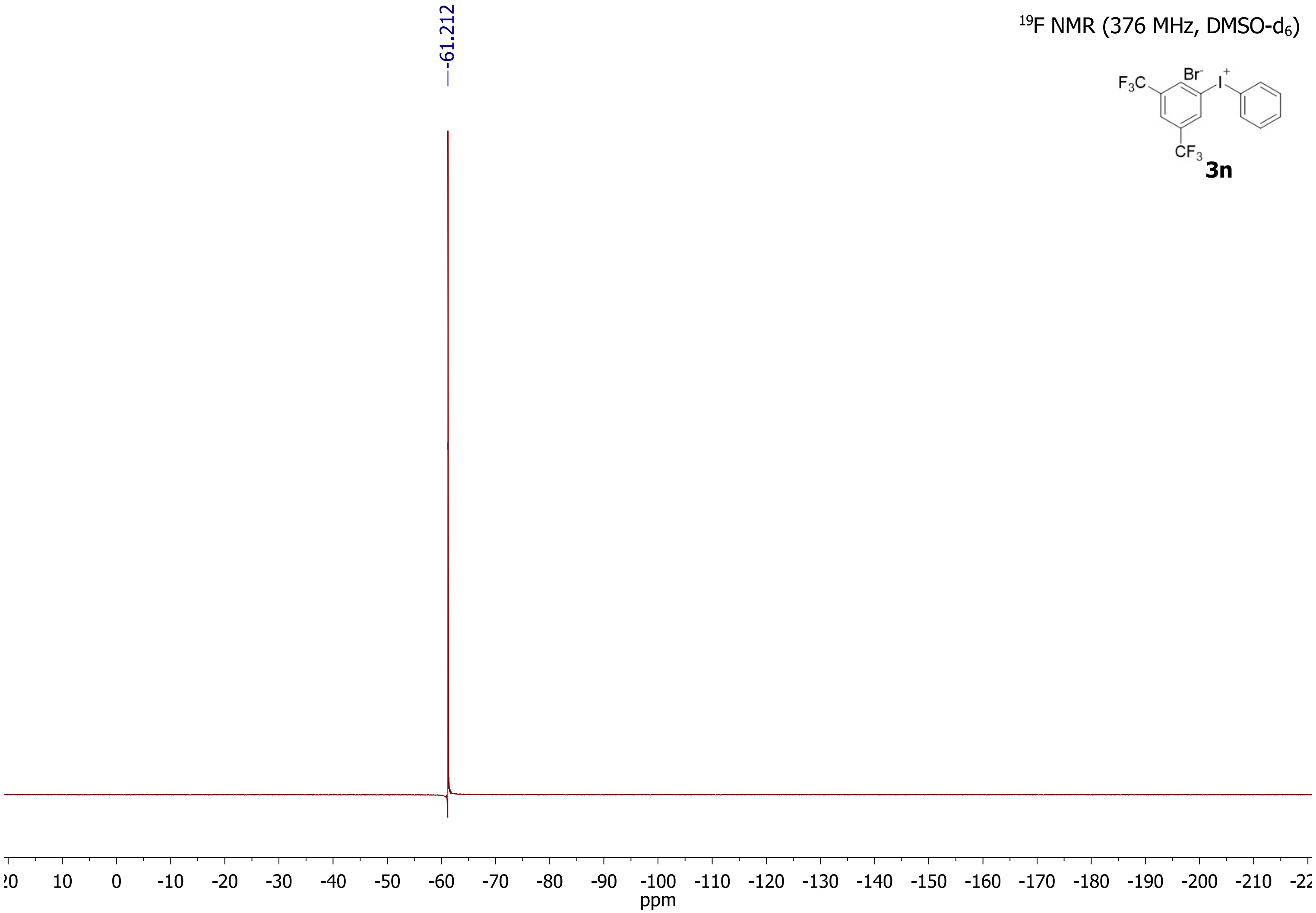

3n

^{1}H NMR (400 MHz, DMSO-d_6)

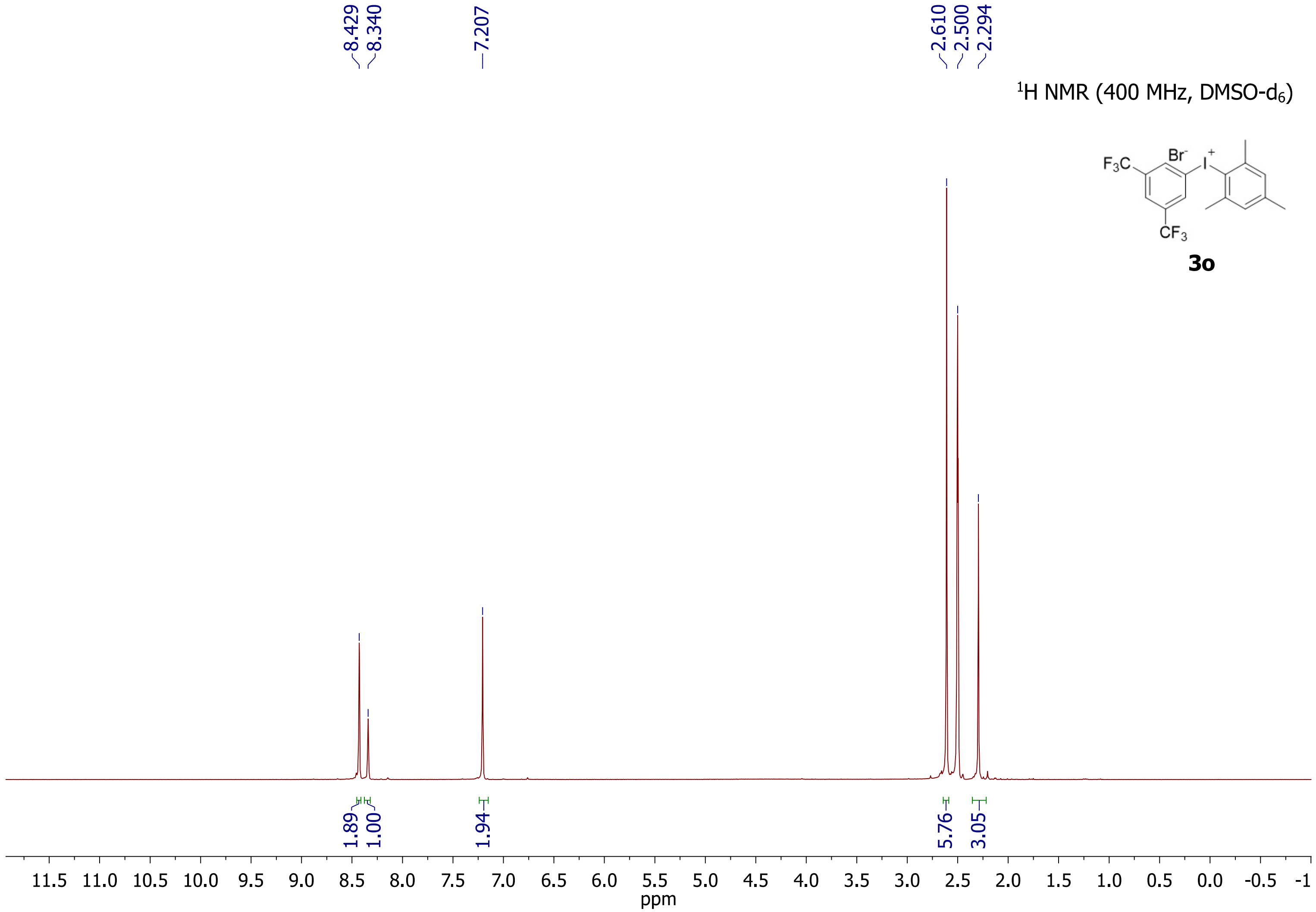

3o

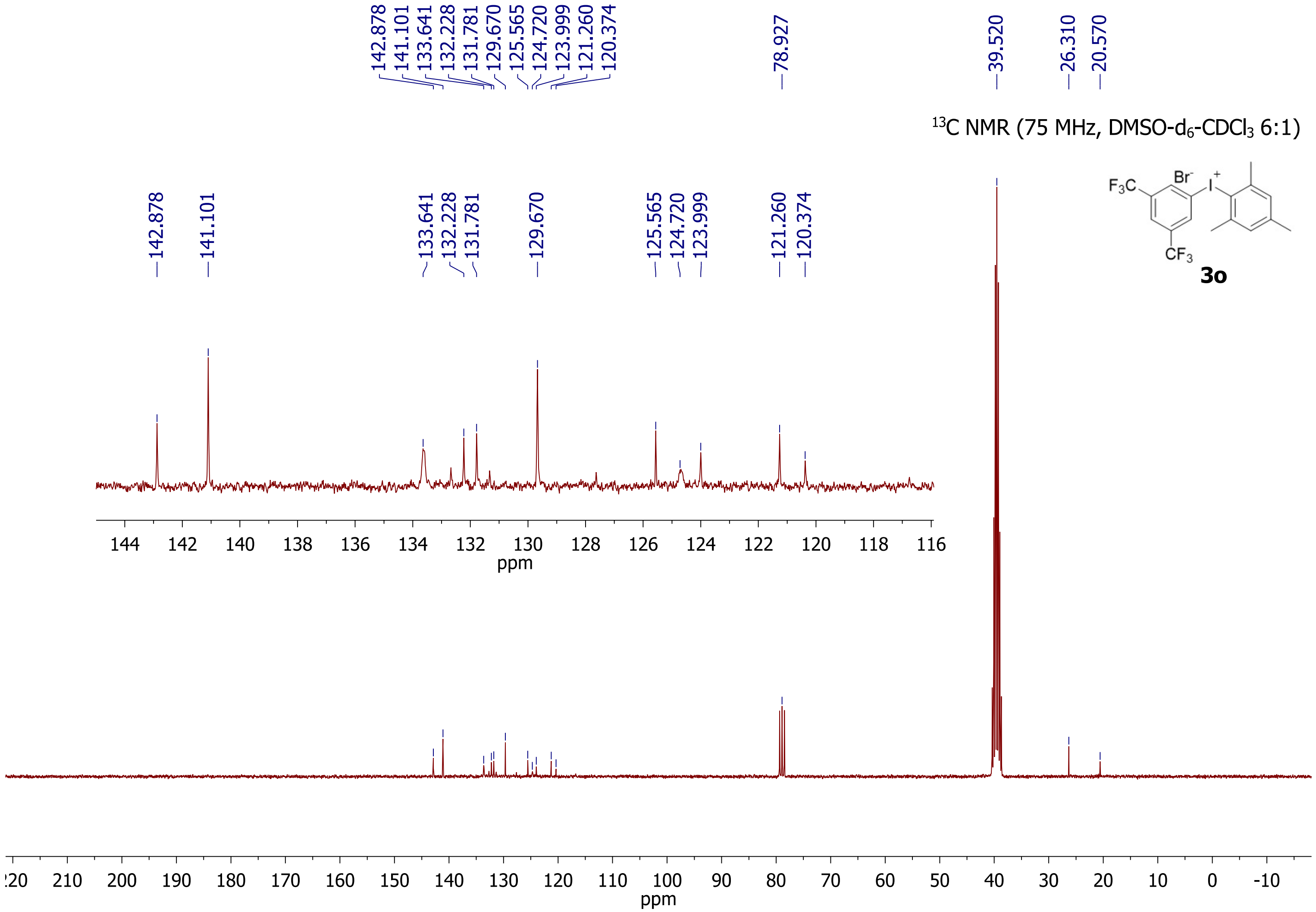

^{13}C NMR (75 MHz, DMSO-d_6-$CDCl_3$ 6:1)

3o

^{19}F NMR (376 MHz, DMSO-d_6)

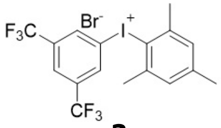

3o

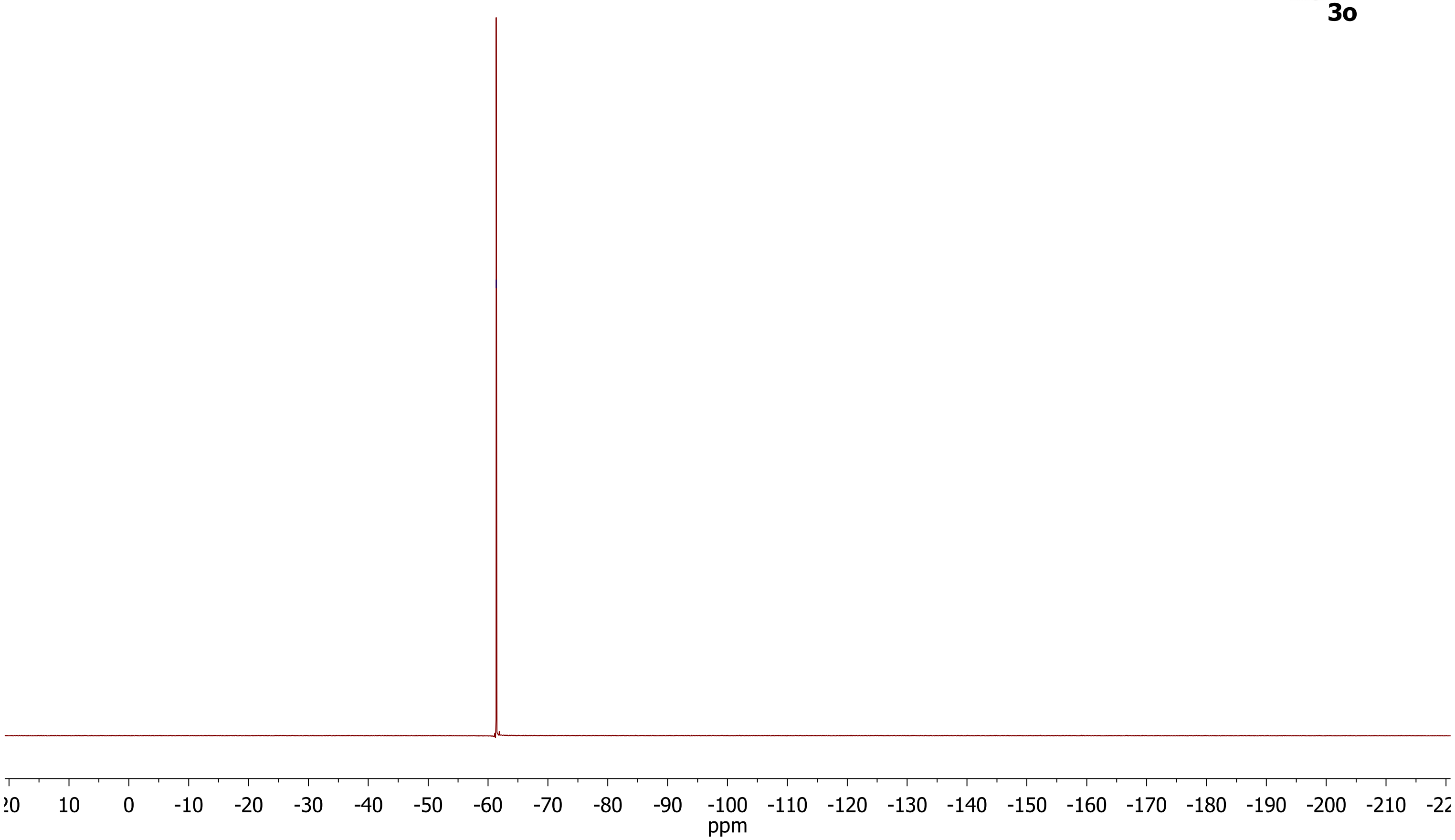

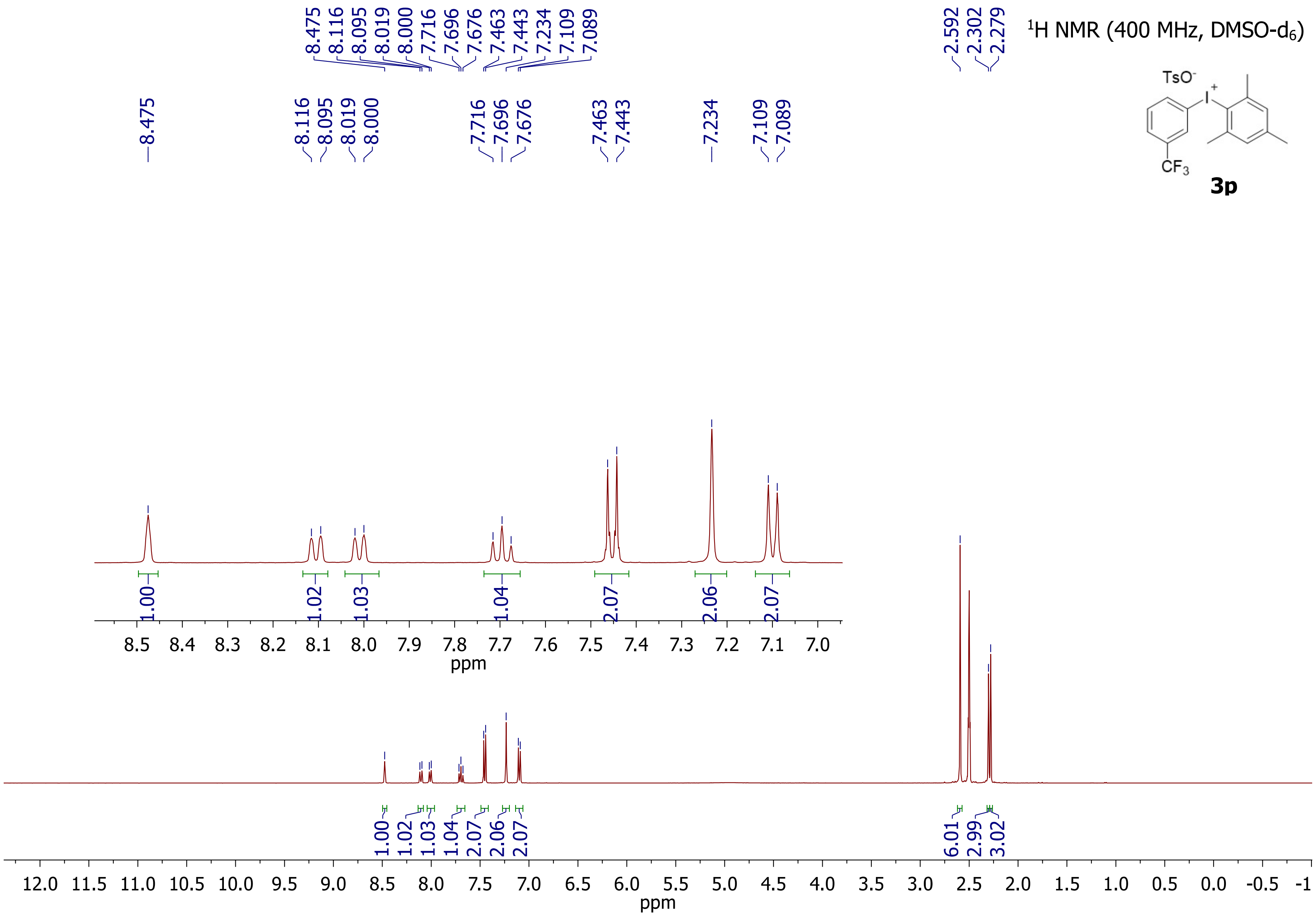

8.475
8.116
8.095
8.019
8.000
7.716
7.696
7.676
7.463
7.443
7.234
7.109
7.089
2.592
2.302
2.279
^1H NMR (400 MHz, DMSO-d_6)
TsO⁻
I⁺
CF_3
3p
1.00
1.02
1.03
1.04
2.07
2.06
2.07
6.01
2.99
3.02
ppm

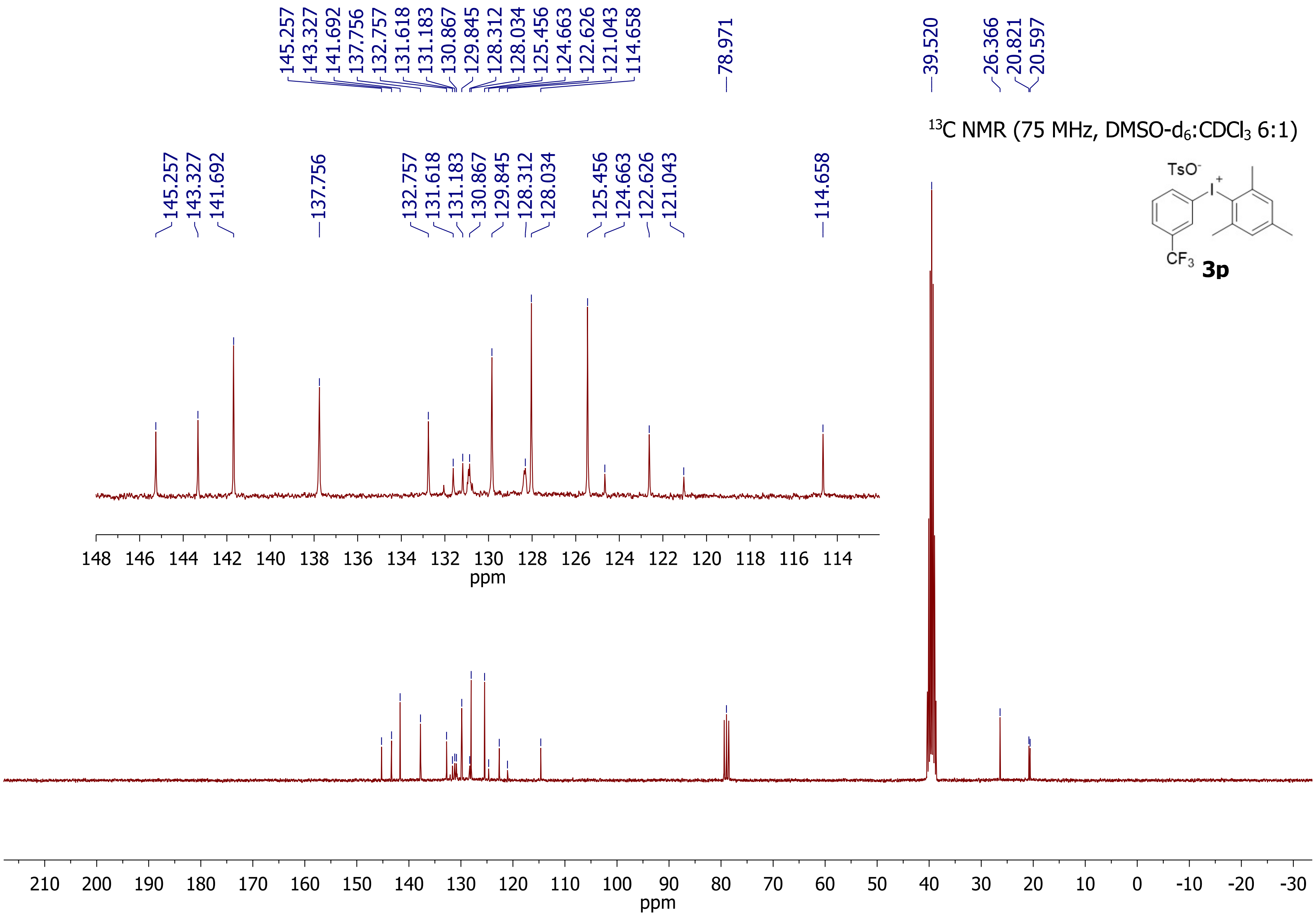
145.257
143.327
141.692
137.756
132.757
131.618
131.183
130.867
129.845
128.312
128.034
125.456
124.663
122.626
121.043
114.658
78.971
39.520
26.366
20.821
20.597
13C NMR (75 MHz, DMSO-d6:CDCl3 6:1)
TsO-
I+
CF3
3p
ppm
ppm

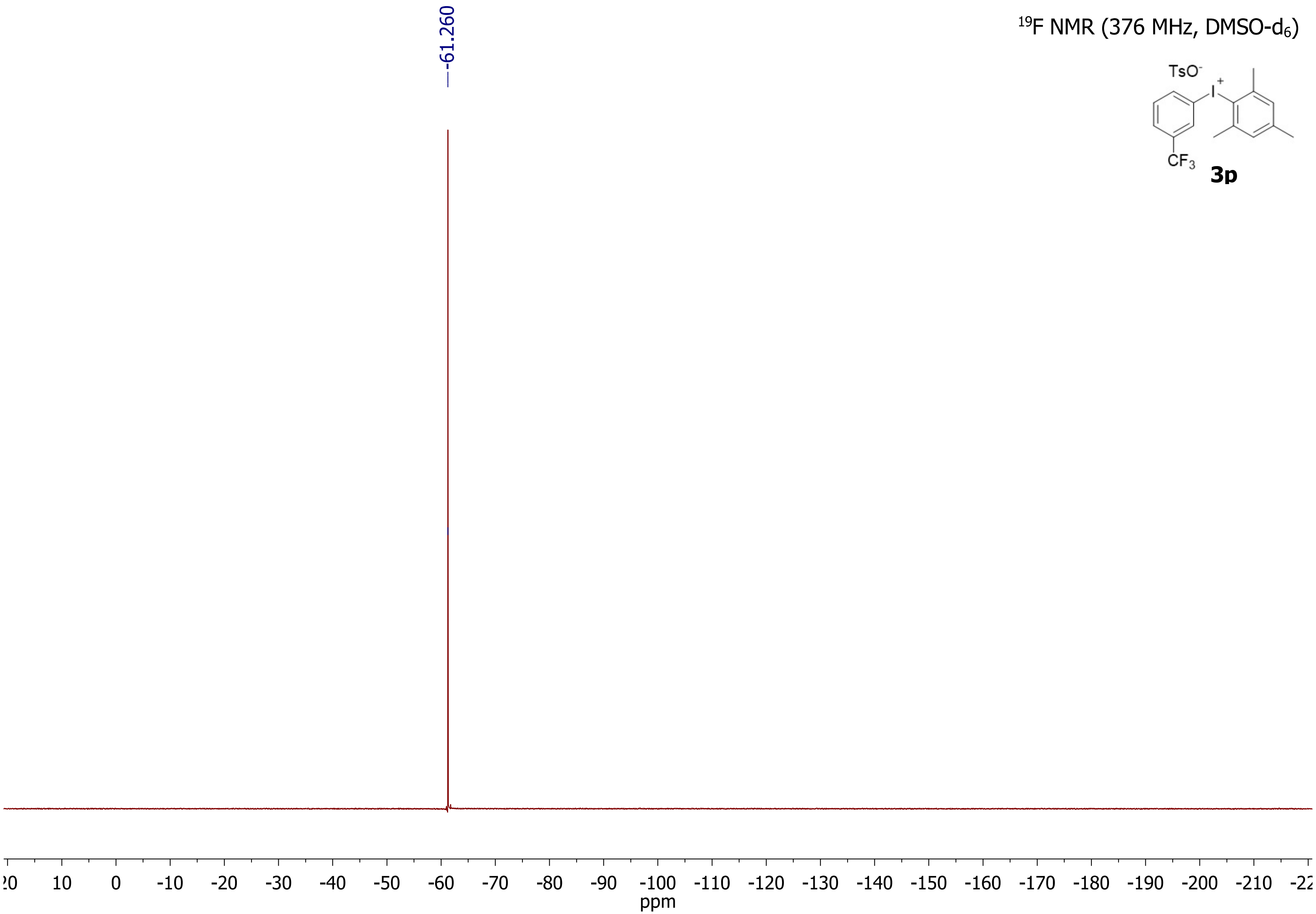

^{19}F NMR (376 MHz, DMSO-d_6)

3p

^1H NMR (400 MHz, $CDCl_3$)

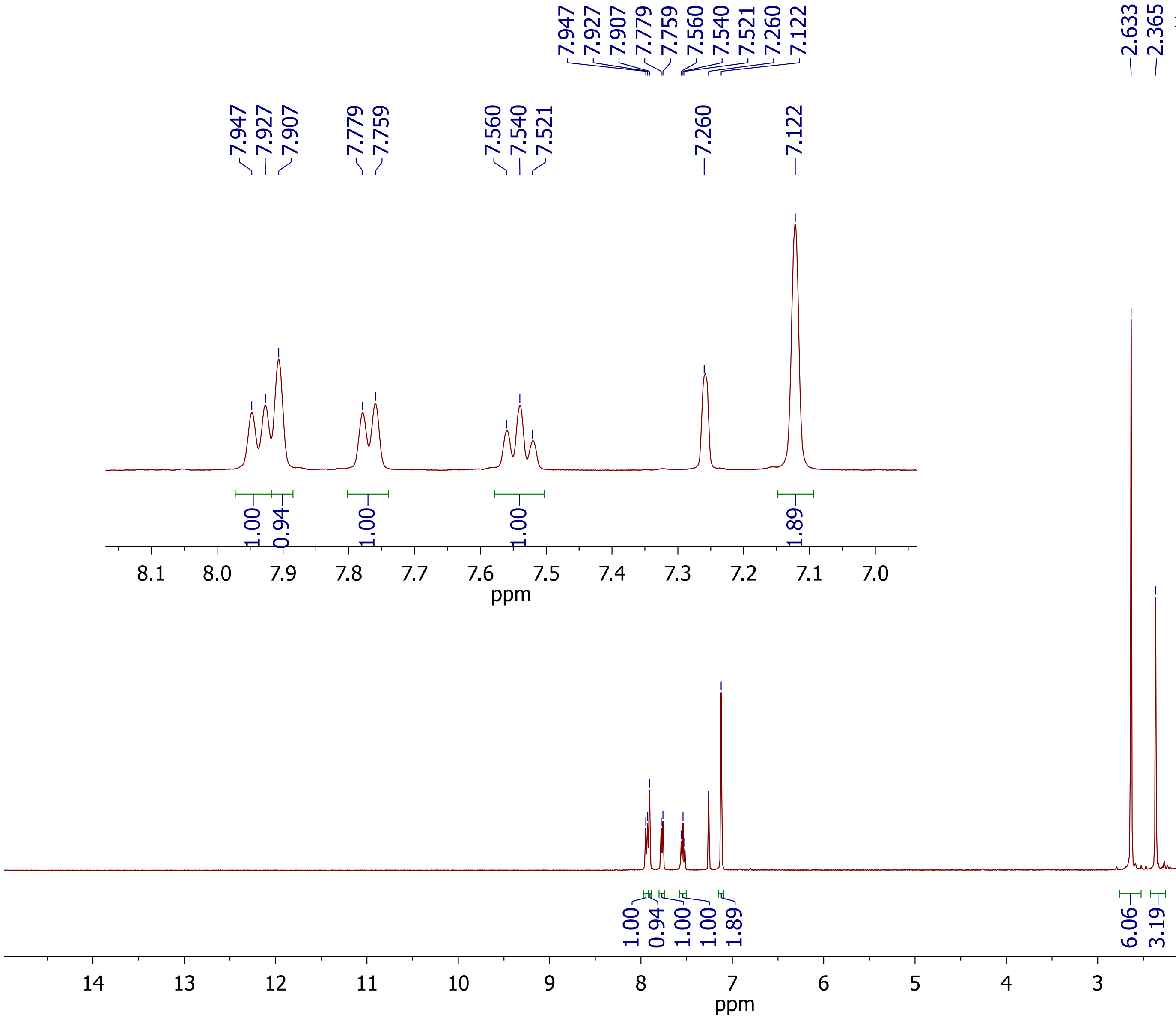

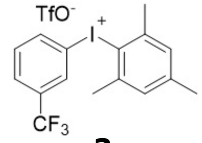

3q

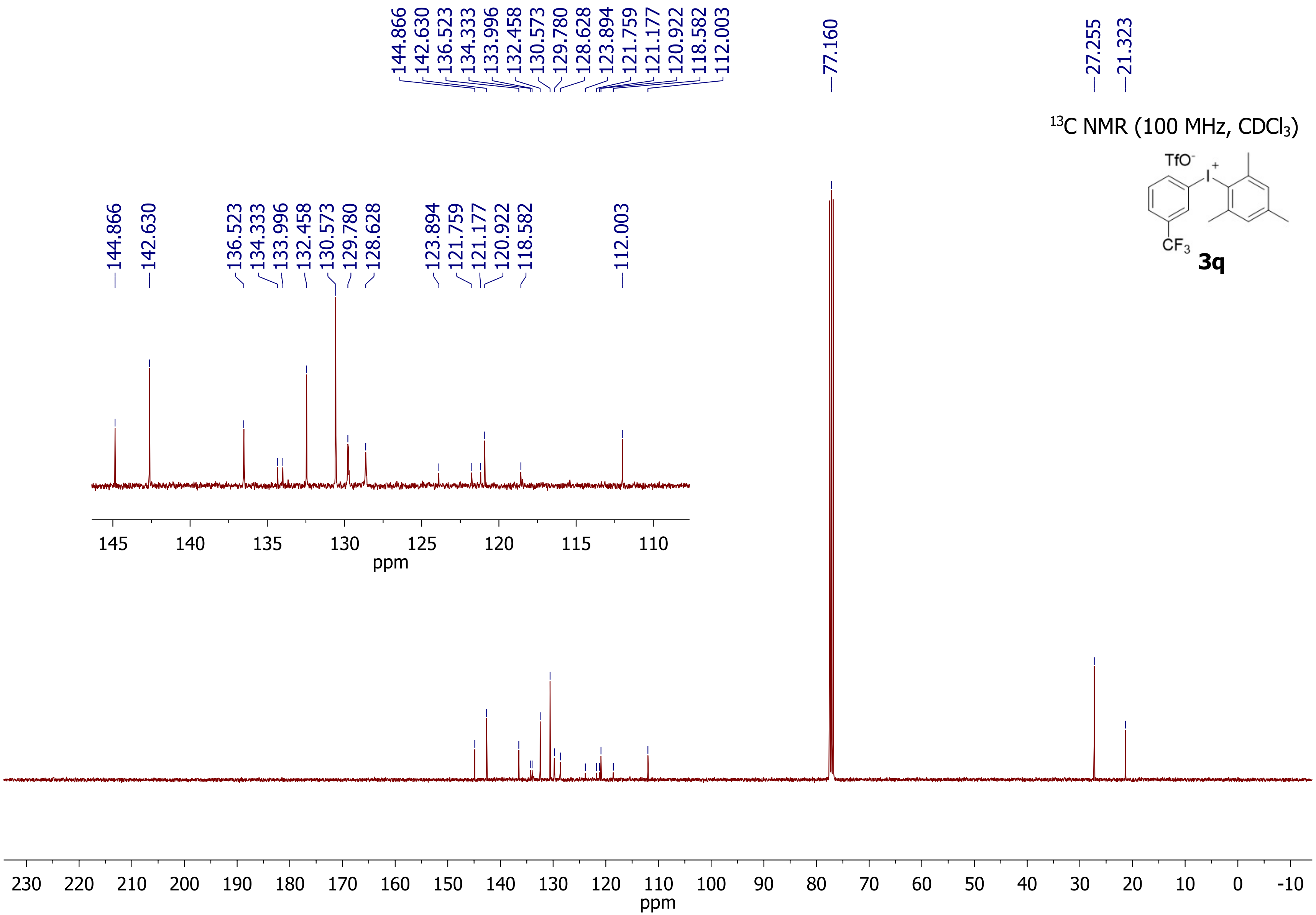

^{13}C NMR (100 MHz, CDCl$_3$)

3q

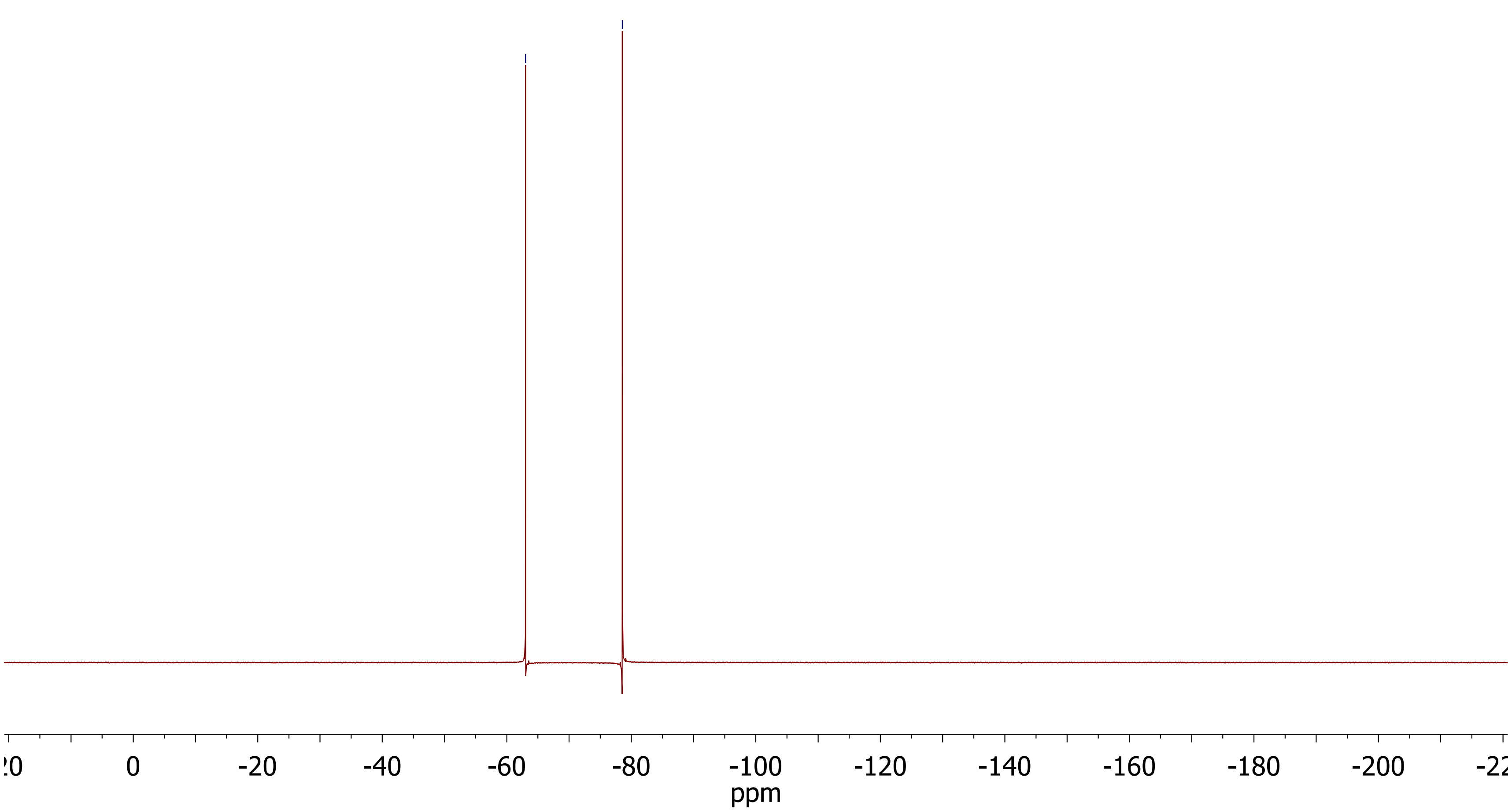

^{19}F NMR (376 MHz, $CDCl_3$)

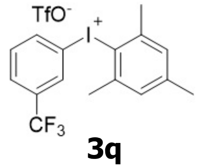

3q

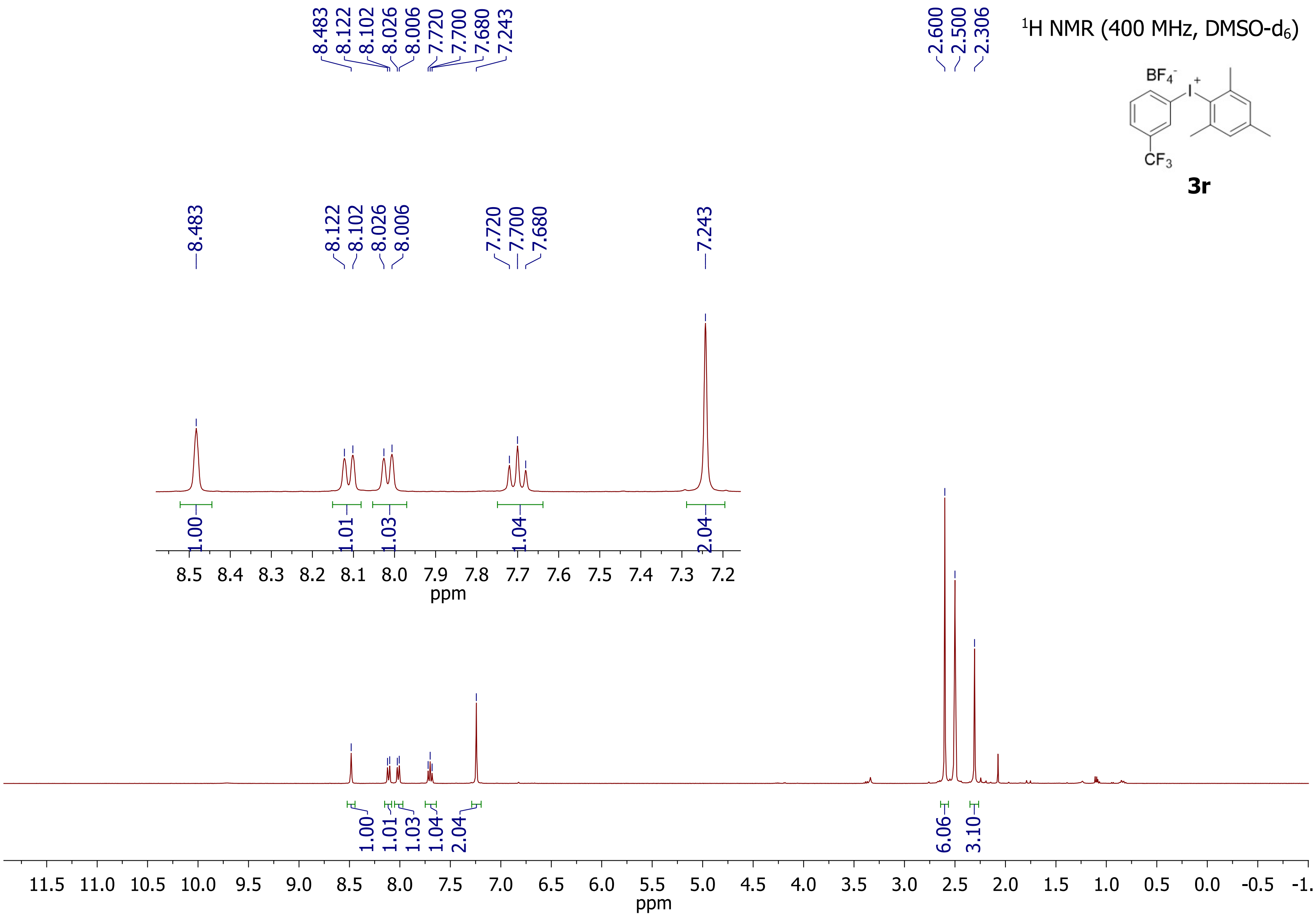

^1H NMR (400 MHz, DMSO-d_6)

3r

^{13}C NMR (75 MHz, $CDCl_3$)

3r

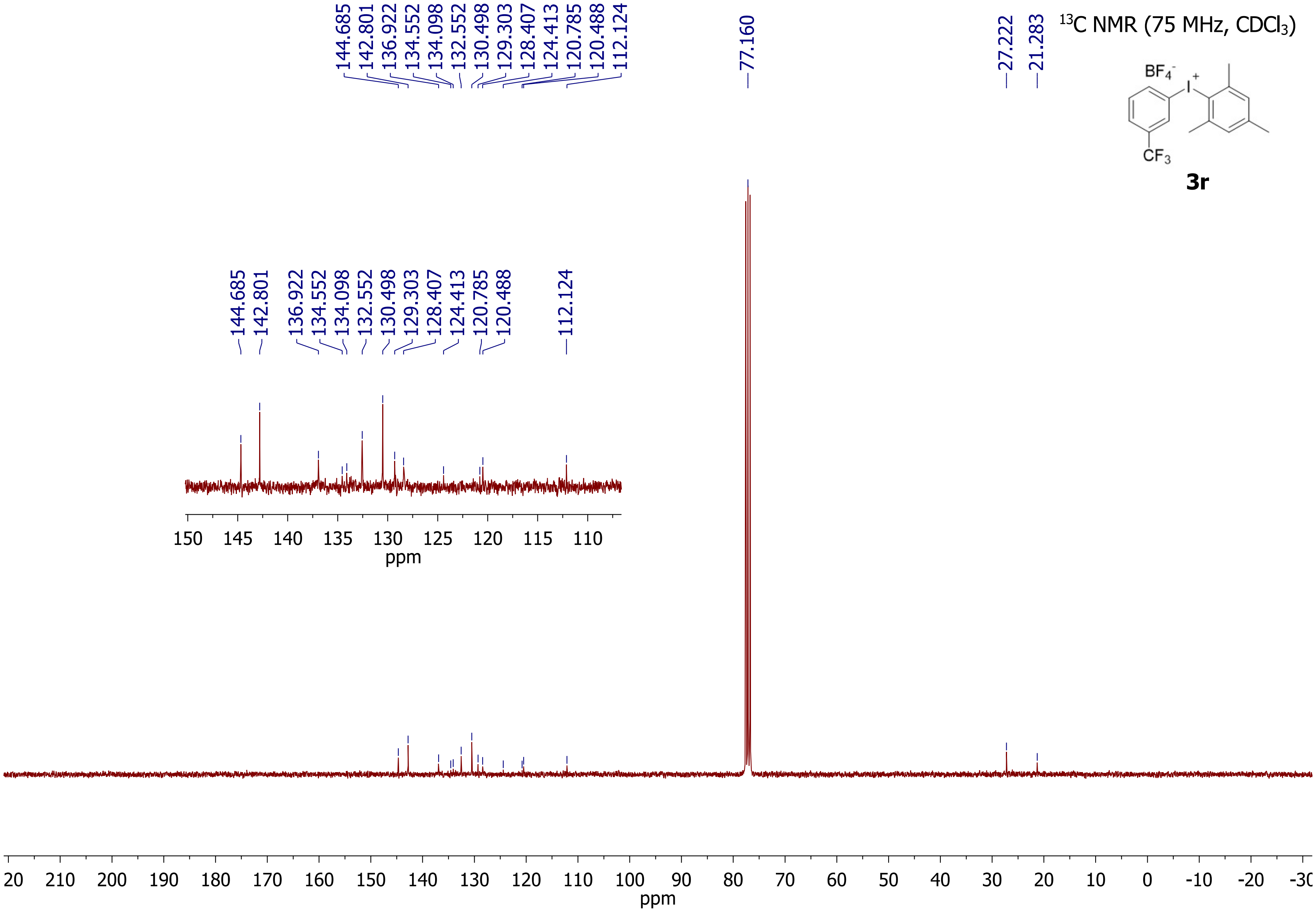

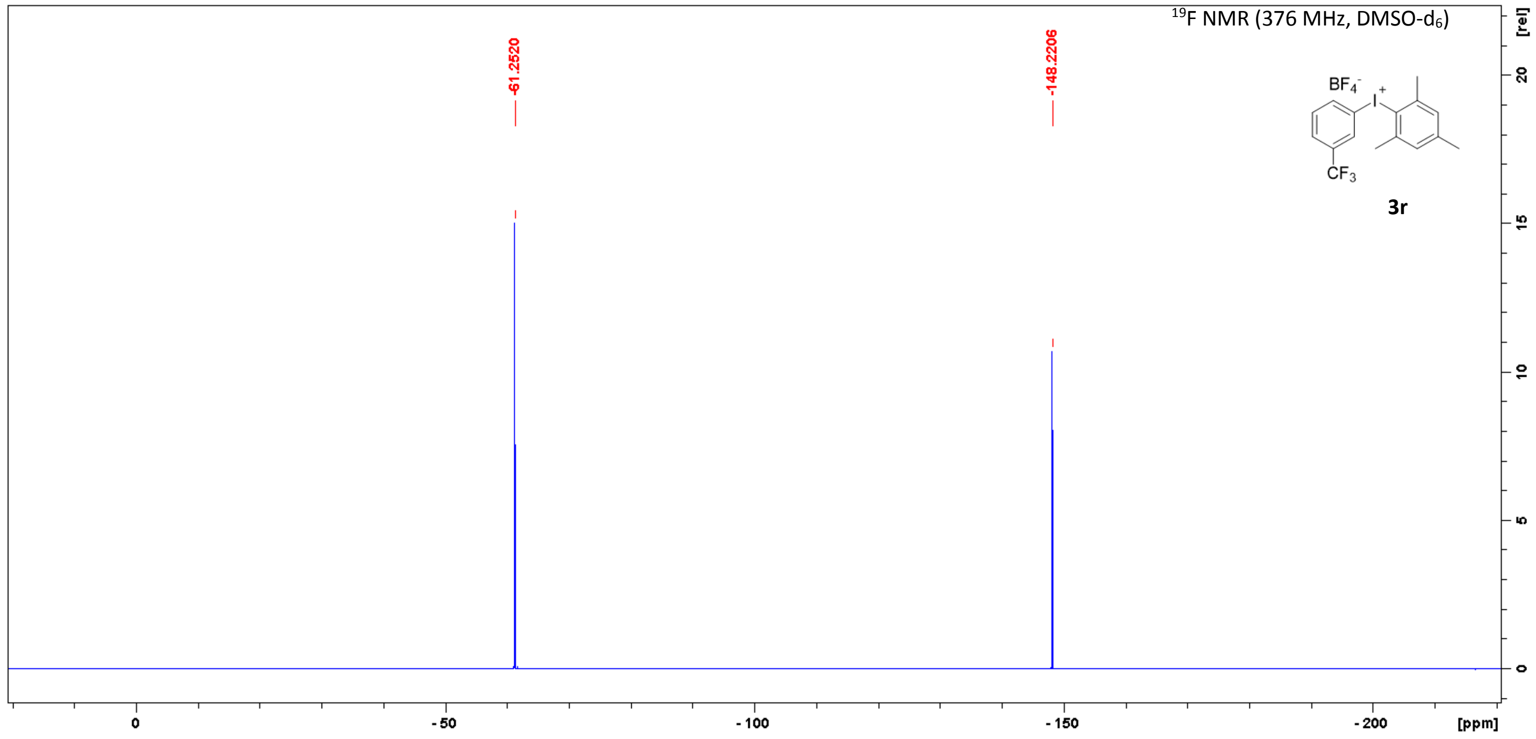
19F NMR (376 MHz, DMSO-d6)
-61.2520
-148.2206
BF4-
I+
CF3
3r
[rel]
20
15
10
5
0
0
- 50
- 100
- 150
- 200
[ppm]

13C NMR (75 MHz, DMSO-d6:CDCl3 6:1)

^{1}H NMR (400 MHz, DMSO-d_6)

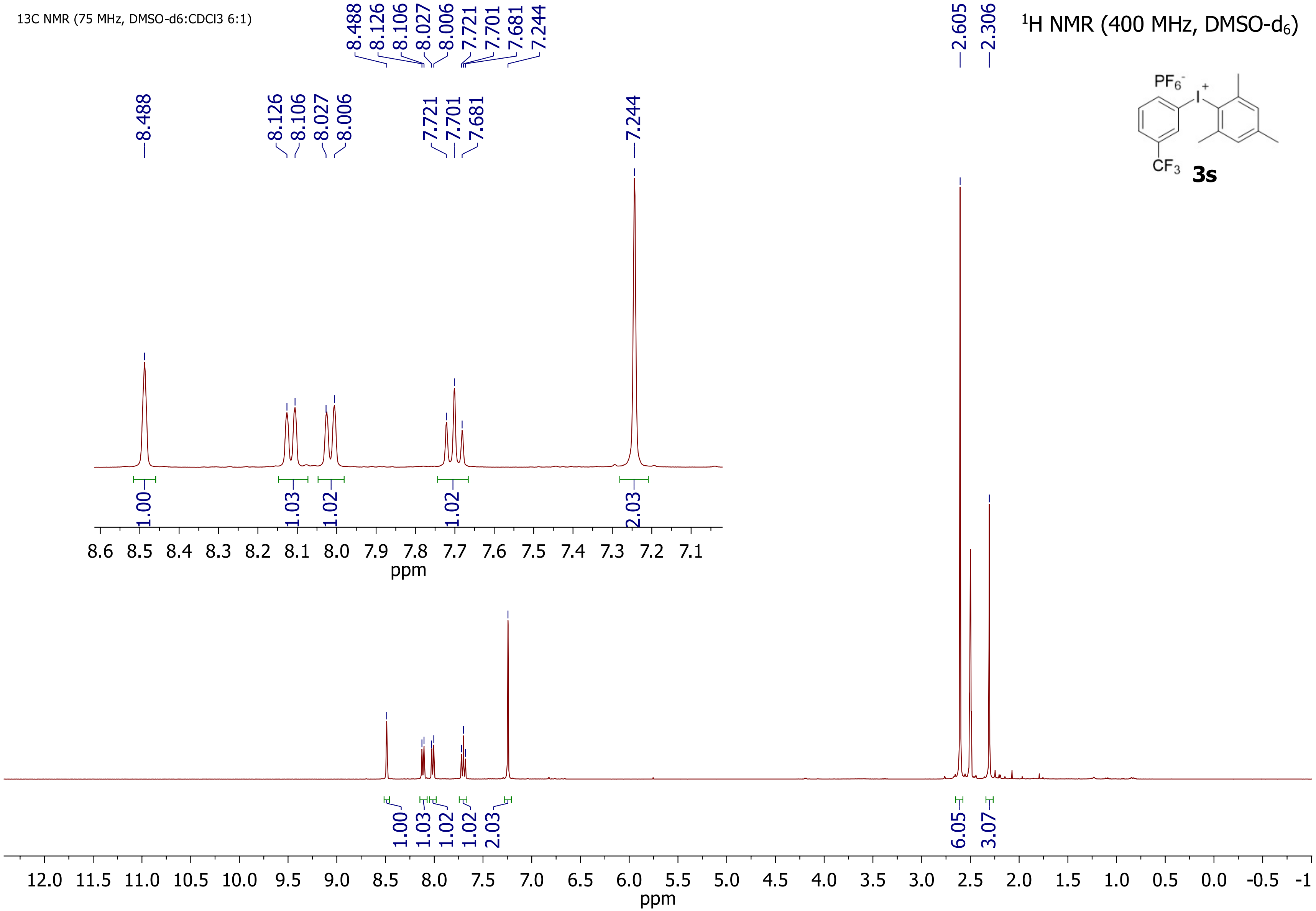

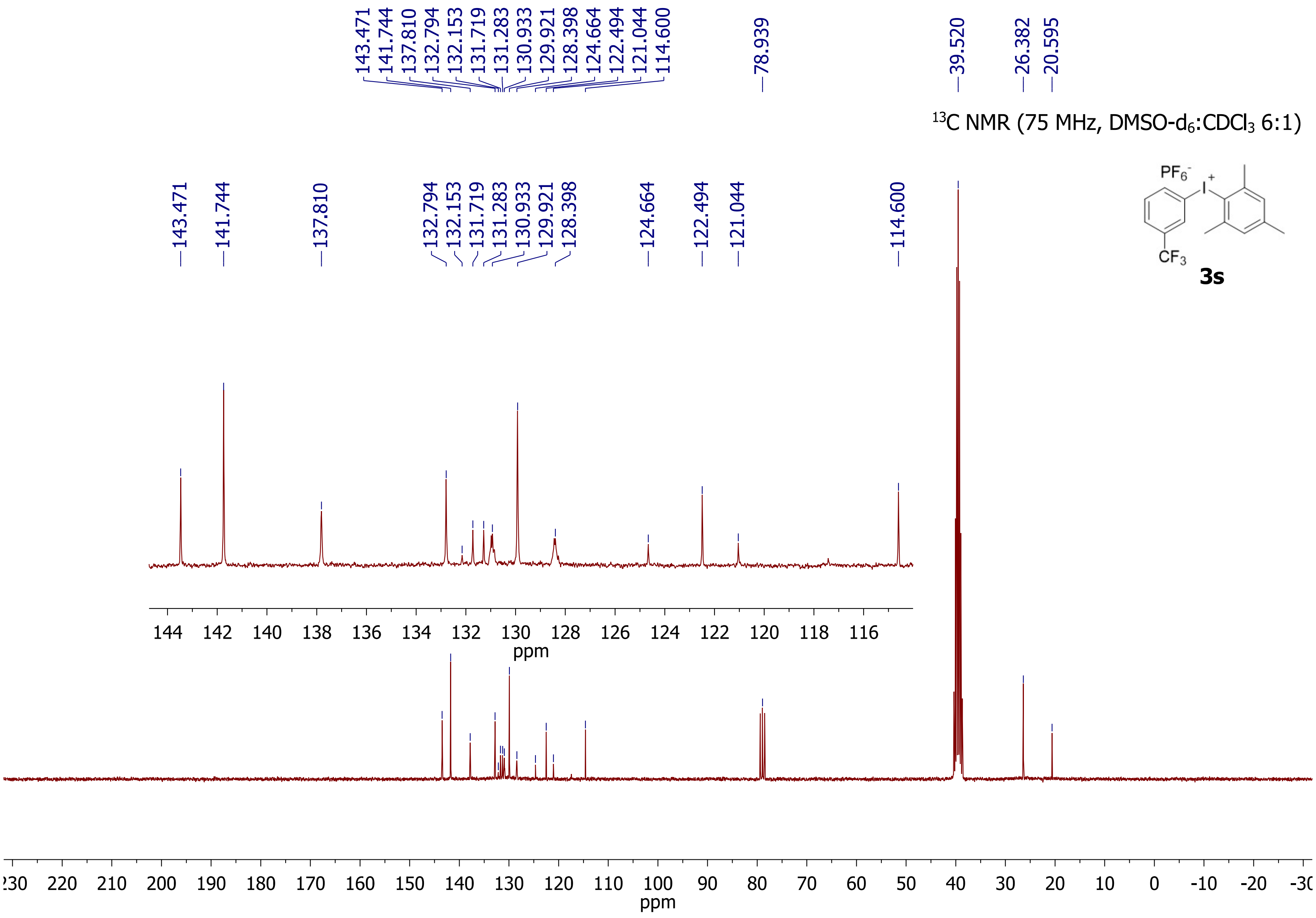
143.471
141.744
137.810
132.794
132.153
131.719
131.283
130.933
129.921
128.398
124.664
122.494
121.044
114.600
78.939
39.520
26.382
20.595
^{13}C NMR (75 MHz, DMSO-d_6:$CDCl_3$ 6:1)
PF_6^-
I^+
CF_3
3s
144 142 140 138 136 134 132 130 128 126 124 122 120 118 116
ppm
230 220 210 200 190 180 170 160 150 140 130 120 110 100 90 80 70 60 50 40 30 20 10 0 -10 -20 -30
ppm

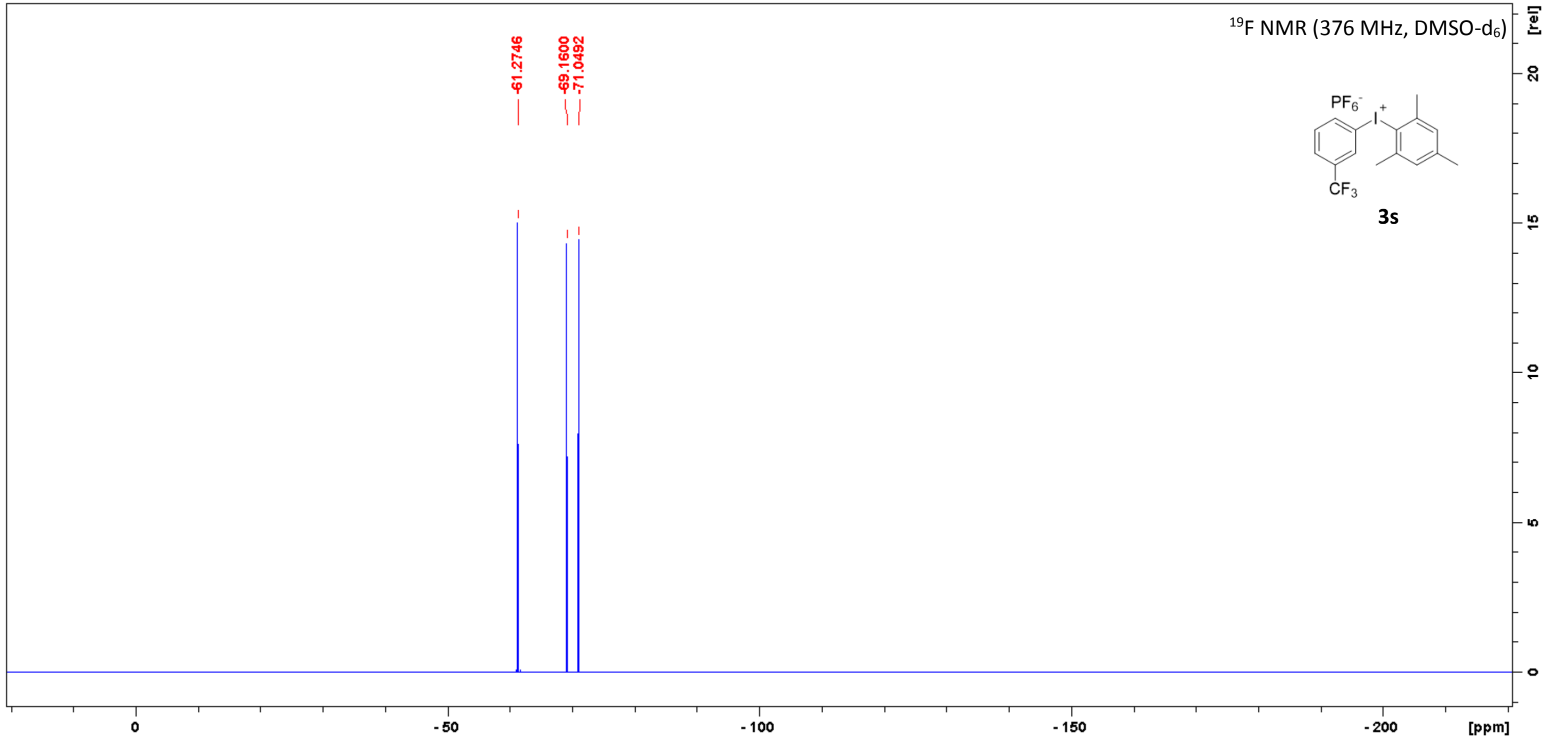
-61.2746
-69.1600
-71.0492
^{19}F NMR (376 MHz, DMSO-d$_6$)
PF$_6^-$
I$^+$
CF$_3$
3s
[rel]
20
15
10
5
0
0
- 50
- 100
- 150
- 200
[ppm]

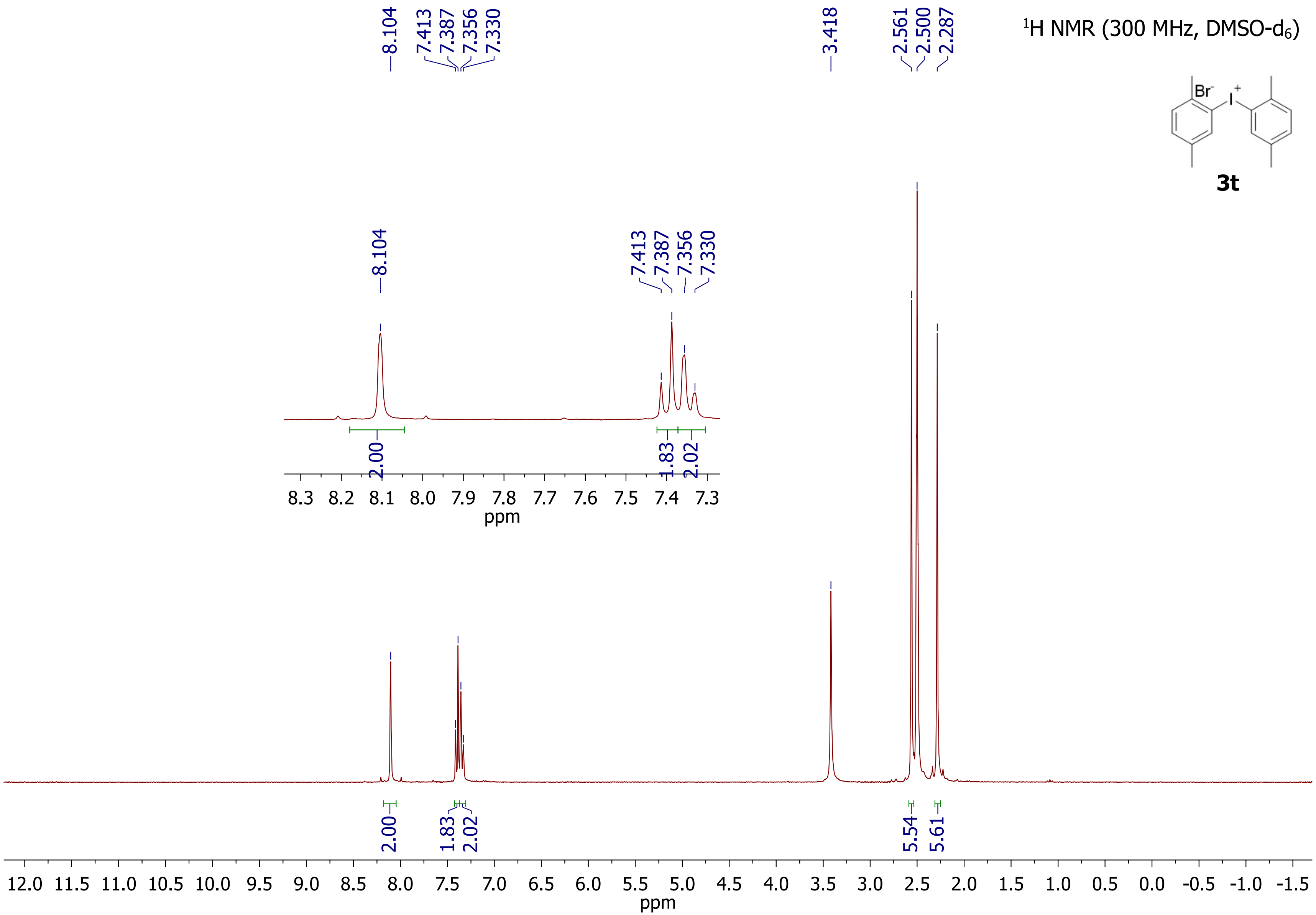

¹H NMR (300 MHz, DMSO-d6)
8.104
7.413
7.387
7.356
7.330
3.418
2.561
2.500
2.287
2.00
1.83
2.02
5.54
5.61
ppm
Br⁻
I⁺

3t

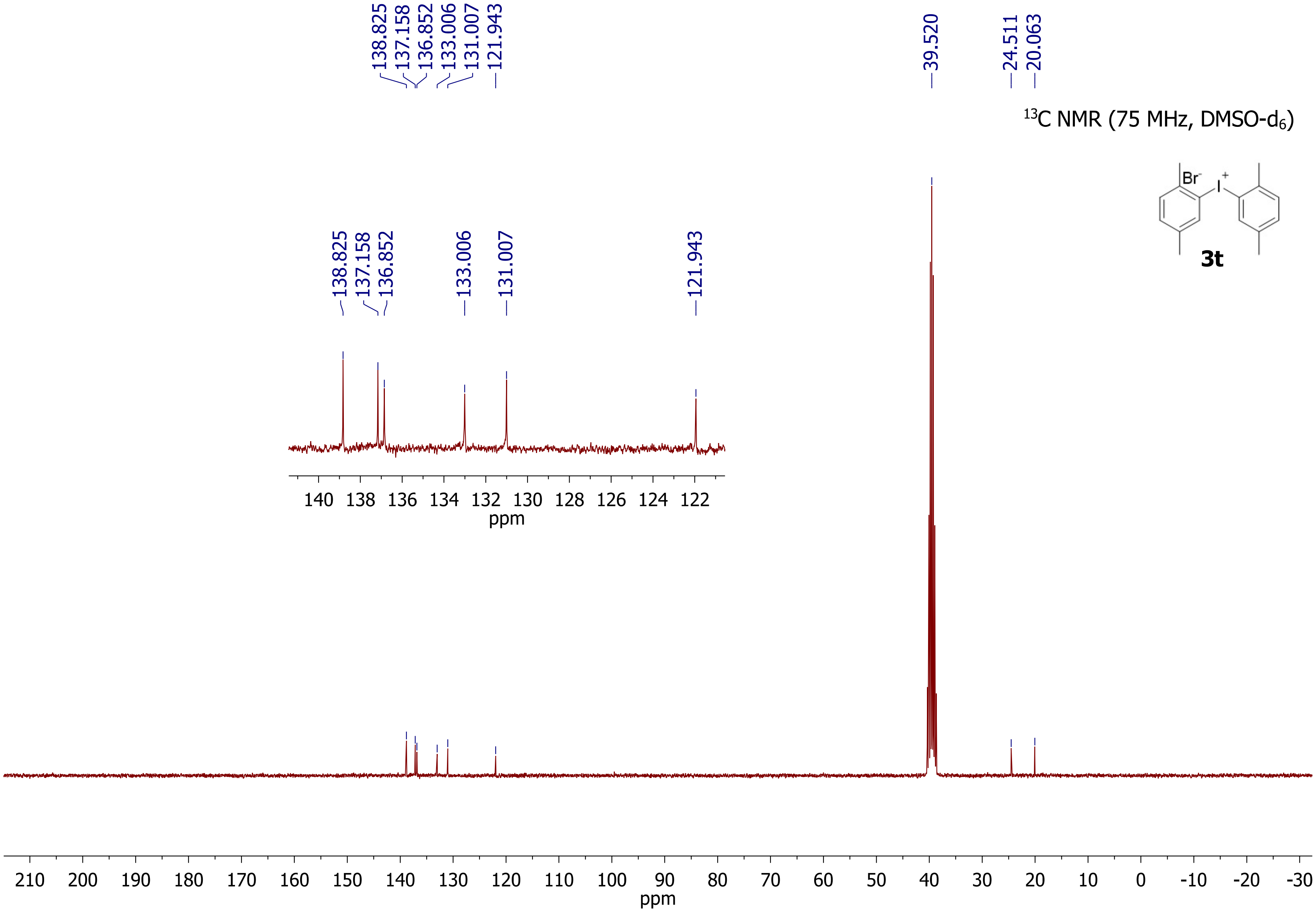
138.825
137.158
136.852
133.006
131.007
121.943
39.520
24.511
20.063
^{13}C NMR (75 MHz, DMSO-d_6)
Br⁻
I⁺
3t
ppm
ppm

^1H NMR (300 MHz, Methanol-d_4)

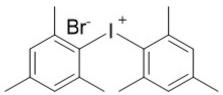

3u

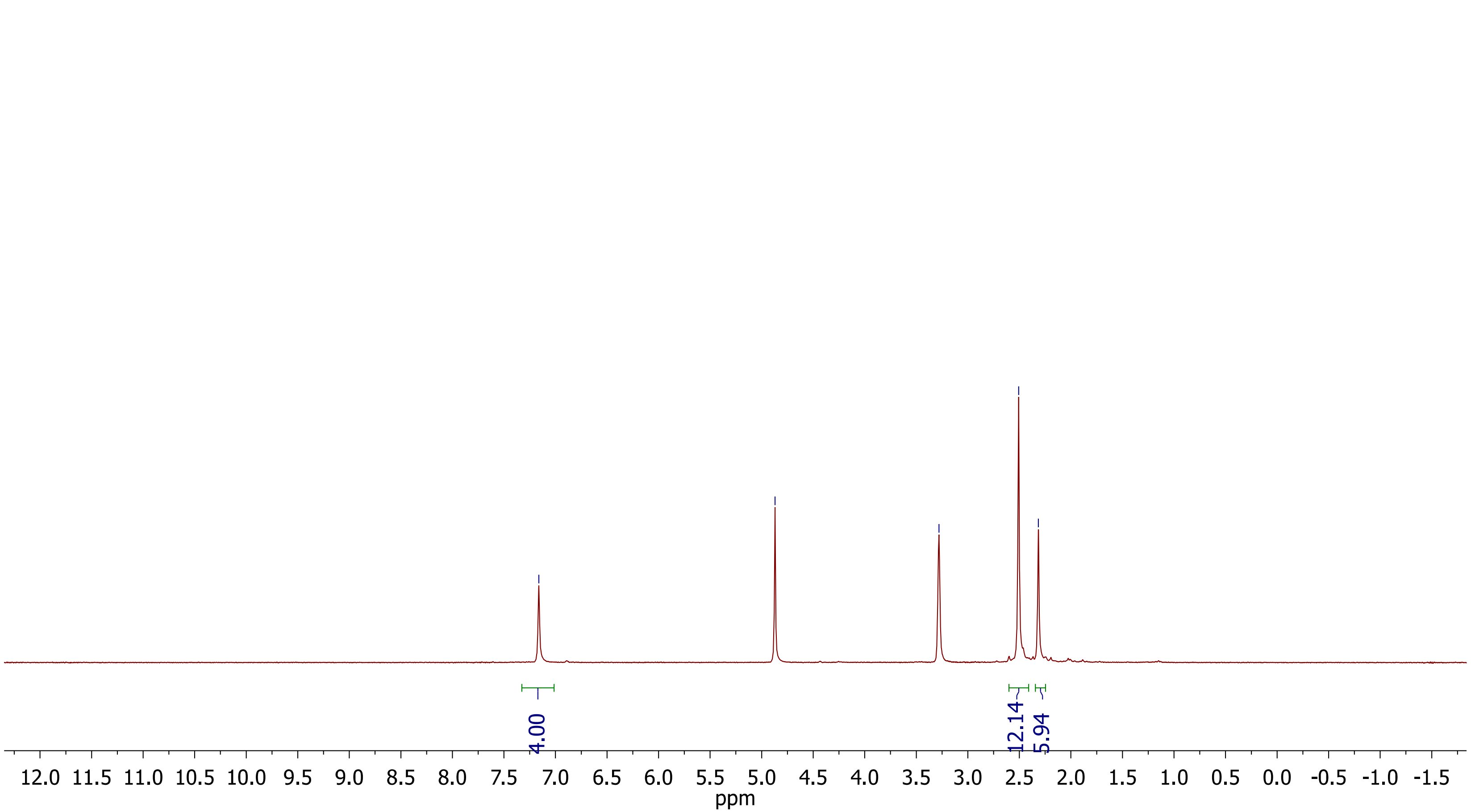

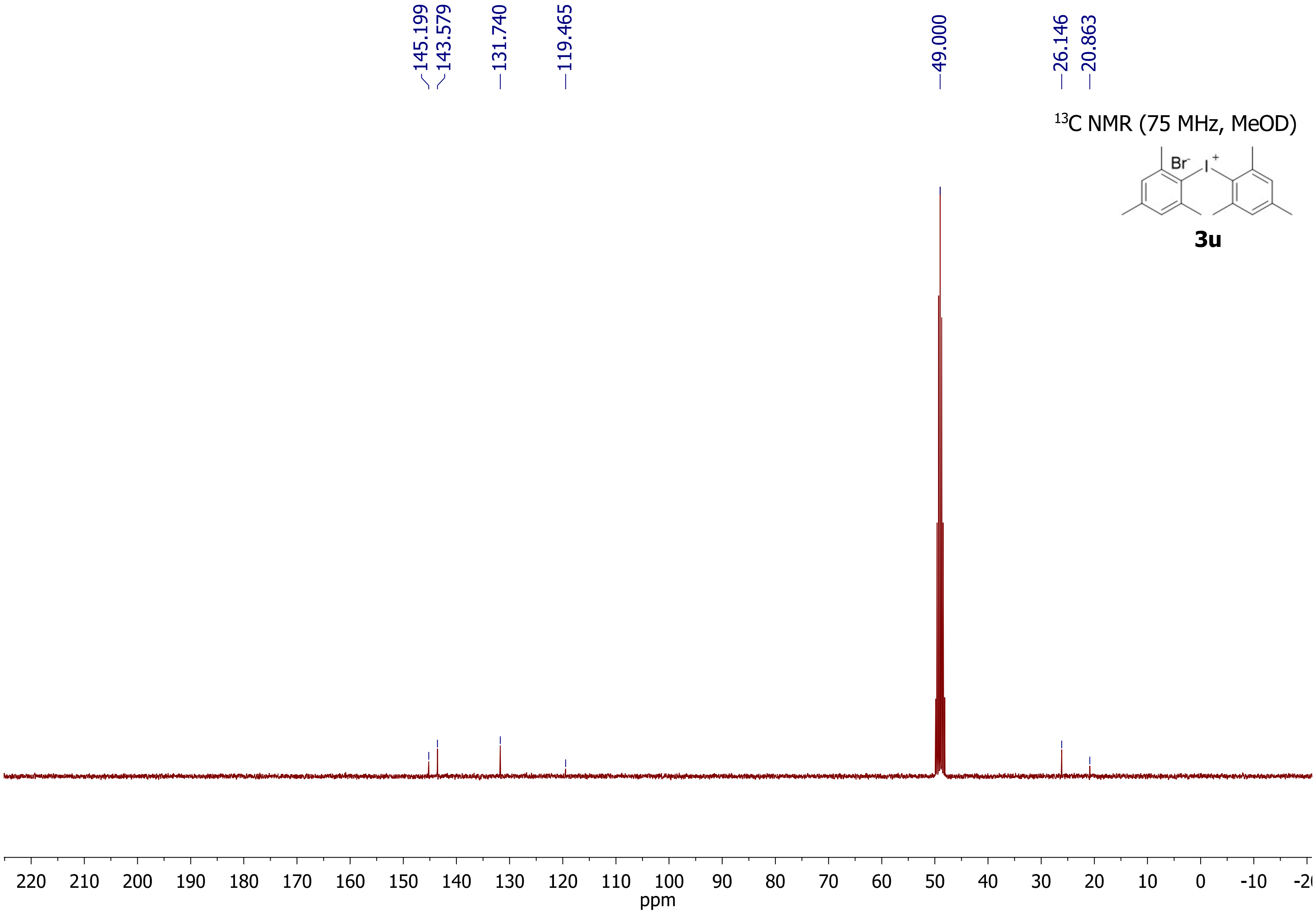

^{13}C NMR (75 MHz, MeOD)

3u

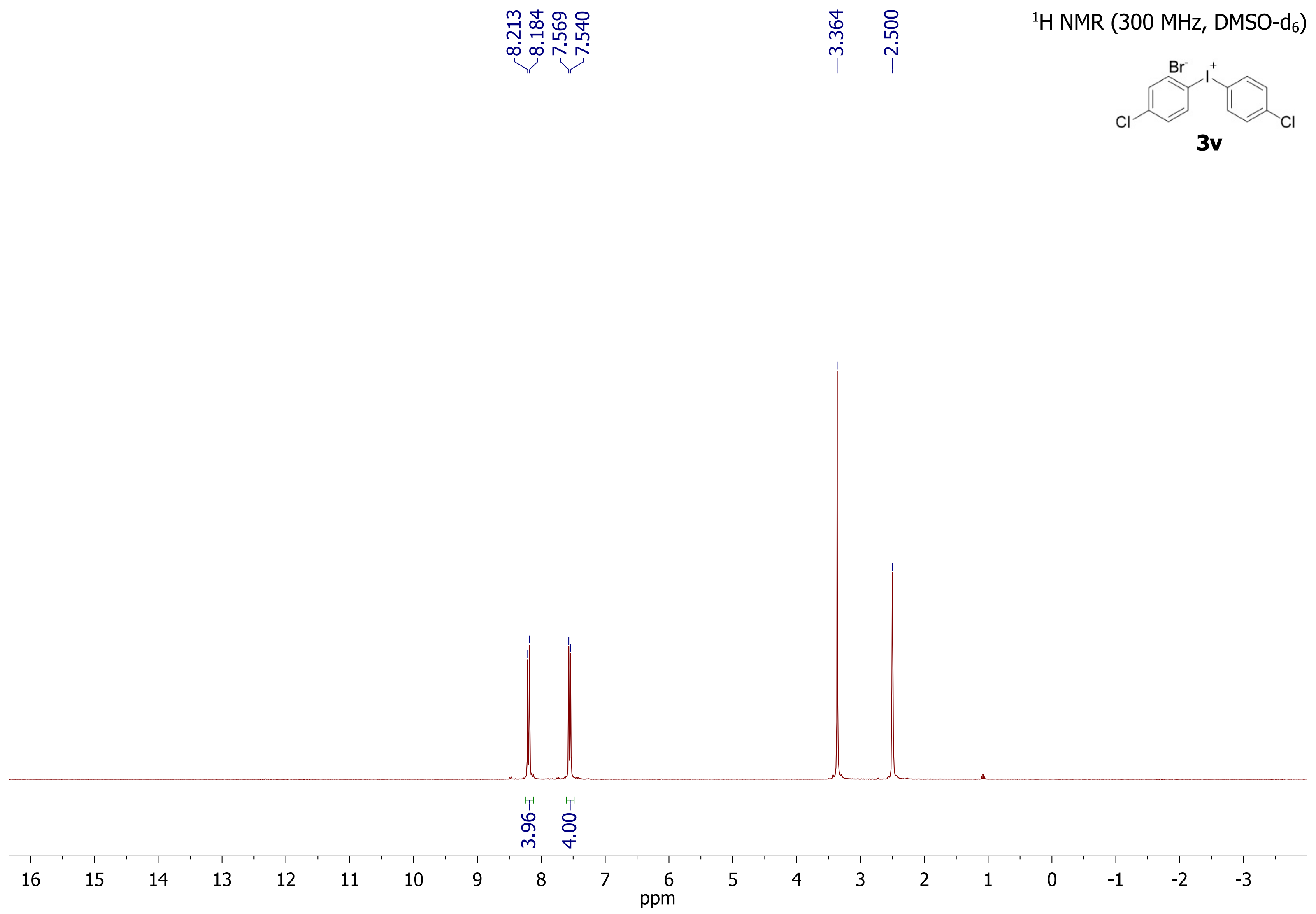

8.213
8.184
7.569
7.540
3.364
2.500
¹H NMR (300 MHz, DMSO-d6)
Br⁻
I⁺
Cl
Cl
3v
3.96
4.00
16 15 14 13 12 11 10 9 8 7 6 5 4 3 2 1 0 -1 -2 -3
ppm

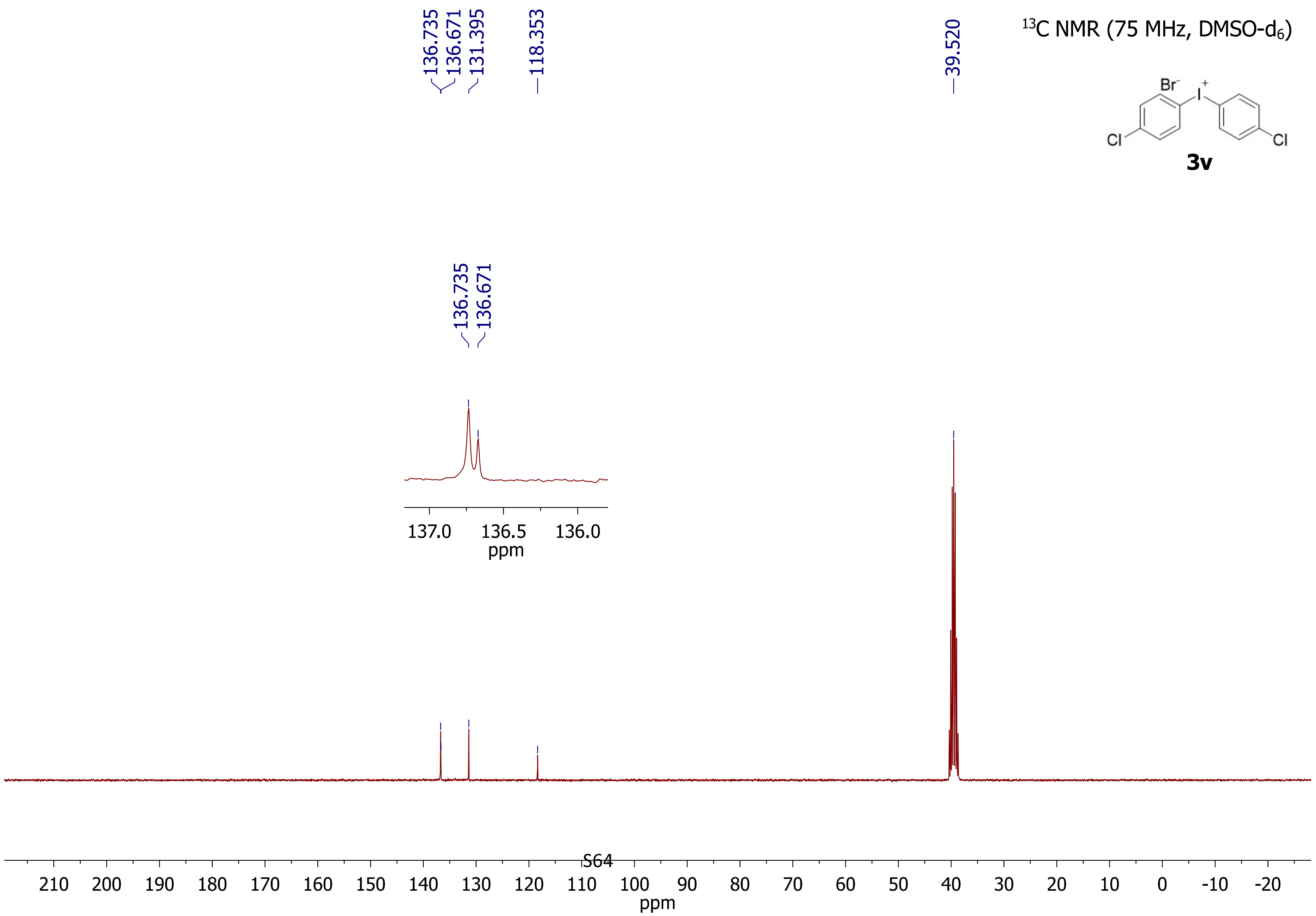

136.735
136.671
131.395
118.353
39.520
^{13}C NMR (75 MHz, DMSO-d$_6$)
Br⁻
I⁺
Cl
Cl
3v
136.735
136.671
137.0
136.5
136.0
ppm
210 200 190 180 170 160 150 140 130 120 110 100 90 80 70 60 50 40 30 20 10 0 -10 -20
ppm
